# *Mikania micrantha* genome provides insights into the molecular mechanism of rapid growth

**DOI:** 10.1038/s41467-019-13926-4

**Published:** 2020-01-17

**Authors:** Bo Liu, Jian Yan, Weihua Li, Lijuan Yin, Ping Li, Hanxia Yu, Longsheng Xing, Minling Cai, Hengchao Wang, Mengxin Zhao, Jin Zheng, Feng Sun, Zhenzhen Wang, Zhaoyang Jiang, Qiaojing Ou, Shubin Li, Lu Qu, Qilei Zhang, Yaping Zheng, Xi Qiao, Yu Xi, Yan Zhang, Fan Jiang, Cong Huang, Conghui Liu, Yuwei Ren, Sen Wang, Hangwei Liu, Jianyang Guo, Haihong Wang, Hui Dong, Changlian Peng, Wanqiang Qian, Wei Fan, Fanghao Wan

**Affiliations:** 1 Guangdong Laboratory of Lingnan Modern Agriculture, Shenzhen; Genome Analysis Laboratory of the Ministry of Agriculture; Agricultural Genomics Institute at Shenzhen, Chinese Academy of Agricultural Sciences, Shenzhen, 518120 China; 2 Key Laboratory of Agro-Environment in the Tropics, Ministry of Agriculture and Rural Affairs; Guangdong Provincial Key Laboratory of Eco-Circular Agriculture; College of Natural Resources and Environment, South China Agricultural University, Guangzhou, 510642 China; 3 Institute of Ecological Science, Guangdong Provincial Key Laboratory of Biotechnology for Plant Development; Guangzhou Key Laboratory of Subtropical Biodiversity and Biomonitoring; School of Life Science, South China Normal University, Guangzhou, 510631 China; 4 Key Laboratory of Protein Function and Regulation in Agricultural Organisms of Guangdong province, College of Life Science, South China Agricultural University, Guangzhou, 510642 China; 5 The Institute of Plant Protection, Chinese Academy of Agricultural Sciences, Beijing, 100193 China; 6 Fairy Lake Botanical Garden, Shenzhen and Chinese Academy of Sciences, Shenzhen, 518004 China

**Keywords:** DNA sequencing, Comparative genomics, Genome evolution, Plant ecology, Plant physiology

## Abstract

*Mikania micrantha* is one of the top 100 worst invasive species that can cause serious damage to natural ecosystems and substantial economic losses. Here, we present its 1.79 Gb chromosome-scale reference genome. Half of the genome is composed of long terminal repeat retrotransposons, 80% of which have been derived from a significant expansion in the past one million years. We identify a whole genome duplication event and recent segmental duplications, which may be responsible for its rapid environmental adaptation. Additionally, we show that *M. micrantha* achieves higher photosynthetic capacity by CO_2_ absorption at night to supplement the carbon fixation during the day, as well as enhanced stem photosynthesis efficiency. Furthermore, the metabolites of *M. micrantha* can increase the availability of nitrogen by enriching the microbes that participate in nitrogen cycling pathways. These findings collectively provide insights into the rapid growth and invasive adaptation.

## Abstract (web summary)

*Mikania micrantha* is an extremely fast-growing invasive plant species that can cause serious damage to natural ecosystems. Here, the authors assemble its chromosome-scale reference genome and explore possible mechanisms that contribute to its rapid growth.

## Introduction

M*ikania micrantha* Kunth (“mile-a-minute” weed) is an extremely fast-growing, sprawling, perennial vine belonging to the family Asteraceae and native to tropical America^1^. The vine is listed as one of the top 100 worst invasive species by the International Union for Conservation of Nature (IUCN)^2^. It is problematic in tropical Asia, in parts of Papua New Guinea, on Indian Ocean islands and Pacific Ocean islands and in Florida in the United States^3,4^, causing serious damage to the natural ecosystems^1,5^. The invasion of *M. micrantha* has caused economic losses related to forests and crop production of up to US$4000 ha^−1^ ^6,7^ by climbing plants to the canopy, and blocking the sunlight and has led to a loss of genetic and species diversity, a decline in soil and food web stability, and altered nutrient cycling^4,8–11^.

*M. micrantha* possesses a variety of biological characteristics associated with successful invasive plant species. It germinates early in the growing season; grows extremely fast (with a maximum mean growth rate of 20 cm day^−1^)^12^; roots from each vine node; has a smothering habit; produces very large numbers of widely dispersed seeds (170,000 m^−2^)^13^, which are very small (8.92 × 10^−5^ g 1000 grains^−1^)^14,15^; and has an ability to survive harsh conditions^15^. *M. micrantha* also exhibits a high degree of morphological and physiological plasticity in response to different light environments^16^. In addition, *M. micrantha* has strong allelopathic effects on other plants and soil microbes^10,17,18^. Allelopathy is the influence of a plant over a target species, through the release into the environment of compounds that influence the growth and development of biological systems^19^. Various allelochemicals, that are produced by a plant must escape into the environment and subsequently influence the growth and development of other plants, such as *Mikania* sesquiterpene lactones (STLs)^20–22^ and phenolic compounds^23^ have been isolated from different *M. micrantha* plant tissues. However, the molecular mechanism underlying the fast growth of *M. micrantha* and the biosynthetic pathways of its characteristic allelochemicals are not yet clear.

Asteraceae is not only one of the largest, most valuable plant families, but also contains the largest number of invasive alien species worldwide. Genomes of some important species of Asteraceae, including lettuce^24^, sunflower^25^, artichoke^26^, and horseweed^27^, have been sequenced, deepening our understanding of Asteraceae evolution and promoting biological studies of plants in Asteraceae. Although some studies have examined the biological characteristics of *M. micrantha*, genomic and molecular data are scarce, which limits in-depth studies of this invasive species. Here, we generate a reference genome of *M. micrantha* and perform analyses of transcriptomic, metabolomics, and metagenomic data to explore possible reasons causing its rapid growth.

## Results

### Chromosome-level genome assembly and recent LTR-RT expansion

**Fig. 1:**
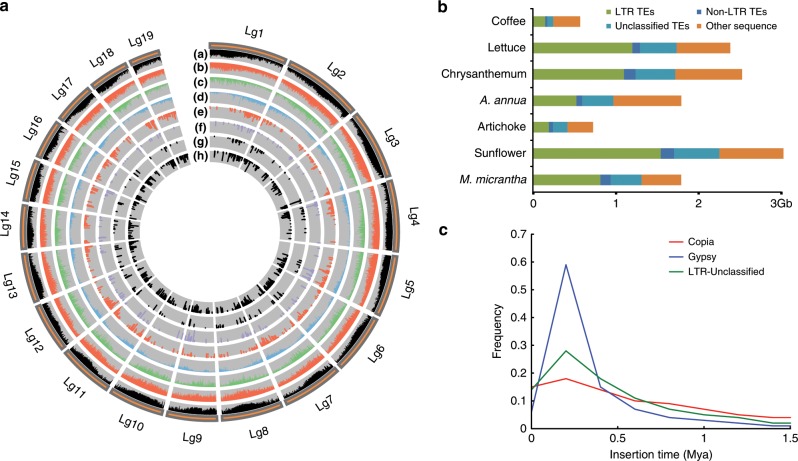
Landscape of the *Mikania micrantha* genome. **a** Integration of genomic and expression data. (a) Distribution of GC content; (b) distribution of the Gypsy family of long terminal repeats retrotransposons (LTR-RTs); (c) distribution of the Copia family of LTR-RTs; (d) distribution of coding genes; (e–h) expression of organ-specific genes (from outside to inside tracks: root, stem, leaf and flower). **b** Comparison of repetitive sequences in asterids, including *Mikania micrantha*, sunflower (*Helianthus annuus*), artichoke thistle (*Cynara cardunculus*), *Artemisia annua*, chrysanthemum (*Dendranthema morifolium*), lettuce (*Lactuca sativa*), and coffee (*Coffea canephora*). **c** The insertion time distribution of intact LTRs in the *M. micrantha* genome. Mya indicates million years ago.

We generated 228 Gb (122 × coverage) of single molecule real-time (SMRT) sequences on the PacBio RS II and Sequel platform (Supplementary Table 1); these sequences were used to assemble a haploid reference genome by Canu-1.6, followed by filtering of the extra heterozygous fragments (Supplementary Fig. 1 and Supplementary Note 1). The assembled genome includes 4414 contigs with a total length of 1.79 Gb, a contig N50 of 1352 kb, and a contig N90 of 180 Kb (Table 1 and Supplementary Table 2). Based on the distribution of k-mer frequencies, the estimated genome size of *M. micrantha* is 1.86 Gb (Supplementary Fig. 2); thus, 96% of the *M. micrantha* genome has been successfully assembled. With Hi-C technology, 1.61 Gb (90.3%) of contigs was anchored and oriented into the 19 linkage groups (Fig. 1a and Supplementary Table 2), with the longest being 124 Mb and the shortest being 47 Mb (Table 1).

**Table 1:** Statistics of the *Mikaina micrantha* genome and gene prediction.

	Number	Size
Assembly feature		
Estimated genome size		1.86 Gb
Assembled sequences	4414	1.79 Gb
N50 contigs		1.35 Mb
Linkage group	19	1.62 Gb
N50 linkage group		93.11 Mb
Longest linkage group	1	124.36 Mb
Anchored and oriented sequences	1618	1.61 Gb
Gaps	1599	159.9Kb
Unanchored scaffolds	2796	173.72 Mb
GC content		36.2%
Genome annotation		
Total repetitive sequence		1.36 Gb
Gene models	46,351	57.56 Mb
lncRNAs	3633	41.10 Mb

The protein-coding genes in the reference genome were predicted by the EVidenceModeler pipeline (Supplementary Table 3). In total, 46,351 gene models were predicted in the assembled genome as the reference gene set, with coding regions spanning ~57.6 Mb (3.2%) of the genome (Table 1 and Supplementary Table 4). In the evaluation of completeness, 91% of eukaryote core genes from OrthoDB (http://www.orthodb.org) were identified as complete in the reference gene set by BUSCO (Table 1), comparable to the number for other published genomes (Supplementary Fig. 3). A total of 42,804 (92%) coding proteins were annotated by functional databases (Supplementary Table 5), including eggNOG, KEGG, Interpro, and uniprot. In addition, we identified 3633 long noncoding RNA (lncRNAs) classified into three subclasses: 2489 intergenic lncRNAs (lincRNAs), 551 intronic lncRNAs (ilncRNAs), and 593 antisense lncRNAs (NAT-lncRNAs) (Supplementary Figs. 4–6).

The high coverage and continuity of the *M. micrantha* genome enabled a comprehensive analysis of transposable elements (TEs). In total, more than three quarters of the *M. micrantha* genome consists of TEs (Fig. 1b and Supplementary Table 6), 60% of which are long terminal repeat retrotransposons (LTR-RTs). Notably, the most abundant LTR-RTs family present in *M. micrantha* was *Gypsy*, accounting for 70.1% of all LTR elements, followed by *Copia* (26.1%) (Supplementary Table 7). LTR-RTs are the most abundant DNA components in all investigated plant species and are largely responsible for plant genome size variation. Interestingly, over 80% of the LTR-RT lineages in *M. micrantha* are very young, exhibiting minimal sequence divergence compared to those of other plant species, owing to significant expansion in the past 1 million years (Fig. 1c), which is consistent with previous reports in sunflower^25^.

### Genome-wide duplication and subsequent fragment duplication

**Fig. 2:**
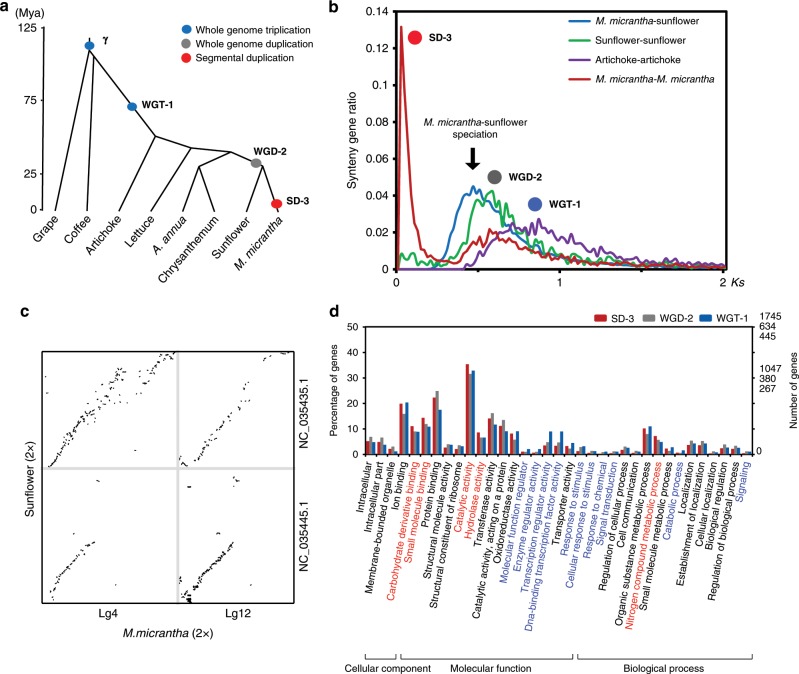
Genome evolution of *Mikania micrantha*. **a** The paleohistory of the asterids, including *M. micrantha*, sunflower, lettuce, artichoke, chrysanthemum, *Artemisia annua*, and coffee, with grape as an outgroup. A whole-genome duplication event (WGD-2) occurring before *M. micrantha-*sunflower divergence (approximately 32–36 Mya), and the shared ancestral whole genome triplications WGT-γ (approximately 122–164 Mya) and WGT-1 (approximately 53–62 Mya). Circles indicate the ages of WGT-1 (blue), WGD-2 (gray) and SD-3 (red). **b** *K*_*s*_ distribution of syntenic orthologues from *M. micrantha*, sunflower and artichoke. The *y*-axis shows the ratio of gene pairs in the syntenic block. The polyploidization (WGT-1, WGD-2, and SD-3) and speciation (*M. micrantha*-sunflower) events are referenced on the peaks. **c** Dot plots of syntenic orthologues in two chromosomes between *M. micrantha* and sunflower. **d** The functional enrichment of WGT-1, WGD-2, and SD-3 duplicated genes in *M. micrantha* by Gene Ontology (GO) classification. The significantly enriched GO terms for the SD-3 and WGT-1/WGT2 duplicated genes are marked by red and blue, respectively.

To assess the paleohistory of the asterids, we performed a comparative genomic investigation of *M. micrantha* with sunflower (*Helianthus annuus*), lettuce (*Lactuca sativa*), artichoke thistle (*Cynara cardunculus*), chrysanthemum (*Dendranthema morifolium*), *Artemisia annua* and coffee (*Coffea canephora*) with grape (*Vitis vinifera*) as an outgroup, showing that the *M. micrantha* diverged from grape ~109 million years ago (Mya), from artichoke ~51 Mya, and from lettuce ~43 Mya. Notably, *M. micrantha* and sunflower were diverged ~31 Mya (Fig. 2a).

To study the conservation of genomic structure, we identified the interspecies syntenic genes between *M. micrantha* and sunflower, artichoke, and grape, and intraspecies syntenic genes within the *M. micrantha*, sunflower, lettuce, artichoke, and coffee genomes, then calculated the *K*_*s*_ values of the syntenic fragments for orthologous pairs (Fig. 2b, c and Supplementary Figs. 7 and 8). These results, together with the results of previous reports^25^, showed that the *M. micrantha* genome has a complex paleo-polyploidy history, including the ancient whole-genome triplication WGT-γ (approximately 122–164 Mya) in dicotyledons, the whole-genome triplication WGT-1 (approximately 53–62 Mya) shared with asterids II, and the whole-genome duplication WGD-2 (approximately 32–36 Mya) shared with sunflower. In contrast to the genomes of other species in Asteraceae, the *M. micrantha* genome exhibits a large number of recent segmental duplications. With *K*_*s*_ values lower than 0.2, we identified 127 syntenic blocks ranging from 46 to 8955 kb in length and containing 23% of all syntenic genes, indicating that the *M. micrantha* genome experienced a recent explosion of lineage-specific segmental duplications (SD-3, less than 1 Mya; Fig. 2b).

Large-scale genome duplications produce abundant duplicated genes, which are important for the diversity of gene functions and adaptation to changing environments. The WGT-1, WGD-2, and SD-3 duplicated genes were significantly enriched in the functions of catalytic and hydrolase activity, nitrogen compound metabolism, transcription factor, response to stimulus and chemical (Fig. 2d and Supplementary Figs. 9 and 10). It demonstrated that extensive gene fractionation has occurred during the evolutionary history of the *M. micrantha* genome, which retained the genes that are essential for survival and lost other redundant genes. Next, gene families related to fast growth were analyzed. A total of 189 photosynthesis related genes were identified in the *M. micrantha* genome, and the number of genes related to photoreaction was significantly higher than that in other Asteraceae species (*p* < 0.01, Z-test; Supplementary Fig. 11). The large-scale duplication prompted photosynthesis gene expansion, with 5% of these genes produced after the WGT-1 and WGD-2 events and 21% of these genes produced after recent SD-3 events. In addition, auxin signaling and jasmonic acid (JA) biosynthesis genes were significantly expanded in the *M. micrantha* genome, compared with the genomes of other Asteraceae species (*p* < 0.01, Z-test; Supplementary Fig. 12a, b). The duplicated genes, which were enriched in categories of genes encoding interacting products, photosynthesis, hormone, and response to stress, play an important role in the environmental adaptability of plants.

### High photosynthesis of the leaves benefits rapid growth

In this study, the diurnal carbon isotope (δ^13^C) changes in *M. micrantha* leaves were not significantly different between day and night based on analysis of the carbon isotope ratio (Supplementary Fig. 13), indicating that *M. micrantha* has a typical C_3_ carbon assimilation type, consistent with the findings of previous reports^1^. However, the net photosynthetic rate of leaves was higher than that in other C_3_ plants and similar to that in C_4_ plants^1^. Previous studies revealed that all plants contain the genes necessary for Crassulacean acid metabolism (CAM) photosynthesis, but the genes regulating the CAM pathway are often silent or inactivated in non-CAM plants^28^. However, the expression of genes related to CAM photosynthesis is activated in response to changes in the external environment or in certain growth stages^29,30^. To adapt to environmental changes, a few plants have evolved to use both the C_3_ and CAM photosynthesis pathways, with interconversion between growth stages^31^.

**Fig. 3:**
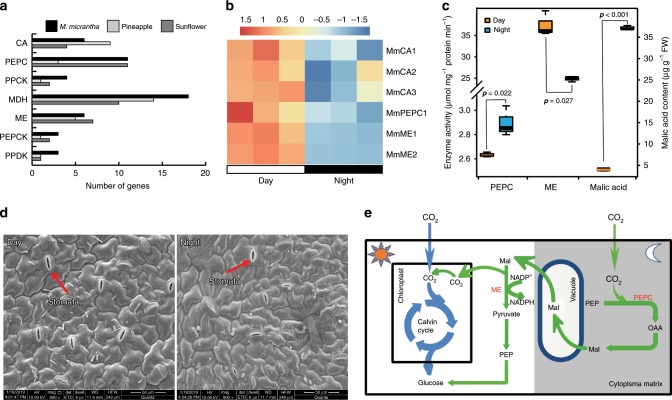
The special photosynthetic system in *Mikania micrantha.* **a** The number of putative carbon fixation genes in *M. micrantha*, pineapple and sunflower. The gene number of pineapple was obtained from the literature^28^; carbonic anhydrase (CA), phosphoenolpyruvate carboxylase (PEPC), phosphoenolpyruvate carboxykinase (PEPCK), malic enzyme (ME), malate dehydrogenase (MDH), and pyruvate orthophosphate dikinase (PPDK). **b** Expression pattern of CAM pathway key genes in *M. micrantha* across the diurnal expression data. The R-package (pheatmap)-transformed transcripts per kilobase million (TMP) expression profiles are shown. The gene names of *M. micrantha* are shown on the right. The gene expression of each replicate sample in two time points (9 A.M. and 9 P.M.) presented in the heatmap. **c** The diurnal variation pattern of key enzyme activities and malic acid content in *M. micrantha* leaves at 9:00 A.M. (day) and 9:00 P.M. (night). The *y*-axis on the left shows the activity of PEPC and NADP-linked ME. Statistical significance (*n* = 3) determined using the two-sided Student’s *t* test. On each box plot, the central mark indicates the median, the bottom and top edges of the box indicate the interquartile range (IQR) and the whiskers represent the maximum and minimum data points. **d** Scanning electron microscopy (SEM) of stomata in *M. micrantha* leaves during the day and at night. SEM images of the samples were taken using a scanning electron microscope (Q25, FEI, USA). **e** Schematic of the CAM and C_3_ photosynthesis proposed pathways of *M. micrantha*. The enzymes in the CAM pathway of *M. micrantha* are shown in red. During the day, carbon dioxide is absorbed directly by the leaves through the C_3_ pathway. At night, carbon dioxide is fixed nocturnally by PEPC and stored transiently as malic acid in large vacuoles. Furthermore, carbon dioxide is released from organic acids through NADP-linked ME and used directly in the Calvin cycle in the daytime. The source data underlying Fig. 3b–d are provided as a Source Data file.

Here, we identified genes related to the CAM pathway based on homology to Kyoto Encyclopedia of Genes and Genomes (KEGG) pathways. The *M. micrantha* genome contains 51 candidate genes involved in the carbon fixation module of CAM, including carbonic anhydrase (CA), phosphoenolpyruvate carboxylase (PEPC), phosphoenolpyruvate carboxykinase (PEPCK), malic enzyme (ME), malate dehydrogenase (MDH), and pyruvate orthophosphate dikinase (PPDK) (Fig. 3a). The gene copy number of the CAM pathway in *M. micrantha* was 1.7 and 1.4 times greater than that in pineapple (a typical CAM plant) and sunflower (a C_3_ plant), respectively. In addition, the activity of PEPC in the leaves of *M. micrantha* (2.8 μmol mg^−1^ protein) was higher than that in pineapple leaves (2.0 μmol mg^−1^ protein).

To investigate the enzyme activity patterns of the CAM pathway in day and night, we collected RNA-seq samples at 9:00 A.M. (day) and 9:00 P.M. (night) from leaf tissues of potted *M. micrantha* (see Supplementary Note 2). On the basis of contrasting expression patterns in the leaf tissues between day and night, we identified the diurnal variation of *M. micrantha* genes involved in carbon fixation (Fig. 3b). The eight key genes of CAM pathway (encoding three copies of CA, one copy of PEPC and two copies of NADP-linked ME) had a diurnal expression pattern in the leaf tissue (Fig. 3b). In addition, the enzyme activity of *M. micrantha* PEPC in the leaf tissue was higher in the night than that during the day (Fig. 3c). Carbon dioxide was fixed nocturnally by PEPC and stored transiently as malic acid in large vacuoles; consequently the malic acid and citric acid content in the evening was nearly 10 times and two times as high as that in the daytime, respectively (Fig. 3c and Supplementary Fig. 14a). The stored malic acid was decarboxylated, and the carbon dioxide was released by NADP-linked ME during the daytime; thus, the activity of NADP-linked ME showed a pattern in which the activity in the daytime was significantly higher than that at night (*p* = 0.027, two-sided Student’s *t* test, Fig. 3c).

In addition, the stomata of C_3_ and C_4_ plants open to absorb carbon dioxide during the day, while they close at night, except for in CAM plants, whose stomata exhibit a unique pattern of diurnal variation. Surprisingly, the stomata of *M. micrantha* leaves were not only always open during the day (Fig. 3d and Supplementary Fig. 14b, c), but also partly open at night (Fig. 3d and Supplementary Fig. 14b, c), implying that the CAM pathway was involved in the carbon dioxide fixation. Together, these results indicate that one of the reasons for the higher photosynthetic capacity of *M. micrantha* leaves is that CO_2_ may be absorbed at night by photosynthetic pathway similar to CAM and stored in vacuoles as organic acids to supplement the carbon fixation capacity during the day (Fig. 3e).

### High photosynthesis of the stem enhances rapid growth

**Fig. 4:**
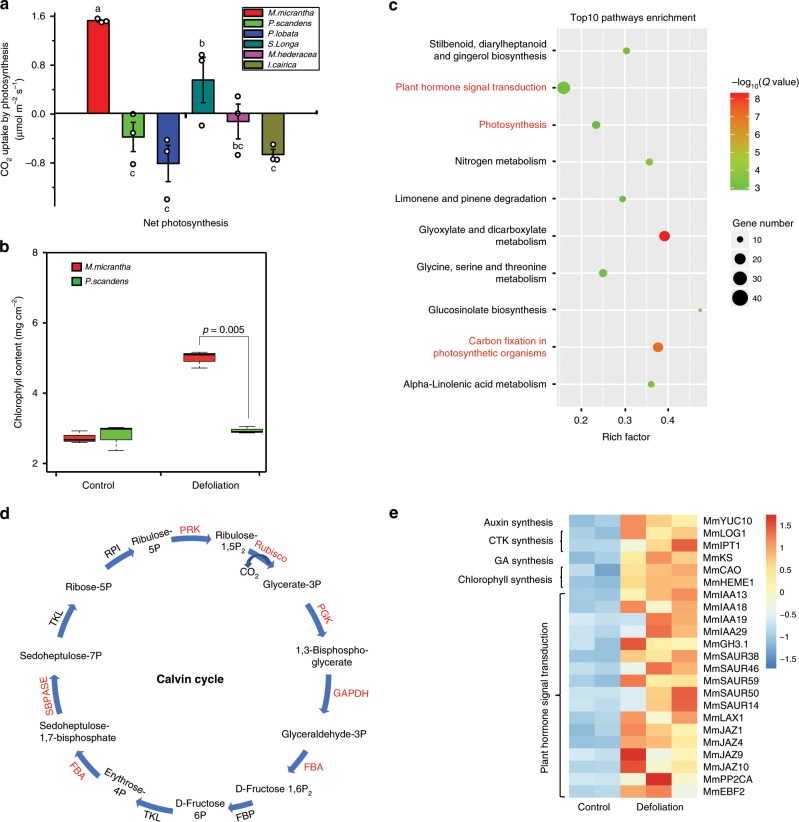
Identification of stem photosynthesis in *Mikania micrantha*. **a** Comparison of the net photosynthetic rate of *M. micrantha* to that of five neighboring species, namely, *Paederia scandens*, *Pueraria lobata*, *Stephania longa*, *Merremia hederacea*, and *Ipomoea cairica*. Statistical significance (*n* = 3) determined using one-way ANOVA with Duncan’s multiple comparison test. **b** The change in chlorophyll content in the stem under defoliation conditions compared to that in the control. Statistical significance (*n* = 3) determined using the two-sided Student’s *t* test. On each box plot, the central mark indicates the median, the bottom and top edges of the box indicate the interquartile range (IQR) and the whiskers represent the maximum and minimum data points. **c** KEGG pathway enrichment of upregulated genes. The significant enrichment of KEGG pathways related to photosynthesis, carbon fixation and hormone signal transduction are marked in red. **d** Comparison of key genes related to the Calvin cycle in the *M. micrantha* stem between defoliation conditions and the control. The significantly upregulated genes are marked in red. **e** Expression pattern of differentially expressed genes involved in auxin, cytokinin, gibberellin, and chlorophyll biosynthesis and plant hormone signal transduction. The gene names of *M. micrantha* are shown on the right. Error bars indicate average value ± s.e.m. of indicated replicates. The source data underlying Fig. 4a, b, e are provided as a Source Data file.

In most plants, the leaves are the major photosynthetic organs; however, some studies have reported that many nonleaf green organs or tissues also have photosynthetic activity^32^. To investigate the photosynthetic ability of stems in *M. micrantha*, we measured their net photosynthetic rate (Pn), respiration and gross photosynthetic rate and compared these values with those of five other species. The Pn of the *M. micrantha* stem was approximately 1.5 μmol CO_2_ m^−2^ s^−1^, and was significantly higher than that in *Paederia scandens*, *Pueraria lobata*, *Stephania longa*, *Merremia hederacea*, and *Ipomoea cairica* (*p* < 0.05, Duncan-test; Fig. 4a), which was close to or less than 0 μmol CO_2_ m^−2^ s^−1^. The gross photosynthetic rate of the *M. micrantha* stem showed a similar pattern (*p* < 0.05, Duncan-test; Supplementary Fig. 15).

To confirm that the photosynthesis of the *M. micrantha* stem could maintain plant growth, we conducted a defoliation experiment with both *M. micrantha* and its neighboring species *P. scandens* (Supplementary Note 3). All the *M. micrantha* samples grew normally without leaves for 30 days, but only 10% of the *P. scandens* samples survived (Supplementary Fig. 16). In addition, the outer epidermis of the stem of *M. micrantha* gradually changed from red to green in the defoliation treatment (Supplementary Fig. 17a). The major reason for this change was a significant reduction in the anthocyanin content (*p* = 0.001, two-sided Student’s *t* test), which was more than ten times lower than that in the stems of the undefoliated samples (Supplementary Fig. 17b), indicating that the stem of *M. micrantha* can compensate for its photosynthetic capacity by reducing anthocyanin accumulation and absorbing more external light energy. In addition, the stem of *M. micrantha* had the same photosynthetic components as the leaves, such as stomata and chlorophyll. The chlorophyll content in the stem of *M. micrantha* increased significantly in the defoliation treatment, but no significant changes occurred in *P. scandens* (Fig. 4b). Moreover, the number of grana lamellae increased, and the synthesized starch grains were also significantly larger than those in the control stems, which were similar to the patterns in the leaves (Supplementary Fig. 18). In addition, we found that the plant hormone (auxin, gibberellin, and cytokinin) contents in the *M. micrantha* stem after defoliation were significantly higher than that in the control group, and similar to that in the leaves (Supplementary Fig. 19a–c).

To investigate the gene expression patterns of the photosynthesis pathway genes in the stem of *M. micrantha*, we collected RNA-seq samples from defoliated and control stems. The upregulated genes were significantly enriched in the photosynthesis (*p* = 1.6 × 10^−4^, Deseq2), carbon fixation (*p* = 2.7 × 10^−9^, Deseq2), and plant hormone signal transduction pathways (*p* = 5.4 × 10^−5^, Deseq2; Fig. 4c). The anthocyanin contents were markedly reduced in the defoliation treatment because the expression of anthocyanin synthesis genes in this treatment were significantly downregulated compared with that in the control (Supplementary Fig. 17c, d). Six key genes in the Calvin cycle of the photosynthesis pathway showed significant upregulation in the defoliation treatment (Fig. 4d and Supplementary Fig. 20). Ribulose-1,5-bisphosphate carboxylase/oxygenase (Rubisco) is an enzyme involved in the first major step of carbon fixation and plays an important role in the process of atmospheric carbon dioxide conversion by plants. The gene expression of five Rubisco copies was four times higher than that in the control group (Supplementary Fig. 20), suggesting that the stem maintains the plant growth by regulating the expression of Rubisco in the absence of leaves. Furthermore, the chlorophyll and plant hormone signal transduction and synthesis pathways displayed similar gene expression patterns, in which the genes were significantly upregulated in the treatment group (Fig. 4e). This evidence indicated that under the defoliation stress, the *M. micrantha* gained enough energy for rapid stem growth and development by improving its photosynthetic rate and increasing its chlorophyll and hormone contents.

### Genetic pathway of *Mikania* STLs

STLs, which are mainly derived from the precursor germacrene A acid (GAA), are known for their allelopathic effects and are characteristic of the family Asteraceae^19,33,34^. Several enzymes acting downstream of GAA have been elucidated recently, such as HaG8H, LsCOS, and Ih8H, that lead to the formation of different STLs with distinctive stereochemistry^35–37^. However, the biosynthetic pathways of STLs in *Mikania* remain unknown. Lactone ring formation in STLs is dependent upon the (C6 or C8) position and stereoselective hydroxylations of GAA, which can result in STLs with four unique configurations (12, 6α-, 12, 6β-, 12, 8α-, and 12, 8β-olides derivatives of GAA)^37^. Ih8H homolog is important enzyme convert to 7, 8-trans lactone from GAA. As all the detected STLs in this study had a 7, 8-trans lactone, we were investigating for a homolog of Ih8H, an enzyme capable of synthesizing 7, 8-trans inunolide, the putative precursor of these *M. micrantha* STLs.

**Fig. 5:**
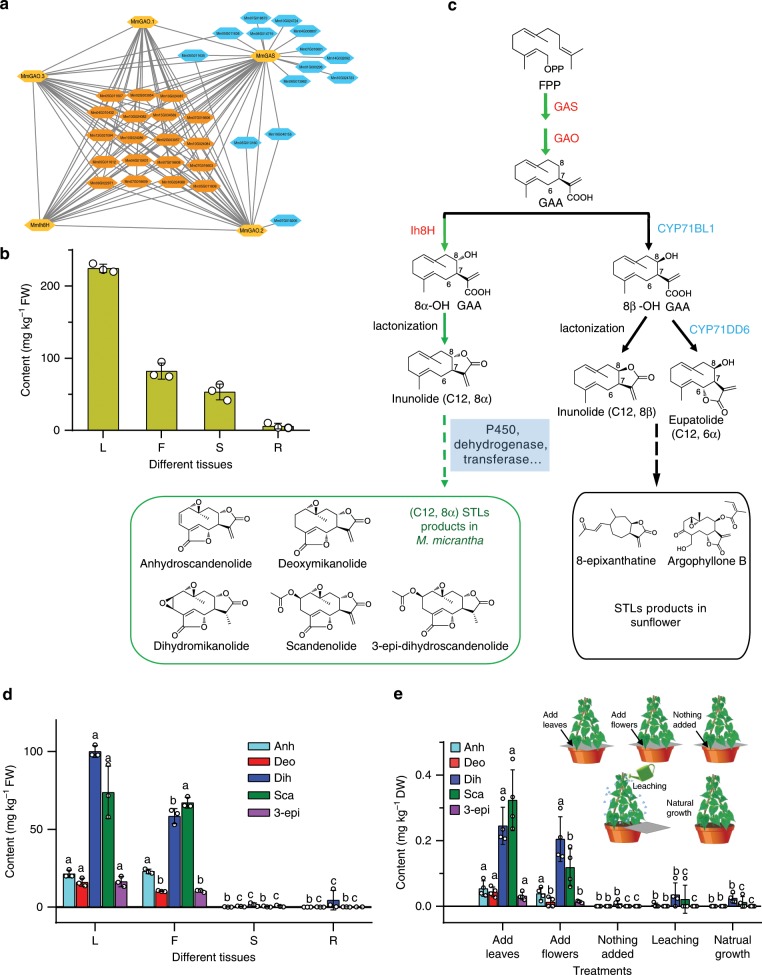
Investigation of sesquiterpene lactones and proposed biosynthetic pathway. **a** Coexpression network of the sesquiterpene lactones (STLs) biosynthesis pathway elements with a correlation coefficient (>0.7). The genes (GAO germacrene A oxidase, GAS germacrene A synthase, Ih8H germacrene A acid 8-hydroxylae), that were reported in previous studies, are colored in orange. The candidate genes involved in STLs biosynthesis from inunolide are colored in red. **b** The relative inunolide content in different fresh plant tissues (*n* = 3). L, F, S, and R represent the leaf, flower, stem, and root, respectively. **c** The hypothesized biosynthesis pathway of STLs from germacrene A acid. Green arrows indicate the proposed metabolic pathways of *Mikania micrantha* STLs. The candidate genes of the biosynthesis pathway of *M. micrantha* STLs are indicated by blue boxes. The black arrows indicate the biosynthetic pathway of STLs in sunflower from a previous report^36^. FPP farnesyl pyrophosphate, GAA germacrene A acid. **d** The amount of five STLs in fresh tissues (*n* = 3). Anh anhydroscandenolide, Deo deoxymikanolide, Dih dihydromikanolide, Sca scandenolide, 3-epi 3-epi-dihydroscandenolide. **e** Pot experiments to simulate the release of *M. micrantha* STLs into soil. Five treatments: Add leaves, dried *M. micrantha* leaves were added to the soil surface in each pot to measure STLs in the soil, mainly coming from the fallen leaves. Add flowers, dried flowers were added to the soil surface in each pot to measure STLs mainly from fallen flowers. Nothing added, nothing was added to determine STLs in the soil from root exudates. Leaching, the plants were watered to wet the growing leaves and thus determine the STLs that were mainly derived from leaching. Natural growth, this was used as the control experiment. *n* = 4 biologically independent samples. **d**, **e** Duncan-test (*p* < 0.05) was used to determine statistical significance. Different letters above the error bars indicate significant differences among means for the different parts of the plant or treatments. Error bars indicate average value ± s.d. of indicated replicates. The source data underlying Fig. 5b, d, e are provided as a Source Data file.

We reconstructed a genome-scale co-expression network for *M. micrantha* and focused on the metabolic pathways involved in inunolide synthesis, yielding a total of 38 genes in this pathway (Fig. 5a). Five genes previously reported to be involved in GAA synthesis, namely, germacrene A oxidases (GAOs), germacrene A synthase (GAS), and GAA 8-hydroxylase (Ih8H), which have high identity with the genes in *H. annuus*, *A. annua*, *L. sativa*, and *Inula hupehensis* (Supplementary Table 8 and Supplementary Fig. 21), were identified in the inunolide synthesis pathway and showed the high expression in the leaf and flower (Supplementary Fig. 22). Inunolide is a key intermediate of STL products, which can be identified and detected in fresh leaves by ultra-performance liquid chromatography (UPLC)-quadrupole time-of-flight-mass spectrometry (MS) (Supplementary Fig. 23). In addition, we have developed a liquid chromatograph MS (LC–MS) method to determine the content of inunolide (Fig. 5b), which displayed the same pattern as the expression of the five genes in different tissues (Supplementary Fig. 22). This suggests that inunolide is likely as intermediate to the STLs metabolites in *Mikania*. Thus, the proposed pathway of STLs in *M. micrantha* was displayed in green arrows (Fig. 5c). According to the co-expression network, the candidate genes of STLs biosynthesis were detected, which may regulate the pathway producing STLs in *M. micrantha* (Fig. 5a and Supplementary Table 9). Based on the previous research^38^, the candidate genes of STLs biosynthesis were assigned to CYP71 subfamily of P450 enzyme. Quantitative analysis of five available STLs (anhydroscandenolide, deoxymikanolide, dihydromikanolide, scandenolide, and 3-epi-dihydroscandenolide) showed a significant higher STLs content in the leaf and flower (*p* < 0.05, Duncan-test), but almost no STLs were detected in stem and root (Fig. 5d and Supplementary Fig. 27a). Microscopic studies revealed the high density of glandular trichomes occurred in flowers (petal) followed by leaves, less glandular trichomes were observed in stem surface (Supplementary Fig. 24). Most of STLs were extracted from glandular trichomes with the chloroform dipping extraction method (Supplementary Fig. 25). The content of five STLs in flowers and leaves trichomes present a higher abundance, but less in the stem trichomes (Supplementary Fig. 26), which further proved the STLs were derived from *Mikania* trichomes. These results are consistent with those of gene expression, which supported the proposed pathway of STLs in *M. micrantha*.

Five STLs, including the dominant constituent dihydromikanolide and scandenolide, were identified in invaded soils collected from different regions (Supplementary Fig. 27b). The STLs content in soil differed among the collection regions, which may have been related to community age, i.e., invasion history, or other ecological factors in the field. To better understand how the STLs are released into surrounding soil, a pot experiment was conducted to determine where the STLs in soil come from (litter fall from the aerial part, root exudates or leaching) (Fig. 5e and Supplementary Note 4). Five STLs were also detected in the soil after 15 days of treatment, and the STL levels in the treatments with added *M. micrantha* leaves and added flowers were higher than those in the treatments with root exudates (nothing added) and leaching (Fig. 5e). Five STLs that were almost undetectable in the root by LC–MS analysis accumulated in the leaves and flowers, which suggested that these five STLs were released into the environment by litter fall and rain, not by root exudates. In addition, the five STLs were detected in soil invaded by *M. micrantha*, providing reliable evidence that STLs are typical allelochemicals of the “mile-a-minute” weed. The proposed STLs pathway based on metabolic and genetic data will promote further investigation of the function of allelochemicals in *M. micrantha*.

### Reinforced microbial function promotes nutrient utilization

**Fig. 6:**
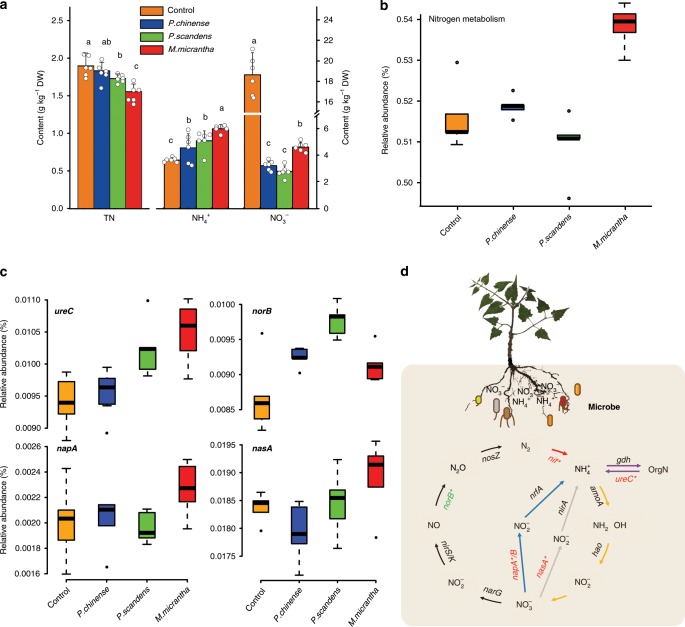
Effect of *Mikania micrantha* metabolites on soil nitrogen cycling. STLs: sesquiterpene lactones. **a** Soil nitrogen content in the pot experiment of *M. micrantha* and its two neighboring native species. *n* = 6 biologically independent soil samples. We used Duncan’s multiple range test (*p* < 0.05) method. Data with different letters in the same column are significantly different. Control indicates that no plants were planted. Error bars indicate average value ± s.d. of indicated replicates. **b** Box plots of the relative abundance of genes involved in nitrogen metabolism pathway. Adjusted p value was calculated using Dunn’s test. *n* = 5 biologically independent soil samples. **c** The relative abundance of key genes of soil microorganisms involved in assimilatory nitrogen reduction (*nasA*), dissimilatory nitrogen reduction (*napA*), ammonification (*ureC*), and denitrification (*norB*) between *M. micrantha* and its two neighboring species (*Polygonum chinense* and *Paederia scandens*) as well as in the blank control. Adjusted *p* value was calculated using Dunn’s test. *n* = 5 biologically independent soil samples. On each box plot, the central mark indicates the median, the bottom and top edges of the box indicate the interquartile range (IQR) and the whiskers represent the maximum and minimum data points. **d** A schematic of nitrogen cycling in the *M. micrantha* soil. Red and green indicate that the abundance of the gene in *M. micrantha* soil was upregulated and downregulated compared to that in the soil of the two neighboring species (*P. chinense* and *P. scandens*), respectively. Black, orange, red, blue, gray, and purple arrows indicate the denitrification, nitrification, nitrogen fixation, dissimilatory nitrogen reduction, assimilatory nitrogen reduction, and ammonification, respectively. The source data underlying Fig. 6a–c are provided as a Source Data file.

To investigate the potential effects of *M. micrantha* on soil nutrient cycling, we conducted a pot experiment with *M. micrantha* and its two neighboring native species, namely, *Polygonum chinense* and *Paederia scandens* (see Supplementary Note 5). The total nitrogen in *M. micrantha* soil significantly decreased, compared to that in the soil of its two neighboring species and the control which no plants were planted (Fig. 6a). Conversely, the available nitrogen, particularly ammonium nitrogen, in *M. micrantha* soil increased significantly more than that in the soil of its two neighboring native species (Fig. 6a and Supplementary Table 10). In addition, the variation in phosphorus and potassium content in soil showed a similar trend (Supplementary Table 10). Furthermore, we determined the contents of nitrogen, phosphorus and potassium contents in different plant tissues. The accumulation of the three elements in the different tissues of *M. micrantha* was significantly higher than that in its two neighbors (*P. chinense* and *P. scandens*) (Supplementary Fig. 28).

Soil metagenomics provides a cultivation-independent assessment of the largely untapped genetic reservoir of soil microbial communities, which has already led to the identification of functional biomolecules^39^. To investigate the effects of whole soil microbial communities on the growth of *M. micrantha* and its neighboring species, we collected soil DNA samples for metagenomic research. With all the soil samples, a total of 25,597,567 nonredundant genes were recovered, with an average open reading frame length of 537 bp. The rarefaction analysis of all soil samples showed a curve approaching saturation (Supplementary Fig. 29), suggesting that the vast majority of microbial genes in the soil where the three species grew were present in our gene catalog. Genes related to carbohydrate metabolism, energy and nitrogen metabolism, membrane transport, replication, and repair and cell motility in *M. micrantha* root microbiome was significantly enriched compared to that in its two neighbors and the control (*p* < 0.05, Duncan-test; Fig. 6b and Supplementary Figs. 30 and 31), indicating that the root microbiome of *M. micrantha* was more active than that of the two neighboring species.

In natural soils, the vast majority of N, P, and K atoms are minimally bioavailable for plants growth, but can be metabolized by various soil microbes, which means that the mineralization processes mediated by soil microbes promoted nutrient cycling in natural ecosystems^40^. In this study, the relative abundance of key genes involved in nitrogen fixation (*nif*), assimilatory nitrogen reduction (*nirB* and *nasA*), dissimilatory nitrogen reduction (*napA* and nirB/D), and ammonification (*ureC*) in *M. micrantha* soil was higher than that in the soil of its two neighboring native species (*P. chinense* and *P. scandens*) soil and the control (Fig. 6c, d and Supplementary Fig. 32), indicating that as a result of the soil microbe functioning, more available forms of nitrogen were reduced to ammonia nitrogen and ammonium was produced and accumulated so as to speed up the nitrogen cycle in *M. micrantha* soil. In contrast, the relative abundance of key genes involved in denitrification (*norB*) in *M. micrantha* soil was significantly lower than that in *P. chinense* and *P. scandens* soil (Fig. 6c, d), suggesting that there were fewer denitrifying bacteria which use nitrates in *M. micrantha* soil to carry out respiration so as to decrease the loss of available nitrate nitrogen. In addition, the population density of cultured Azotobacter, ammoniated bacteria, and phosphorus- and potassium-solubilizing bacteria (Supplementary Fig. 33) in *M. micrantha* soil were significantly higher than that in the soil of its two neighboring species soil.

Plant metabolites play an important role in plant survival in a given environment and establish ecological relationships between plants and other organisms. To investigate the metabolites effects of *M. micrantha* on soil nutrient cycling, we added five STLs (anhydroscandenolide, deoxymikanolide, dihydromikanolide, scandenolide, and 3-epi-dihydroscandenolide) separately to the bare soil near an *M. micrantha* monoculture to investigate the metabolite-microbe interactions. The STLs significantly increased not only the concentration of carbon dioxide (Supplementary Fig. 34), but also the relative abundance of nitrogen fixation bacteria (Supplementary Fig. 35), indicating that the activity of microorganisms was improved. Furthermore, the available nutrients, e.g., ammonium nitrogen, were mineralized efficiently in the soil in five STLs treatments, which was significantly higher than that in the control (Supplementary Table 11). These evidences indicated that the STLs of soil may play an important role in enriching microorganisms, which in turn accelerating the nutrient cycling (Fig. 6d). On the other hand, root zone is the primary site for interactions between microbes and plants. They provide a very attractive, nutrient-rich niche that harbors large numbers of active microbes that are important for plant health and nutrient uptake^41–43^.

## Discussion

Given the common name “mile-a-minute”, one of the most distinctive features of *M. micrantha* is its fast growth, which strongly relies on its photosynthesis ability^1^. In this study, we found that the variation pattern of malic acid content, citric acid content, stomatal conductance and key genes expression, such as PEPC, CA, NADP-ME, are consistent with classic CAM plants. This result indicates that CO_2_ may be absorbed at night by photosynthesis pathways which was similar to CAM and stored in vacuoles as organic acids to supplement the carbon fixation capacity during the day, helping to absorb more carbon dioxide at night in *M. micrantha*. The CAM pathway is mostly found in dry-desert plants and is rare in normal plants^28,44,45^, which was contrast with tropical plant *M. micrantha*. In addition, the *M. micrantha* leaves are not succulent, which was a morphological prerequisite for vacuolar storage capacity of organic acids^46^. These evidences suggested that *M. micrantha* may develop a special mechanism allowing it to achieve a higher photosynthetic rate that enables fast growth, and the details of the photosynthesis mechanism needs further research. Moreover, the stem of *M. micrantha* also has a high photosynthetic capacity, further enhancing the growth ability of stems. Thus, the special photosynthesis pathways, together with the greater photosynthetic ability of the stem, is thought to be one of the most important factors contributing to the fast growth of this species. Previous studies reported that photosynthetic efficiency could increase by modification of the photosynthetic pathway, such as introducing an intact maize gene for one of the key enzymes in C_4_ photosynthesis into rice^47^ and the alternative photorespiratory pathways^48^, implying a photosynthetic pathway by similar CAM at night in C_3_ crop could be used to improve their carbon fixation capacity through a modified photosynthetic pathway in the future.

The well-known allelochemicals STLs^21,35,36^ have a certain allelopathic effect on associated plants and soil microbes^49–51^. We found that STLs mainly accumulated in the leaves and flowers and were then released into the surrounding soil by litter fall. The STLs in the soil increased the ammonium nitrogen content by regulating microbial activity. Our results showed that the *M. micrantha* STLs could not only promote nitrogen fixation, assimilatory nitrogen reduction, dissimilatory nitrate reduction to ammonium, and ammonification, but also inhibited denitrification in the soil, indicating that the soil microbes of *M. micrantha* promoted nitrogen mineralization and decreased the loss of available nitrogen, thereby accelerating nitrogen cycling, which allowed *M. micrantha* to utilize more nutrients as well as grow rapidly. In addition, we also proposed a putative biosynthesis pathway of STLs, which may be valuable for producing biological herbicides and pharmacological chemicals derived from STLs, benefiting environmental biosecurity and human health.

## Methods

### Plant materials and sequencing

Genomic DNA was extracted from a *M. micrantha* sample, which was collected form Neilingding Island, Shenzhen, China, for constructing four Illumina PCR-free libraries with 350-bp insert size and sequenced on Illumina HiSeq x ten platforms. Totally, 339 and 14 SMRT cells with 20-kb insert library were constructed and sequenced on RSII and Sequel, respectively. Total RNA was extracted from four tissues (root, stem, leaf, and flower, three biological repeats) using the RNA extraction kit (Huayueyang, ZH120, China). The mRNA from all samples was purified using the Truseq RNA Sample Prep Kit (illumina), followed to be sequenced on an Illumina HiSeq 2500 sequencer.

### Genome assembly and annotation

The Illumina raw reads were filtered by trimming the adapter sequence and low-quality part by in-house software clean_adapter and clean_lowqual (https://github.com/fanagislab/assembly_2ndGeneration/tree/master/clean_illumina), resulting in a clean and high-quality reads data with average error rate <0.001. The raw PacBio reads were assembled by the software canu-1.6^52^, and contaminating heterozygous fragments were filtered by using the result of falcon/falcon-unzip^53^. We used the alternative heterozygous haplotype sequence (hapotig) assembled by falcon/falcon-unzip to align to the contigs assembled by canu and d filtered out the heterozygous contigs from canu assembled result (Supplementary Fig. 1). Then Illumina reads were aligned to the contigs by BWA-MEM^54^, and single base errors in the contigs were corrected by Pilon-v1.22^55^. For the Hi-C-based proximity-guided assembly, we removed duplications and kept reads that uniquely mapped to the reference genome. The assembly package, Lachesis was applied to do clustering, ordering and orienting^56^.

The gene models were predicted by EVidence Modeler v1.1.1^57^, embedded in a pipeline that integrating evidences from *ab initio* predictions, homology-based searches, and full-length transcriptome and RNA-seq alignments. Then, the protein-coding sequences were mapped by RNA-seq data and functionally annotated using UniProt^58^ and KEGG databases^59^. Finally, the gene models were retained if they had at least one supporting evidence. Gene functional annotation was performed by aligning the protein sequences to UniProt, eggNOG and KEGG databases using BLASTP v2.3.0+ (http://blast.ncbi.nlm.nih.gov/Blast.cgi) with E-value cutoff of 10^−5^. The pathway analysis and functional classification were conducted based on KEGG database. InterProScan^60^ was used to assign preliminary GO terms, Pfam domains and IPR domains to the gene models.

A de novo repeat library for *M. micrantha* was constructed by RepeatModeler (v1.0.4; http://www.repeatmasker.org/RepeatModeler/). TEs were identified by RepeatMasker (v4.0.6; http://www.repeatmasker.org/) using both Repbase library and the de novo library. Tandem repeats were predicted using Tandem Repeats Finder v4.07b^61^. The LTR-RTs were annotated with an in-house pipeline that uses LTR-finder^62^ and LTR-retriever^63^. The LTR insert time is estimated by LTR-retriever.

### Evolution analysis

Duplicated genomic fragments were identified by MCscanX^64^, requiring at least ten paralogous gene pairs per collinear block, and the duplicate_gene_classifier in MCscanX was implemented to classify the origins of the duplicate genes into different types. Orthologous and paralogous gene families were assigned by OrthoFinder^65^ with default parameters.

Gene families that contain only one gene for each species were selected to construct the phylogenetic tree. The protein sequences of each gene family were independently aligned by muscle v3.8.31^66^ and then concatenated into one super-sequence. The phylogenetic tree was constructed by maximum likelihood (ML) using PhyML v3.0^67^ with best-fit model (MtMAN) that was estimated by ProtTest3^68^. The Bayesian Relaxed Molecular Clock (BRMC) approach was adopted to estimate the neutral evolutionary rate and species divergence time using the program MCMCTree, implemented in PAML v4.9 package^69^. The calibration time interval (110–124 Mya) of grape was adopted from TimeTree (http://www.timetree.org). We used the the inter- and intra-syntentic gene pairs to calculate the synonymous mutation rate (*K*_*s*_) values by KaKs_Calculator 2.0 with default parameter^70^.

### Transcriptome data analysis

Transcriptome reads were filtered by in-house software clean_adapter and clean_lowqual (https://github.com/fanagislab/assembly_2ndGeneration/tree/master/clean_illumina) and then mapped to the reference genome of *M. micrantha* using TopHat v. 2.1.0^71^ with default settings. Reads count were calculated with HTSeq v. 0.9.1^72^ using BAM results from TopHat v. 2.1.0, and TPM values were then calculated for every gene in the samples. We continue to identify the differentially expressed genes by DEseq2^73^. Co-expression network analysis was performed by WGCNA package^74^ using all transcriptomes. WGCNA network construction and module detection was identified using an unsigned type of topological overlap matrix with a soft-thresholding power *β* of 28 (correlation coefficient *R*^2^ > 0.9).

### The STLs measure

Five STLs were isolated from the aerial part of *M. micrantha* by repeated chromatography. The structures of STLs were elucidated by nuclear magnetic resonance and high resolution electrospray ionization MS. UPLC coupled to MS was used as a method for quantitative analysis of five STLs (Supplementary Table 12) and the chemical constituents of different plant tissues (for details see the Supplementary Information).

### Statistical analysis

All statistical tests were performed using SPSS 11.5 software (SPSS Inc., USA). Soil chemical characteristics, nitrogen, phosphorus, potassium content of plant tissue and the population density of soil cultured bacteria were, respectively, compared using one-way ANOVA, followed by least significant difference tests at *p* < 0.05 or Duncan’s multiple range test, *p* < 0.05. All observations are independent of one another and scores in groups are normally distributed. A univariate *F*-test for each variable was used to interpret the respective effects. The equality of error variances was tested by using Levene’s test and the error variance of the dependent variable was considered to be equal across groups when *p* > 0.05. In the soil metagenomic analysis, the significant functional differences between *M. micrantha* and two neighboring species, control samples were determined by the Duncan-test.

### Reporting summary

Further information on research design is available in the Nature Research Reporting Summary linked to this article.

## Supplementary information

Supplementary Information

Peer Review File

Reporting Summary

## Data Availability

Data supporting the findings of this work are available within the paper and its Supplementary Information files. A reporting summary for this Article is available as a Supplementary Information file. The datasets generated and analyzed during the current study are available from the corresponding author upon request. The Whole Genome Shotgun project of *Mikaina micrantha* has been deposited at DDBJ/ENA/GenBank under the accession SZYD00000000 [https://www.ncbi.nlm.nih.gov/nuccore/CM018680.1/] with BioProject ID PRJNA528368. The version described in this paper is version SZYD01000000. For details, PacBio SMRT data are under accessions SRR8816384 and SRR8834228–SRR8834566; genomic Illumina DNA data are under accessions SRR8835135, SRR8835136, and SRR8835137; RNA-seq data are under accessions SRR8857616–SRR8857640; full-length transcriptome data are under accessions SRR8834701–SRR8834748. For the defoliation experiment, the RNA-seq data have been deposited as SRR8846782–SRR8846787. The metagenomics data have been deposited as SRR8936416–SRR8936475. The *M. micrantha* genome assembly, gene prediction and functional annotation datas in this paper can also be accessed at ftp://ftp.agis.org.cn/Mikania_micrantha/. The source data underlying Figs. 3b–d, 4a, b, e, 5b, d, e, 6a–c, as well as Supplementary Figs. 13–16, 17a, b, d, 18–20, 22, 26–28, 30–35, and Supplementary Tables 10 and 11 are provided as a Source Data file.

## Source data

Source Data

## Code availability

The in-house software clean_adapter [https://github.com/fanagislab/assembly_2ndGeneration/blob/master/clean_illumina/clean_adapter] and clean_lowqual [https://github.com/fanagislab/assembly_2ndGeneration/blob/master/clean_illumina/clean_lowqual] are used to filter the adapter and low-quality sequence.

## References

[1] Day M (2016). Biology and impacts of Pacific islands invasive species. 13. Mikania micrantha Kunth (Asteraceae). Pac. Sci..

[2] Lowe S., Browne M., Boudjelas S., De Poorter M. *100 of the World’s Worst Invasive Alien Species, A Selection from the Global Invasive Species Database*. (IUCN/SSC Invasive Species Specialist Group, Auckland, 2000).

[3] Zhang LY, Ye WH, Cao HL, Feng HL (2004). *Mikania micrantha* H.B.K. in China—an overview. Weed Res..

[4] Manrique V, Diaz R, P. Cuda J, Overholt W (2011). Suitability of a new plant invader as a target for Biological control in Florida. Invasive Plant Sci. Manag..

[5] Day M (2012). *Mikania micrantha* Kunth (Asteraceae) (mile-a-minute): its distribution and physical and socioeconomic impacts in Papua New Guinea. Pac. Sci..

[6] Zhong X, Huang Z, Si H, Zan Q (2004). Analysis of ecological-economic loss caused by weed *Mikania micrantha* in Neilingding Island, Shenzhen, China. J. Trop. Subtrop. Bot..

[7] Macanawai A, Day M, Tumaneng-Diete T, Adkins S (2012). Impact of *Mikania micrantha* on crop production systems in Viti Levu, Fiji. Pak. J. Weed Sci. Res..

[8] Li W-h, Zhang C-b, Jiang H-b, Xin G, Yang Z-y (2006). Changes in soil microbial community associated with invasion of the exotic weed, *Mikania micrantha* H.B.K. Plant Soil.

[9] W-h Li, C-b Zhang, G-j Gao, Q-j Zan, Z-y Yang (2007). Relationship between *Mikania micrantha* invasion and soil microbial biomass, respiration and functional diversity. Plant Soil.

[10] Kaur R, Malhotra S, Inderjit (2012). Effects of invasion of *Mikania micrantha* on germination of rice seedlings, plant richness, chemical properties and respiration of soil. Biol. Fertil. Soils.

[11] Shen S (2015). Effects of invasive plant *Mikania micrantha* on plant community and diversity in farming systems. Asian J. Plant Sci..

[12] Li M (2012). Evaluation of the controlling methods and strategies for *Mikania micrantha* H. B. K. Acta Ecol. Sin..

[13] Kuo Y, Chen T, Lin C (2002). Using a consecutive-cutting method and allelopathy to control the invasive vine, *Mikania micrantha* HBK. Taiwan J. For. Sci..

[14] Hu Y, But P (1994). A study on life cycle and response to herbicides of *Mikania micrantha*. ACTA Sci. Nat. Univ. Sunyatseni.

[15] Yang Q (2003). An investigation of the effects of environmental factors on the flowering and seed setting of *Mikania micrantha* HB K (Compositae). J. Trop. Subtrop. Bot..

[16] Xiong D (2010). Morphological and physiological plasticity responding to different light environments of the invasive plant, *Mikania micrantha* H.B.Kunth. Ecol. Environ. Sci..

[17] Chen B-M, Peng S-L, Ni G-Y (2009). Effects of the invasive plant *Mikania micrantha* H.B.K. on soil nitrogen availability through allelopathy in South China. Biol. Invasions.

[18] Wu A-P (2009). Differential belowground allelopathic effects of leaf and root of *Mikania micrantha*. Trees.

[19] Rice E. L. *Allelopathy* 2nd edn (1983).

[20] Shao H, Peng S, Wei X, Zhang D, Zhang C (2005). Potential allelochemicals from an invasive weed *Mikania micrantha* H.B.K. J. Chem. Ecol..

[21] Huang HJ, Ye WH, Wei XY, Zhang CX (2008). Allelopathic potential of sesquiterpene lactones and phenolic constituents from *Mikania micrantha* H. B. K. Biochem Syst. Ecol..

[22] Piyasena KGNP, Dharmaratne HRW (2013). Allelopathic activity studies of *Mikania scandens*. Nat. Prod. Res..

[23] Xu QL, Xie HH, Xiao HL, Wei XY (2013). Phenolic constituents from the roots of *Mikania micrantha* and their allelopathic effects. J. Agr. Food Chem..

[24] Reyes-Chin-Wo S (2017). Genome assembly with *in vitro* proximity ligation data and whole-genome triplication in lettuce. Nat. Commun..

[25] Badouin H (2017). The sunflower genome provides insights into oil metabolism, flowering and Asterid evolution. Nature.

[26] Scaglione D (2016). The genome sequence of the outbreeding globe artichoke constructed *de novo* incorporating a phase-aware low-pass sequencing strategy of F1 progeny. Sci. Rep..

[27] Peng Y (2014). *De novo* genome assembly of the economically important weed horseweed using integrated data from multiple sequencing platforms. Plant Physiol..

[28] Ming R (2015). The pineapple genome and the evolution of CAM photosynthesis. Nat. Genet..

[29] Gawronska K, Romanowska E, Miszalski Z, Niewiadomska E (2013). Limitation of C3-CAM shift in the common ice plant under high irradiance. J. Plant Physiol..

[30] Adams P (1998). Growth and development of *Mesembryanthemum crystallinum* (Aizoaceae). New Phytol..

[31] Winter K, Holtum J (2005). The effects of salinity, crassulacean acid metabolism and plant age on the carbon isotope composition of *Mesembryanthemum crystallinum* L., a halophytic C3-CAM species. Planta.

[32] Aschan G, Pfanz H (2003). Non-foliar photosynthesis—a strategy of additional carbon acquisition. Flora.

[33] Picman AK (1986). Biological activities of sesquiterpene lactones. Biochem. Syst. Ecol..

[34] Bais HP, Ramarao V, Simon G, Callaway RM, Vivanco JM (2003). Allelopathy and exotic plant invasion: from molecules and genes to species interactions. Science.

[35] Ikezawa N (2011). Lettuce costunolide synthase (CYP71BL2) and its homolog (CYP71BL1) from sunflower catalyze distinct regio- and stereoselective hydroxylations in sesquiterpene lactone metabolism. J. Biol. Chem..

[36] Frey M, Schmauder K, Pateraki I, Spring O (2018). Biosyinthesis of Eupatolide—a metabolic route for sesquiterpene lactone formation involving the P450 enzyme CYP71DD6. Acs Chem. Biol..

[37] Gou JB (2018). Discovery of a non-stereoselective cytochrome P450 catalyzing either 8 alpha- or 8 beta-hydroxylation of germacrene A acid from the Chinese medicinal plant, *Inula hupehensis*. Plant J..

[38] Nelson D, Werck-Reichhart D (2011). A P450-centric view of plant evolution. Plant J..

[39] Daniel R (2005). The metagenomics of soil. Nat. Rev. Microbiol..

[40] Rovira A. D. Plant root exudates and their influence upon soil microorganisms. (eds K. F. Baker and W. C. Snyder) 170–186. (University of California Press, Berkeley, Los Angeles, 1965).

[41] Alqueres S (2013). The bacterial superoxide dismutase and glutathione reductase are crucial for endophytic colonization of rice roots by Gluconacetobacter diazotrophicus PAL5. Mol. Plant Microbe Interact..

[42] Reinhold-Hurek B, Bunger W, Burbano CS, Sabale M, Hurek T (2015). Roots shaping their microbiome: global hotspots for microbial activity. Annu Rev. Phytopathol..

[43] Velmourougane K, Prasanna R, Saxena AK (2017). Agriculturally important microbial biofilms: present status and future prospects. J. Basic Microbiol..

[44] Osmond CB (1978). Crassulacean acid metabolism: a curiosity in context. Annu. Rev. Plant Physiol..

[45] Borland AM (2014). Engineering crassulacean acid metabolism to improve water-use efficiency. Trends Plant Sci..

[46] Cushman JC, Bohnert HJ (1999). Crassulacean acid metabolism: molecular genetics. Annu Rev. Plant Physiol. Plant Mol. Biol..

[47] Ku MS (1999). High-level expression of maize phosphoenolpyruvate carboxylase in transgenic rice plants. Nat. Biotechnol..

[48] South PF, Cavanagh AP, Liu HW, Ort DR (2019). Synthetic glycolate metabolism pathways stimulate crop growth and productivity in the field. Science.

[49] Callaway RM, Aschehoug ET (2000). Invasive plants versus their new and old neighbors: a mechanism for exotic invasion. Science.

[50] Rial C, Novaes P, Varela RM, Molinillo JM, Macias FA (2014). Phytotoxicity of cardoon (*Cynara cardunculus*) allelochemicals on standard target species and weeds. J. Agric Food Chem..

[51] Molinaro, F. et al. The effect of isabelin, a sesquiterpene lactone from *Ambrosia artemisiifolia* on soil microorganisms and human pathogens. *FEMS Microbiol. Lett.* **365**, fny001 (2018). 10.1093/femsle/fny001 29319784

[52] Berlin K (2015). Assembling large genomes with single-molecule sequencing and locality-sensitive hashing. Nat. Biotechnol..

[53] Chin CS (2016). Phased diploid genome assembly with single-molecule real-time sequencing. Nat. Methods.

[54] Li H, Durbin R (2009). Fast and accurate short read alignment with Burrows-Wheeler transform. Bioinformatics.

[55] Walker BJ (2014). Pilon: an integrated tool for comprehensive microbial variant detection and genome assembly improvement. PLoS One.

[56] Burton JN (2013). Chromosome-scale scaffolding of *de novo* genome assemblies based on chromatin interactions. Nat. Biotechnol..

[57] Haas BJ (2008). Automated eukaryotic gene structure annotation using EVidenceModeler and the Program to Assemble Spliced Alignments. Genome Biol..

[58] Wu CH (2006). The Universal Protein Resource (UniProt): an expanding universe of protein information. Nucleic Acids Res..

[59] Kanehisa M, Goto S, Kawashima S, Okuno Y, Hattori M (2004). The KEGG resource for deciphering the genome. Nucleic Acids Res..

[60] Quevillon E (2005). InterProScan: protein domains identifier. Nucleic Acids Res..

[61] Benson G (1999). Tandem repeats finder: a program to analyze DNA sequences. Nucleic Acids Res..

[62] Xu Z, Wang H (2007). LTR_FINDER: an efficient tool for the prediction of full-length LTR retrotransposons. Nucleic Acids Res..

[63] Ou S, Jiang N (2018). LTR_retriever: A highly accurate and sensitive program for identification of long terminal repeat retrotransposons. Plant Physiol..

[64] Tang H (2008). Synteny and collinearity in plant genomes. Science.

[65] Emms DM, Kelly S (2015). OrthoFinder: solving fundamental biases in whole genome comparisons dramatically improves orthogroup inference accuracy. Genome Biol..

[66] Edgar RC (2004). MUSCLE: multiple sequence alignment with high accuracy and high throughput. Nucleic Acids Res..

[67] Guindon S (2010). New algorithms and methods to estimate maximum-likelihood phylogenies: assessing the performance of PhyML 3.0. Syst. Biol..

[68] Darriba D, Taboada GL, Doallo R, Posada D (2011). ProtTest 3: fast selection of best-fit models of protein evolution. Bioinformatics.

[69] Yang Z (2007). PAML 4: phylogenetic analysis by maximum likelihood. Mol. Biol. Evol..

[70] Wang D, Zhang Y, Zhang Z, Zhu J, Yu J (2010). KaKs_Calculator 2.0: a toolkit incorporating gamma-series methods and sliding window strategies. Genomics Proteom. Bioinforma..

[71] Kim D (2013). TopHat2: accurate alignment of transcriptomes in the presence of insertions, deletions and gene fusions. Genome Biol..

[72] Anders S, Pyl PT, Huber W (2015). HTSeq—a Python framework to work with high-throughput sequencing data. Bioinformatics.

[73] Love MI, Huber W, Anders S (2014). Moderated estimation of fold change and dispersion for RNA-seq data with DESeq2. Genome Biol..

[74] Hollender CA (2014). Floral transcriptomes in woodland strawberry uncover developing receptacle and anther gene networks. Plant Physiol..

