# Supplementary material for: Mikania micrantha genome provides insights into the molecular mechanism of rapid growth
Source: Nat Commun. 2020 Jan 17;11:340. doi: 10.1038/s41467-019-13926-4 (PMC6969026; doi:10.1038/s41467-019-13926-4)
Supplement: Supplementary file 1 — Supplementary Information [file 41467_2019_13926_MOESM1_ESM.pdf]

Supplementary Information

Mikania micrantha genome provides insights into the molecular mechanism of rapid growth

Liu *et al.*

Supplementary Figures

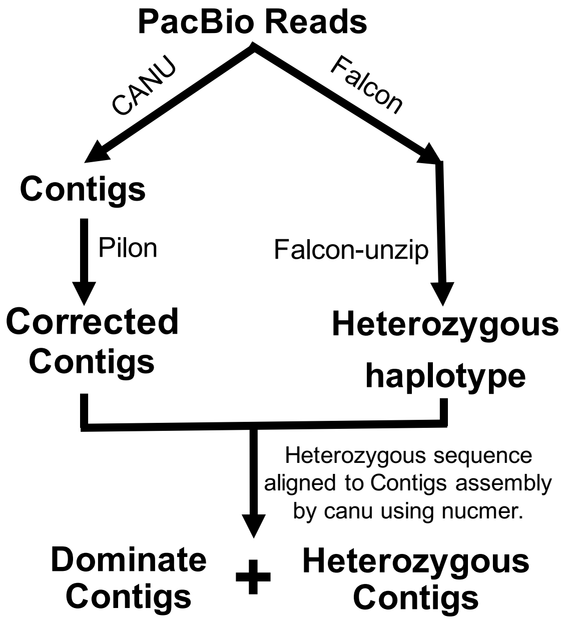

Supplementary Figure 1. The flow chart of *M. micrantha* genome assembly. We used the canu, falcon and falcon-unzip to assemble two versions of this genome, respectively. Then, the assembly sequence (by canu) as a reference genome, were aligned to it with the heterozygous sequences (by falcon and falcon-unzip). More than 70% coverage of alignment sequences were considered as heterozygous contigs. And the remain contigs were considered as the dominate contigs. In this study, we used the dominate contigs for the further analysis.

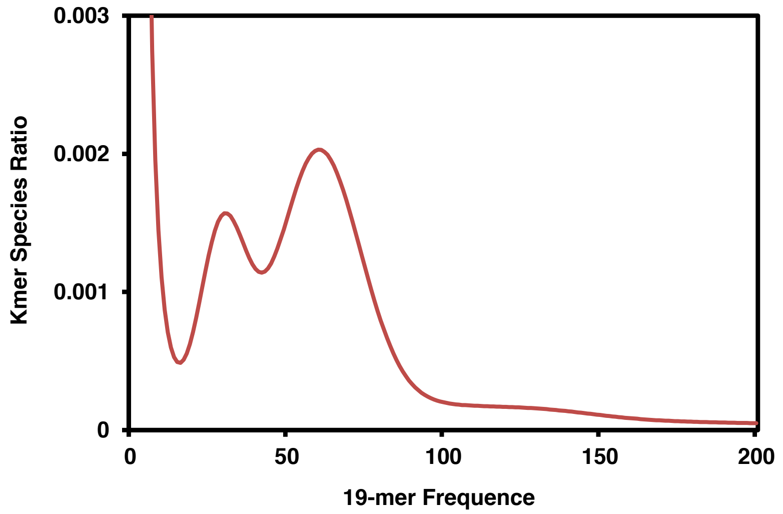

Supplementary Figure 2. Distribution of 19-mer frequency in the sequence reads. We used Illumina reads from the short insert-size libraries to calculate the kmer frequencies. And The estimated genome size of *M. micrantha* was ~1.87Gb.

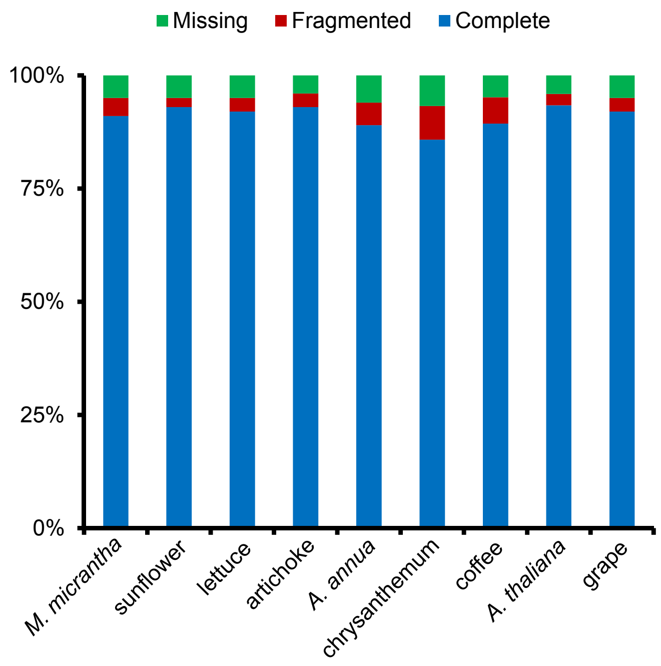

Supplementary Figure 3. Assessing genome assembly and annotation completeness with single-copy orthologs.

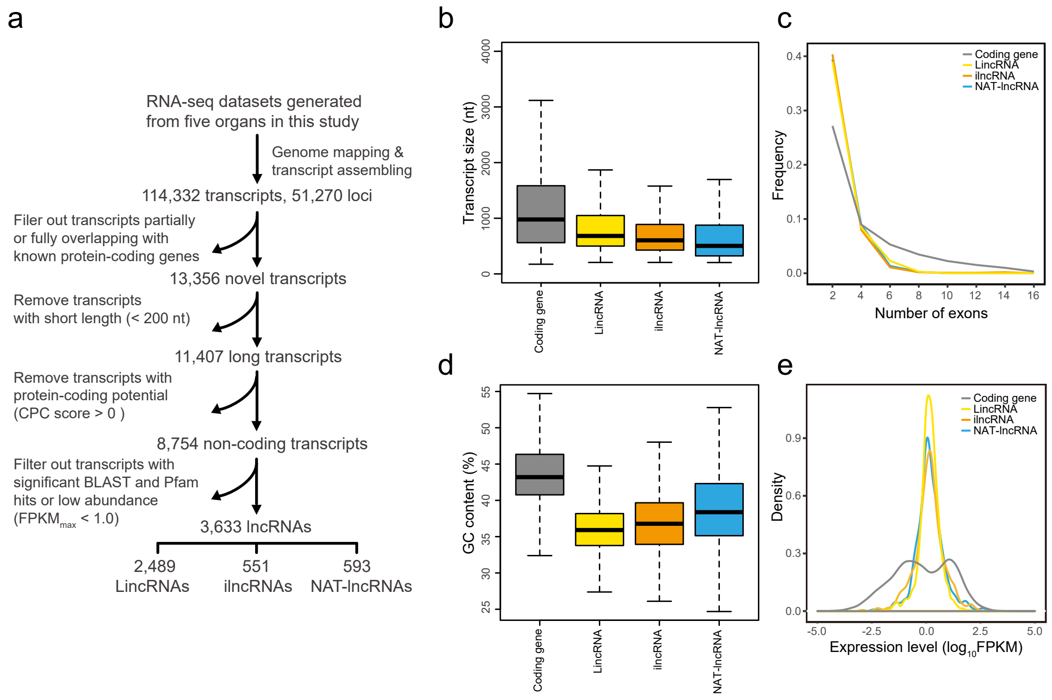

Supplementary Figure 4. Identification and characteristics of long non-coding RNAs (lncRNAs) in *M. micrantha*. a, The flowchart for bioinformatics analysis of lncRNAs in *M. micrantha*. **b,** Boxplot showing the distribution of transcript size of protein-coding genes and three subclasses of lncRNAs. On each box plot, the central mark indicates the median, the bottom and top edges of the box indicate the interquartile range (IQR) and the whiskers represent the maximum and minimum data points. **c,** The frequency distribution pattern of the number of exons among protein-coding genes and lncRNAs. **d,** The boxplot showing GC content of protein-coding genes and lncRNAs. On each box plot, the central mark indicates the median, the bottom and top edges of the box indicate the interquartile range (IQR) and the whiskers represent the maximum and minimum data points. **e,** The density plot showing the distribution of $\log_{10}$ normalized expression levels of protein-coding genes and lncRNAs.

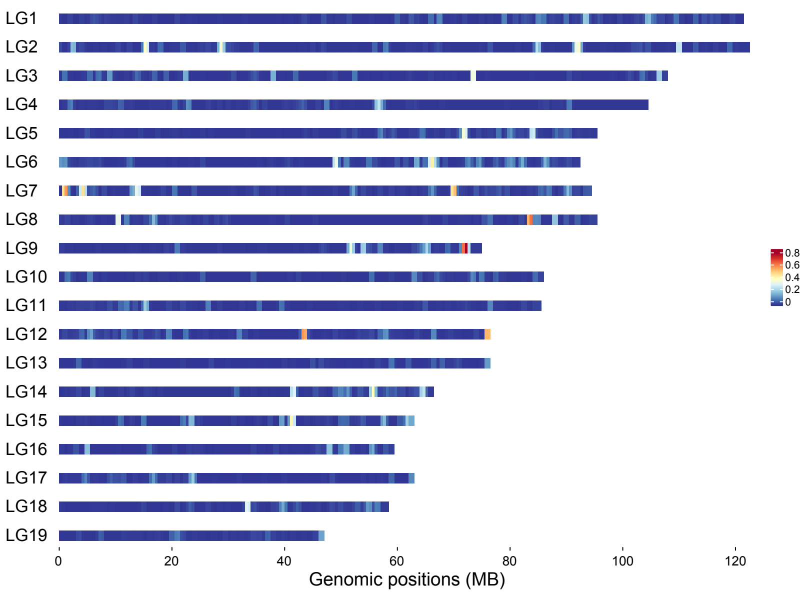

Supplementary Figure 5. Genome distribution of long non-coding RNAs (lncRNAs) in *M. micrantha*. We used the 50Kb window size to calculate the density of lncRNAs.

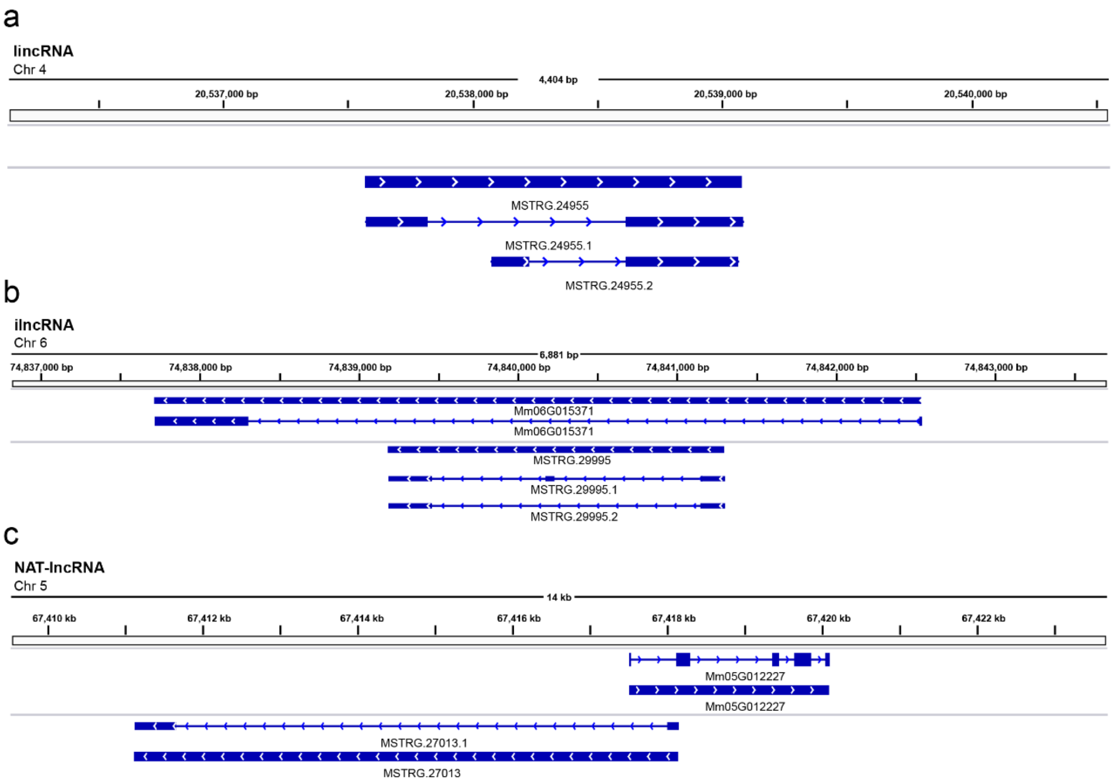

Supplementary Figure 6. Classification of long non-coding RNAs (lncRNAs) in *M. micrantha*.

a, A representative gene locus of long intergenic ncRNA. **b,** An example of intronic lncRNA. **c,** Illustrated is an example of natural antisense lncRNA. The "Mm06G015371" and "Mm05G012227" were the gene location.

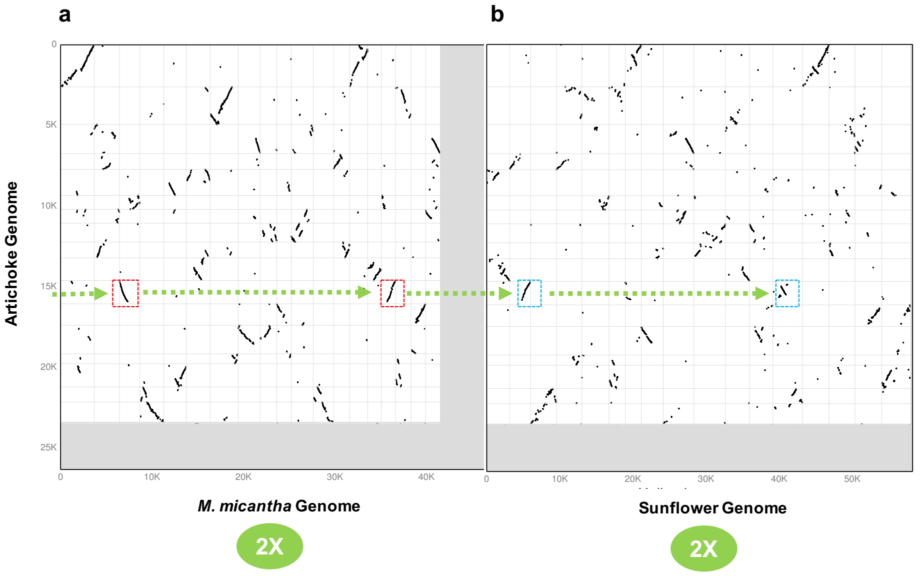

Supplementary Figure 7. Dot plots of syntenic orthologous between artichoke and sunflower, *M. micrantha*. The diploidization events are showed in red and blue boxes, respectively.

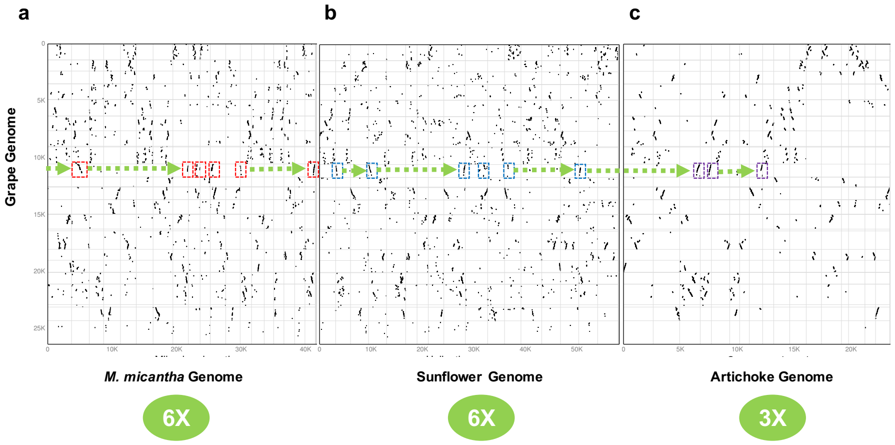

Supplementary Figure 8. Dot plots of syntenic orthologous between grape and artichoke, sunflower and *M. micrantha*. The polyploidization events are showed in red, blue and purple boxes, respectively.

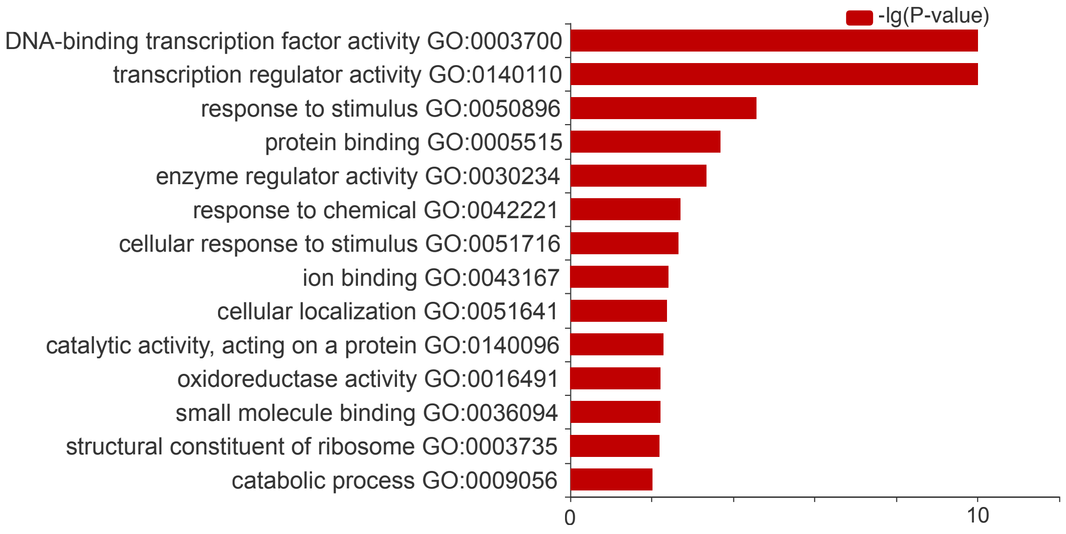

Supplementary Figure 9. The top functional enrichment of duplicated genes in *M. micrantha* by Gene Ontology.

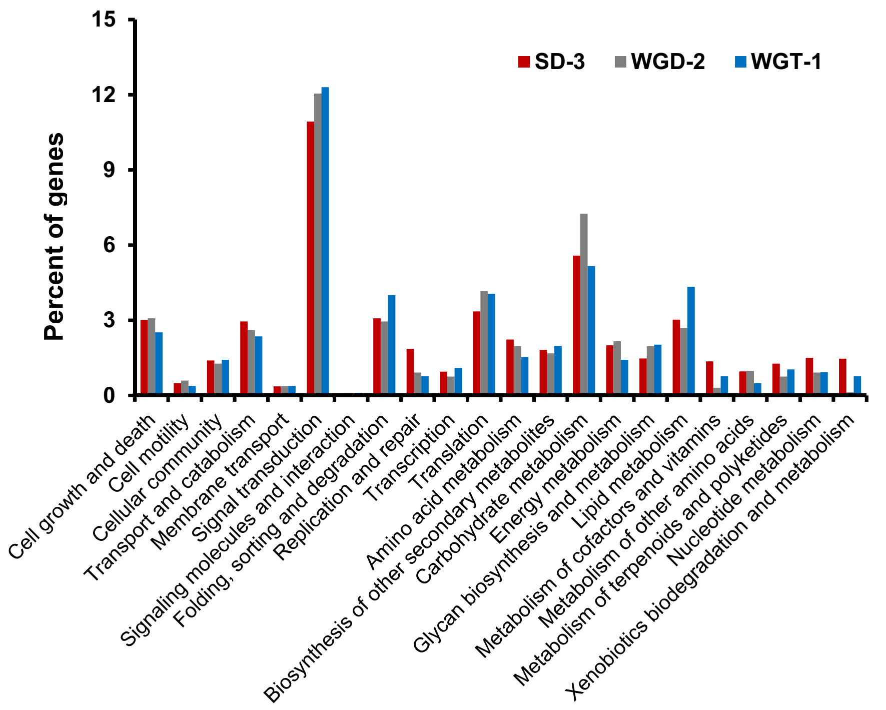

Supplementary Figure 10. The functional enrichment of WGT-1, WGD-2 and SD-3 duplicated genes in *M. micrantha* by KEGG pathways.

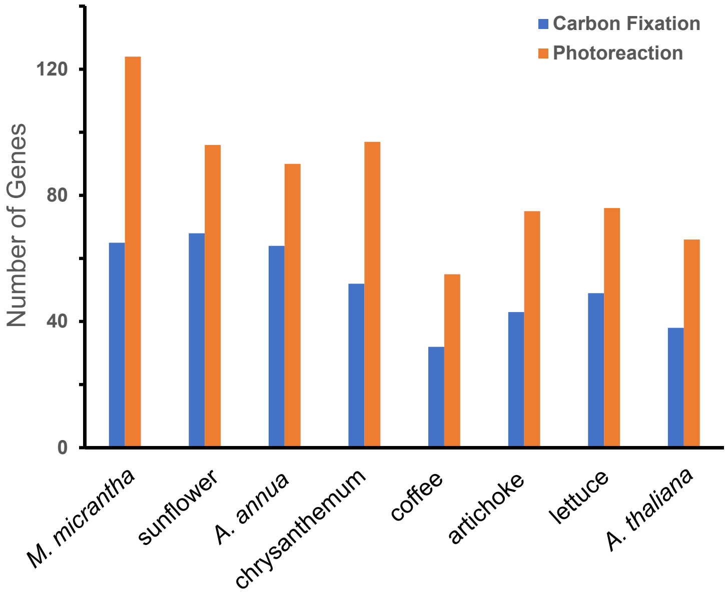

Supplementary Figure 11. The number of photoreaction and carbon fixation genes in Asterids species and *A. thaliana*.

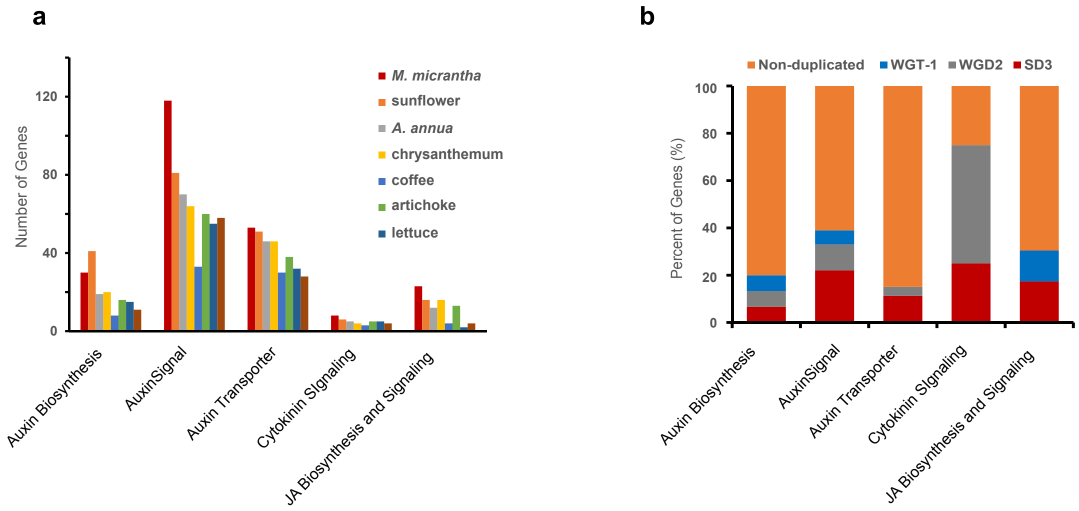

Supplementary Figure 12. The comparison of hormone biosynthesis, transport and signaling genes in Asterids species.

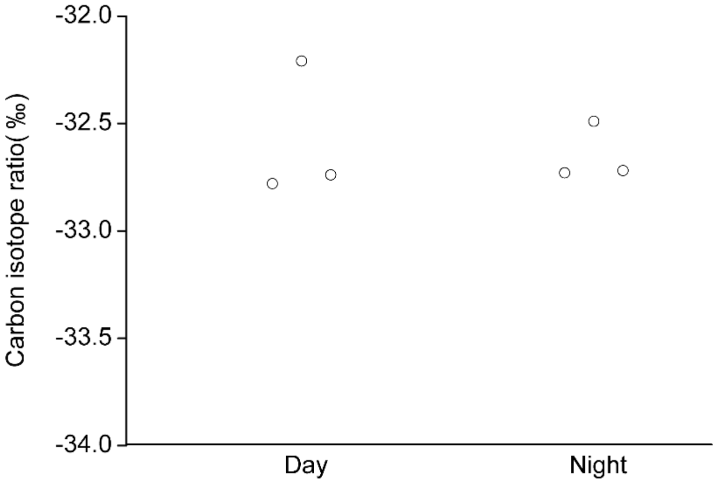

Supplementary Figure 13. Diurnal variation of carbon isotope ratio in *M. micrantha* leaves. Statistical significance (n = 3) determined using the two-sided Student' s t test. Error bars indicate mean ± s.e.m. of indicated replicates. The source data is provided as a Source Data file.

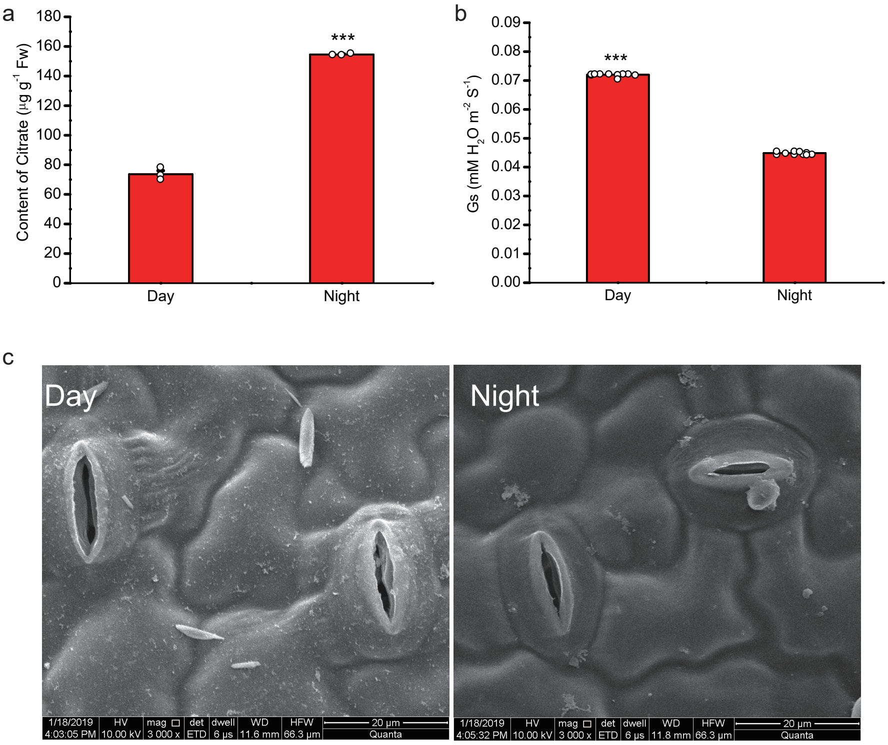

Supplementary Figure 14. The stomatal scanning electron microscopy and conductance diurnal variation in *M. micrantha* leaves. a-b, Diurnal variation of citric acid contents (**a**) and stomatal conductance (**b**) in *M. micrantha* leaves. Statistical significance (n = 3 or 10) determined using the two-sided Student' s t test. Error bars indicate mean ± s.e.m. of indicated replicates. ***, adjusted P value < 0.001 comparing Day (9 a.m) vs Night (9 p.m); **c**, Scanning electron microscopy (SEM, Q25, FEI, USA) of stomata during the day and night. The source data is provided as a Source Data file.

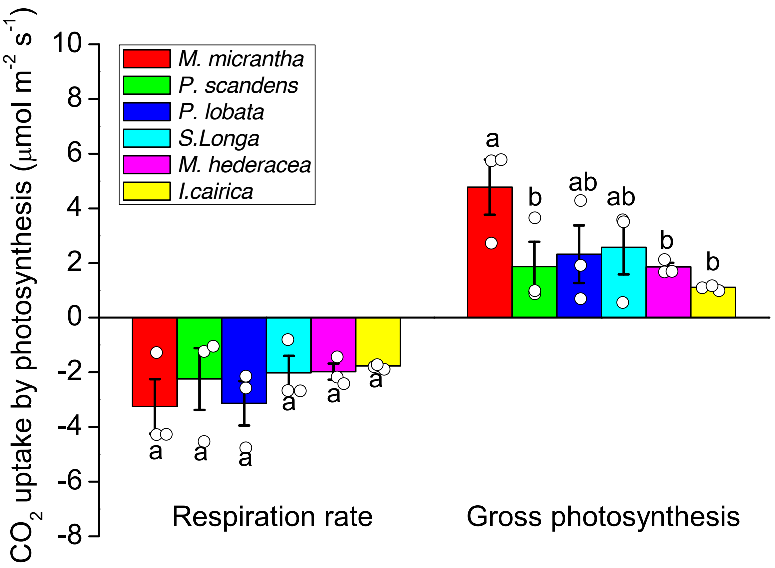

Supplementary Figure 15. The respiration and gross photosynthetic rate in *M. micrantha* stem compared with other five species. Statistical significance (n = 3) determined using one-way ANOVA with Duncan's multiple comparison test. Error bars indicate mean ± s.e.m. of indicated replicates. The source data is provided as a Source Data file.

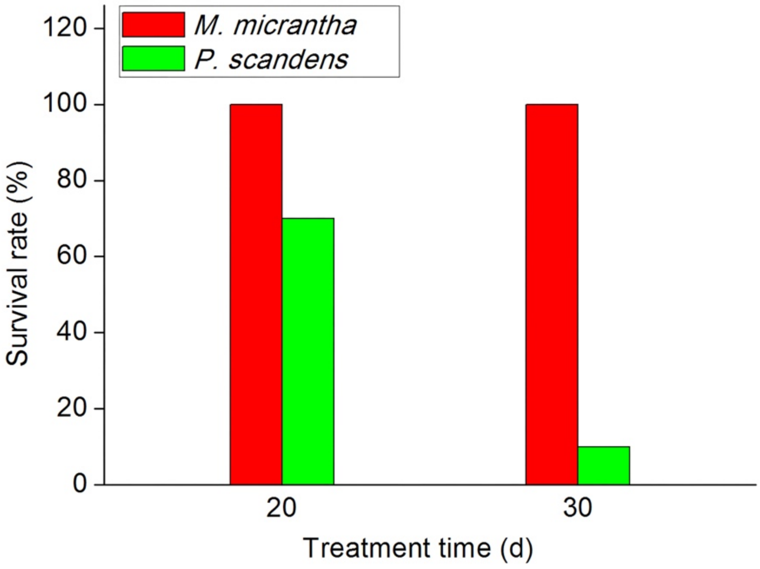

Supplementary Figure 16. The survival rates of the *M. micrantha* and *P. scandens* under the defoliation experiment.

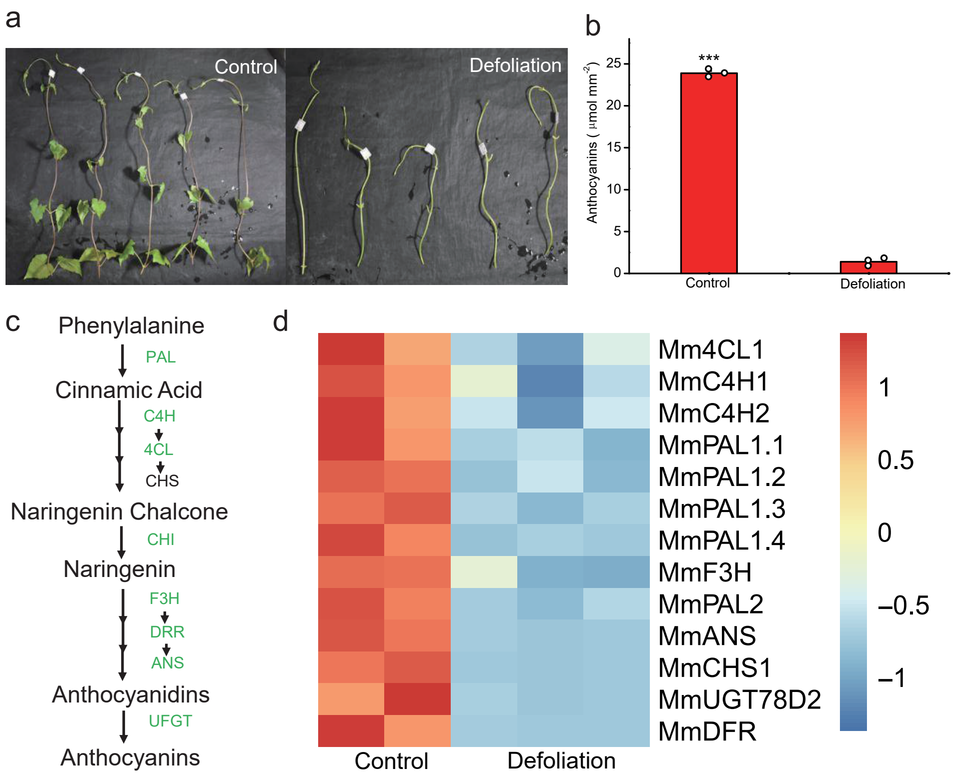

Supplementary Figure 17. The inhibition of anthocyanin synthesis under defoliation treatment in *M. micrantha*. a, The phenotypic changes of *M. micrantha* stem. (n=5); **b**, The anthocyanin contents of stem under the defoliation treatment. Statistical significance (n = 3) determined using the two-sided Student' s t test. Error bars indicate mean ± s.e.m. of indicated replicates. ***, adjusted P value < 0.001. **c**, The anthocyanin synthesis pathway. The down-regulated genes are marked in green. **d**, Heatmap of differentially expressed genes of anthocyanin synthesis genes under the defoliation treatment. The source data is provided as a Source Data file.

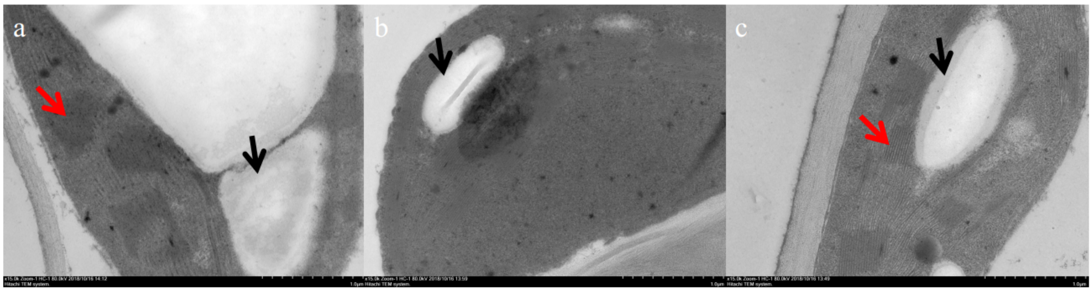

Supplementary Figure 18. Distribution and composition of chloroplasts on the surface of *M. micrantha* stem and leaf. a, Chloroplasts in the control leaf. **b**, Chloroplasts in the control stem. **c**, Chloroplasts in *M. micrantha* stem under the defoliation treatment. All the photographs have same magnification of 15,000X. The red arrow represents the grana lamella; The black arrow represents the starch grains. The source data is provided as a Source Data file.

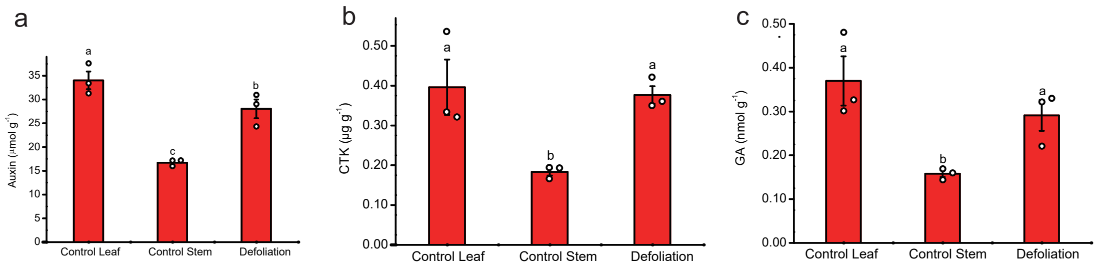

Supplementary Figure 19. The plant hormone (a, auxin; b, cytokinin; c, gibberellin) contents in *M. micrantha* stem under the defoliation treatment. Statistical significance (n = 3) determined using one-way ANOVA with Duncan's multiple comparison test. Error bars indicate mean ± s.e.m. of indicated replicates. The source data is provided as a Source Data file.

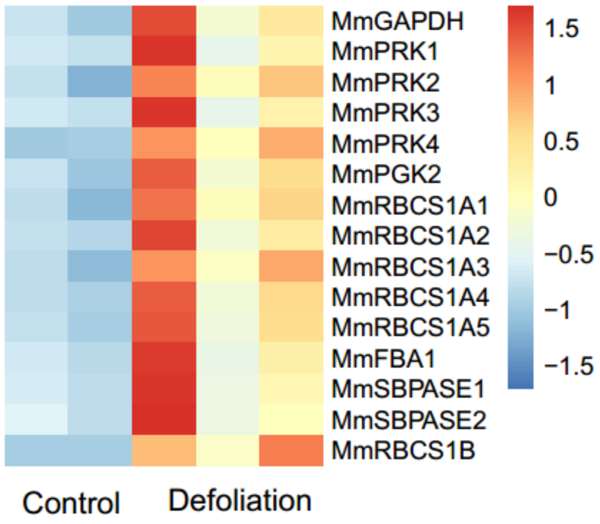

Supplementary Figure 20. The gene expression pattern of Calvin cycle in *M. micrantha* stem under the control and defoliation experiment. The source data is provided as a Source Data file.

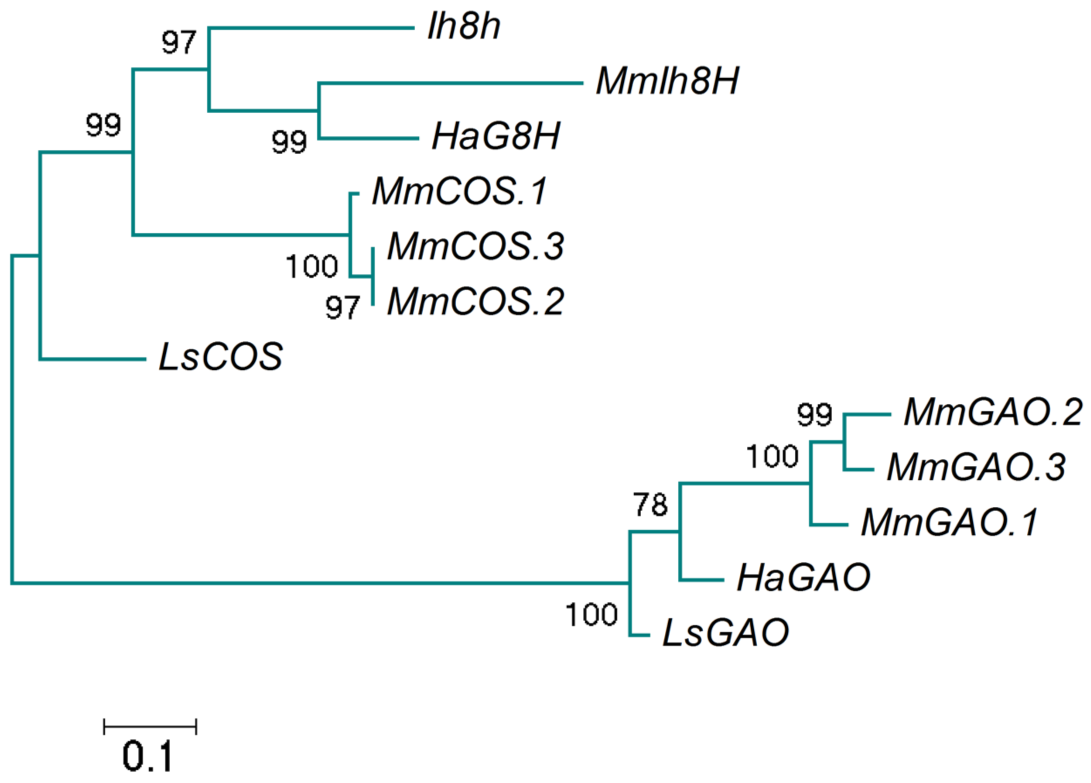

Supplementary Figure 21. The maximum likelihood phylogenetic tree of STL biosynthesis pathway key genes in *Mikania micrantha, Helianthus annuus*, *Artemisia annua*, *Lactuca sativa* and *Inula hupehensis*.

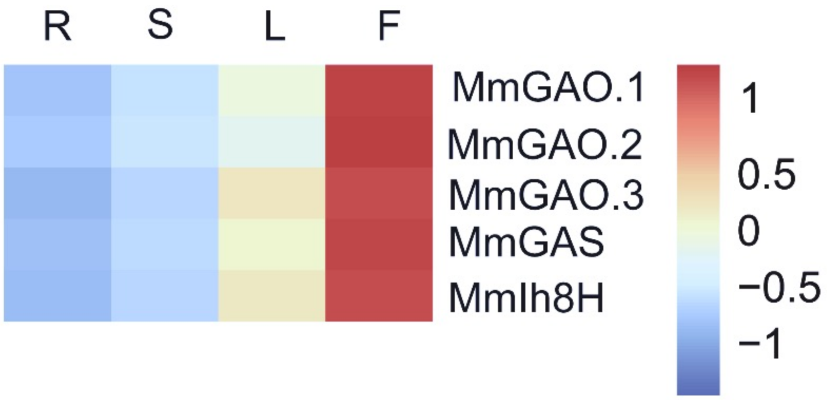

Supplementary Figure 22. The gene expression of GAA synthesis, including three germacrene A oxidase (GAO), one germacrene A synthase (GAS) and one germacrene A acid 8-hydroxylas (Ih8H). R: root; S: stem; L: leaf; and F: flower. Three copies of GAO in *M. micrantha* genome were named MmGAO.1, MmGAO.2 and MmGAO.3, respectively. The source data is provided as a Source Data file.

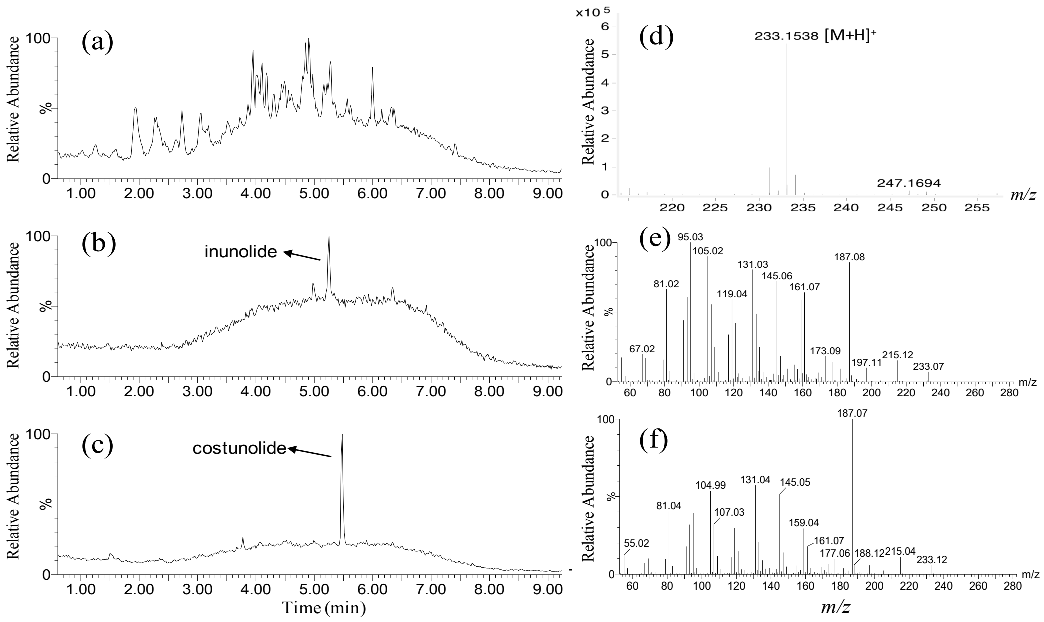

Supplementary Figure S23. LC-MS analyzed dichloromethane dipped extract of *M. micrantha* leaves, inunolide and costunolide standard in parallel (positive mode). (a) Total ion chromatograph (TIC) of chloroform dipped extract of leaves; (b) TIC of innunolide (Rt 5.25 min) isolated from *M. micrantha*; (c) TIC of costunolide standard (Rt 5.48 min); (d) The high resolution mass spectrum of inunolide in positive ion, the molecular formula $C_{15}H_{20}O_2$ from its molecular ions at m/z 233.1538$[M+H]^+$ (calcd for $C_{15}H_{21}O_2$, 233.1542); (e)-(f) MSMS spectrum of inunolide and costunolide (collision energy was set at 20 eV).

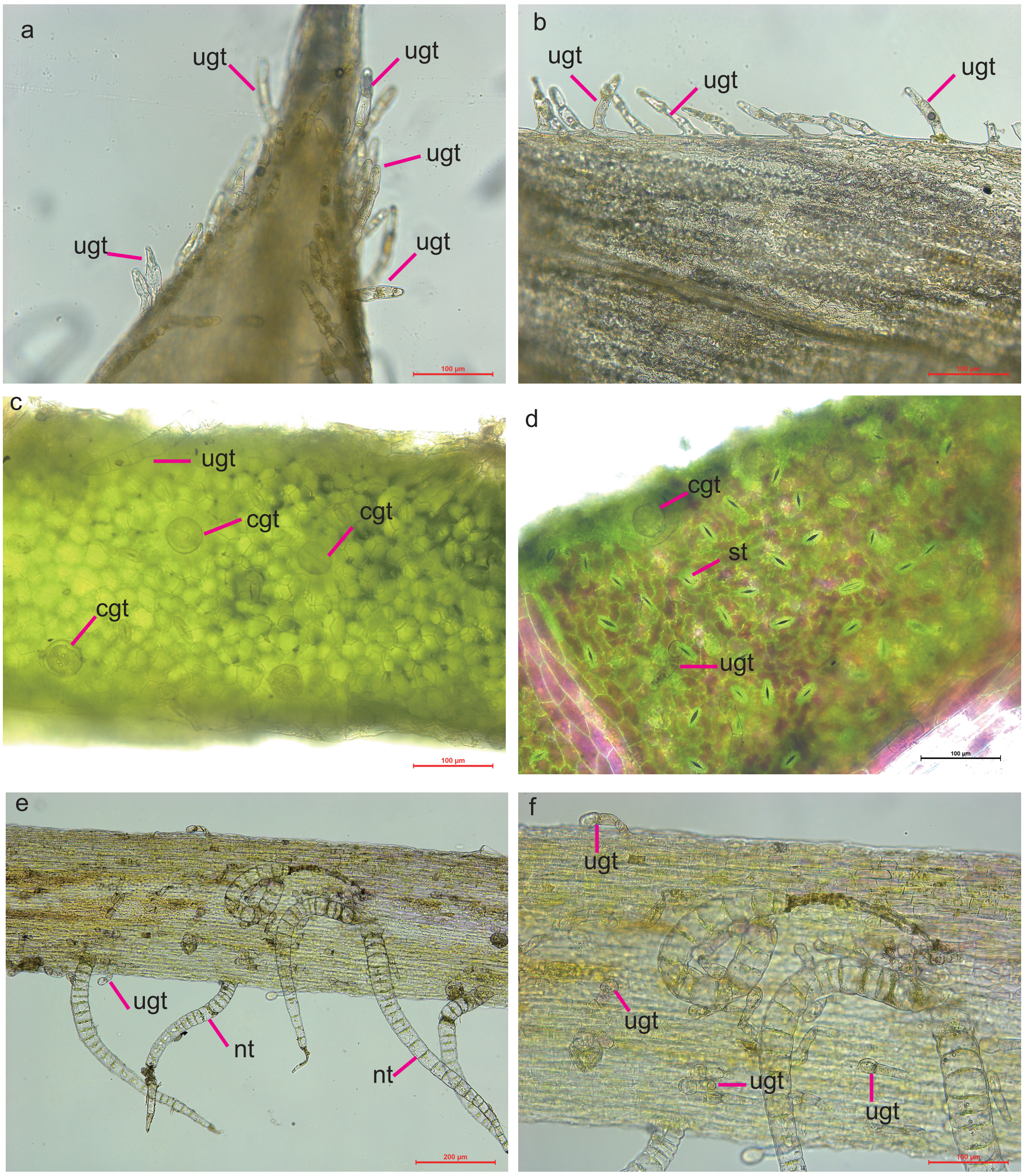

Supplementary Figure 24. Trichome in different tissues of *M. micrantha*. a-b: the petal of flower in surface view. c-d: surface view of leaf. e-f. surface view of stem; Ugt: uniseriate glandular trichome; cgt: conical glandular trichome; nt: non-glandular trichome; st: stomatum. 100 μm (a-f).

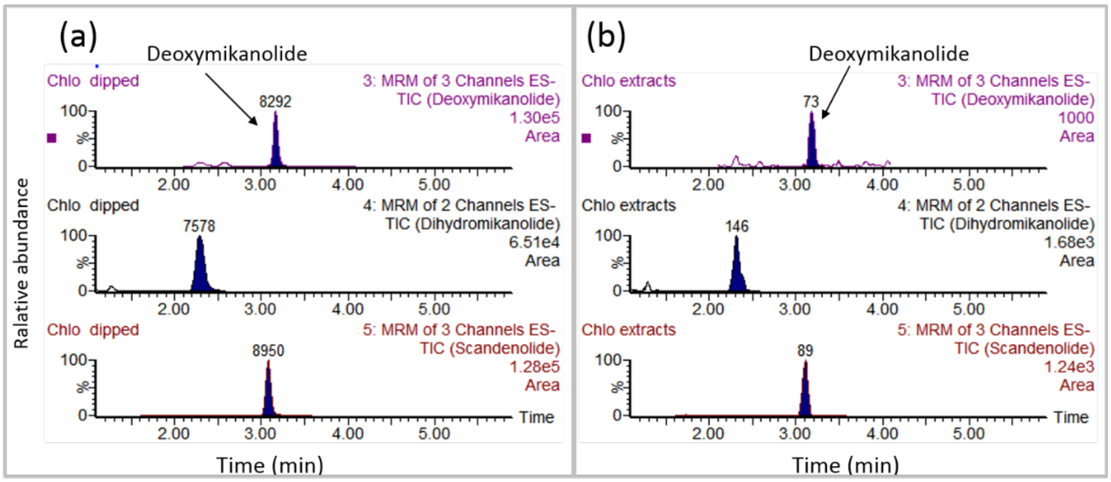

Supplementary Figure 25. Comparitive analysis of chloroform dipping extract and the residue extracts of fresh *M. micrantha* leaves. A: Multiple reaction monitoring (MRM) of scandenolide, dihydromikanolide and deoxymikanolide from chloroform dipped extracts of fresh leaves. B: MRM analyzed the STLs from the residue extract by chloroform.

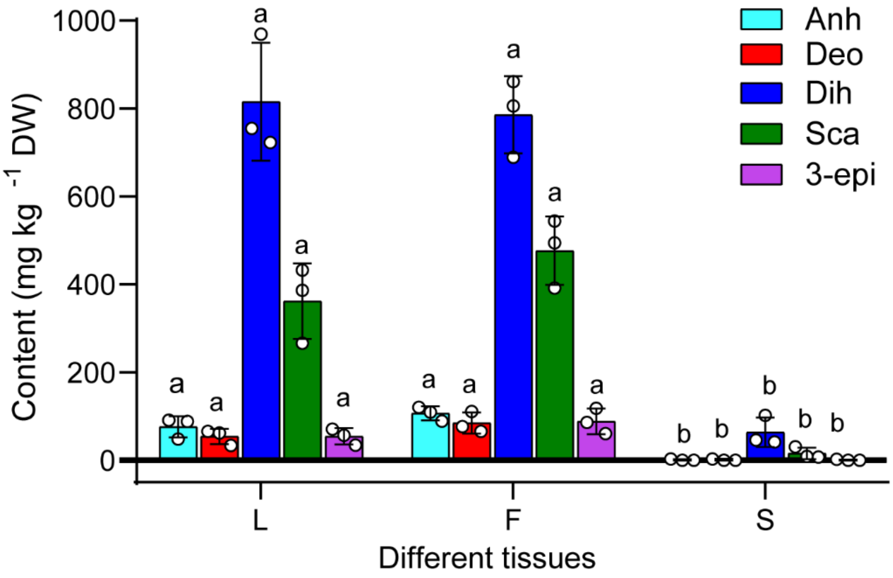

Supplementary Figure 26. Quantification of five STLs in different dried tissues of trichomes of *M. micrantha* (L: leaf, F: flower; S: stem). n=3 biologically independent samples, as determined by one-way ANOVA followed by Duncan's multiple range test, $p < 0.05$. Error bars indicate mean ± s.d. of indicated replicates. The source data is provided as a Source Data file.

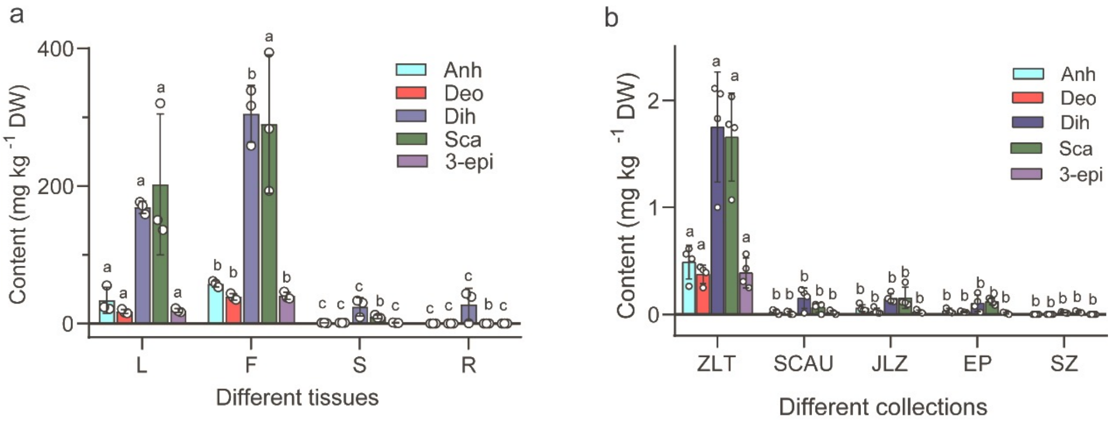

Supplementary Figure 27. Quantitative analysis of five sesquiterpene lactones in different tissues of *M. micrantha* and soil by LC-MS. a, The content of five STLs in dried parts of plant; n=3 biologically independent samples. Significant differences determined by one-way ANOVA followed by Duncan's multiple range test, $p < 0.05$. **b,** five STLs can be determined in invasive soil of *M. micrantha*. Anh, anhydroscandenolide; Deo, deoxymikanolide; Dih, dihydromikanolide; Sca, scandenolide; 3-epi, 3-epi-dihydroscandenolide. R: root; S: stem; L: leaf; and F: flower. ZLT, JLZ, SCAU, EP, and SZ were different geographic sources in Guangdong province, China (see sample collection in Supplementary Note 4). n=4 biologically independent samples, as determined by one-way ANOVA followed by Duncan's multiple range test, $p < 0.05$. Different letters indicate significant differences among means for the different treatments. Error bars indicate mean ± s.d. of indicated replicates. The source data is provided as a Source Data file.

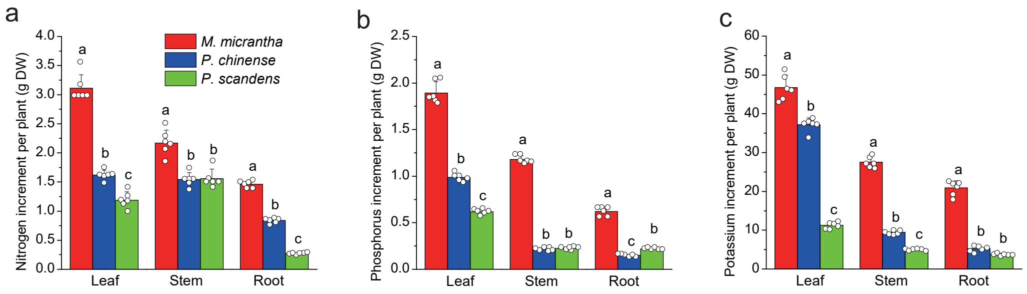

Supplementary Figure 28. Plant tissue nutrient increment of *M. micrantha* and its two neighboring natives growing for three months in the pot experiment. n=6 biologically independent samples, as determined by one-way ANOVA followed by Duncan's multiple range test, $p < 0.05$. Different letters indicate significant differences among means for the different treatments. Error bars indicate mean ± s.d. of indicated replicates. The source data is provided as a Source Data file.

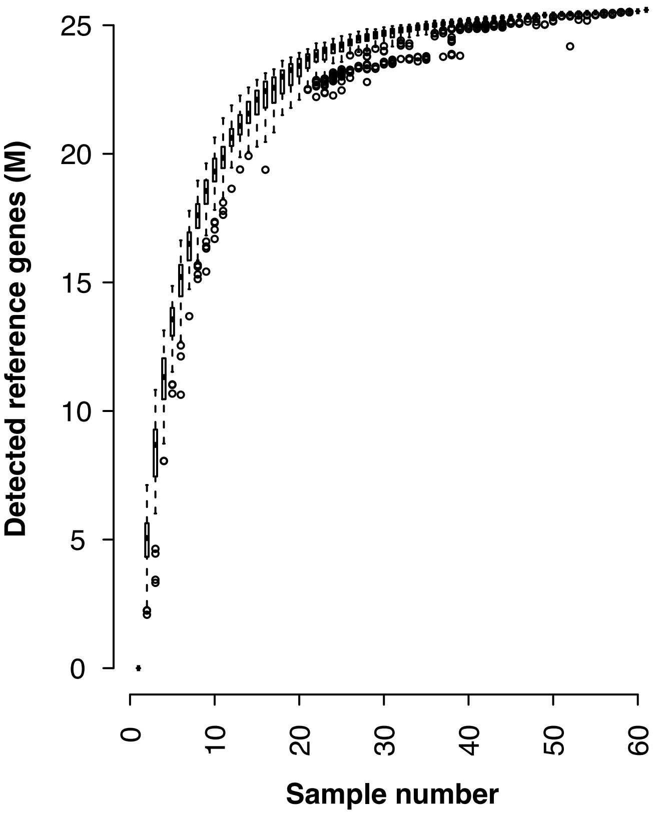

Supplementary Figure 29. The rarefaction analysis of all *M. micrantha* soil samples.

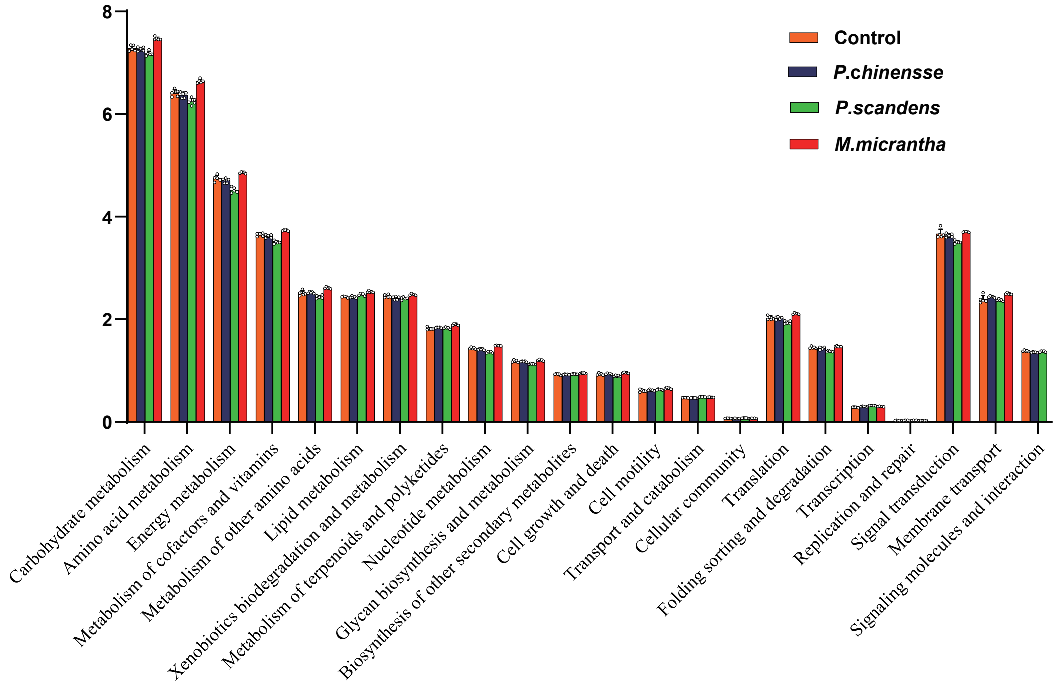

Supplementary Figure 30. The comparison of gene relative abundance in each KEGG pathway under the potting condition. According to our KEGG functional analysis, the relative abundance of root microbiota in the *M. micrantha* was enriched in the all metabolic pathways mentioned in this figure, as well as in cell growth and death, cell motility, replication and repair and membrane transport pathways. The source data is provided as a Source Data file.

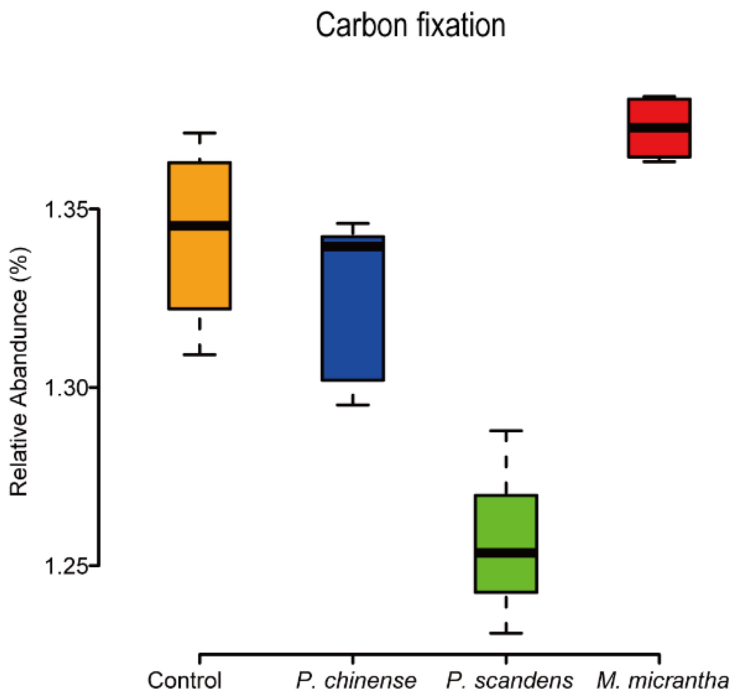

Supplementary Figure 31. The box plots of gene relative abundance in carbon fixation pathway under the potting condition. Adjusted p value was calculated using Dunn's test. n=5 biologically independent soil samples. On each box plot, the central mark indicates the median, the bottom and top edges of the box indicate the interquartile range (IQR) and the whiskers represent the maximum and minimum data points. The source data is provided as a Source Data file.

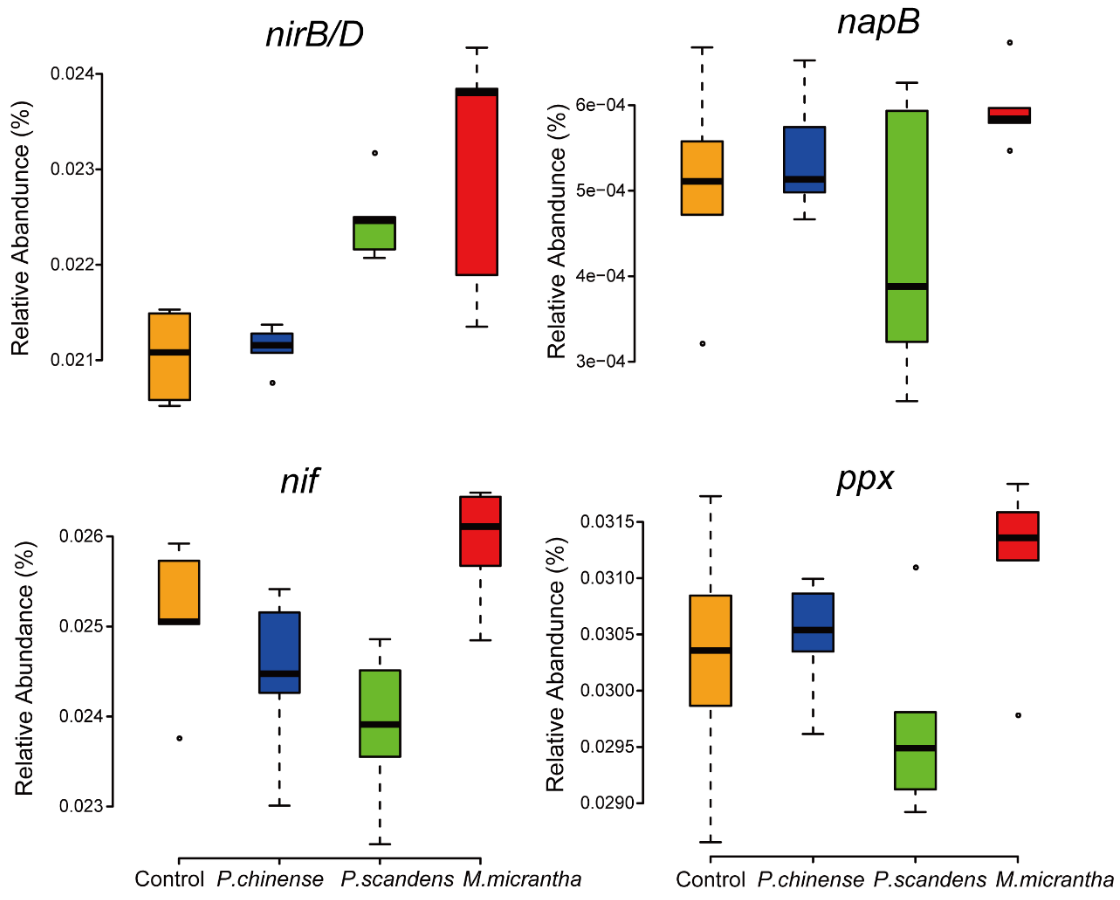

Supplementary Figure 32. The box plots of gene relative abundance, involved in nitrogen fixation genes (*nif*), assimilatory nitrogen reduction genes (*nirB/D*), dissimilatory nitrogen reduction genes (*napB*) and polyphosphate degradation genes (*ppx*), under the potting condition. n=5 biologically independent soil samples. On each box plot, the central mark indicates the median, the bottom and top edges of the box indicate the interquartile range (IQR) and the whiskers represent the maximum and minimum data points. The source data is provided as a Source Data file.

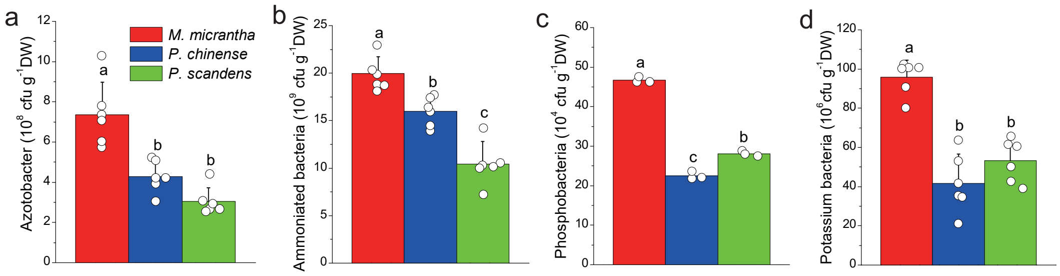

Supplementary Figure 33. Population density of soil cultured bacteria associated with nitrogen cycling, phosphorus and potassium solubilizing capacity of *M. micrantha* and its two neighboring natives in the pot experiment. n=3 or 6 biologically independent samples, as determined by one-way ANOVA followed by Duncan's multiple range test, $p < 0.05$. Different letters indicate significant differences among means for the different treatments. Error bars indicate mean ± s.d. of indicated replicates. The source data is provided as a Source Data file.

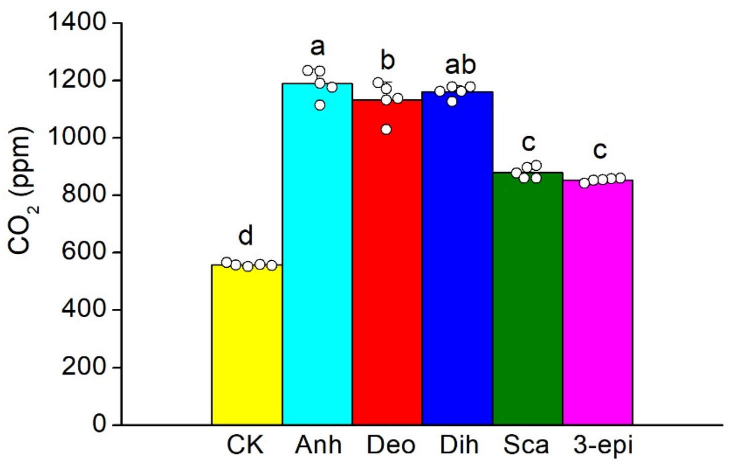

Supplementary Figure 34. Changes of CO_2 concentration after added five Mikania sesquiterpene lactones at 12 days in the uninvaded soil. Note: Anh, anhydroscandenolide; Deo, deoxymikanolide; Dih, dihydromikanolide; Sca, scandenolide; 3-epi, 3-epi-dihydroscandenolide. n=5 biologically independent samples, as determined by one-way ANOVA followed by Duncan's multiple range test, $p < 0.05$. Different letters indicate significant differences among means for the different treatments. Error bars indicate mean ± s.d. of indicated replicates. The source data is provided as a Source Data file.

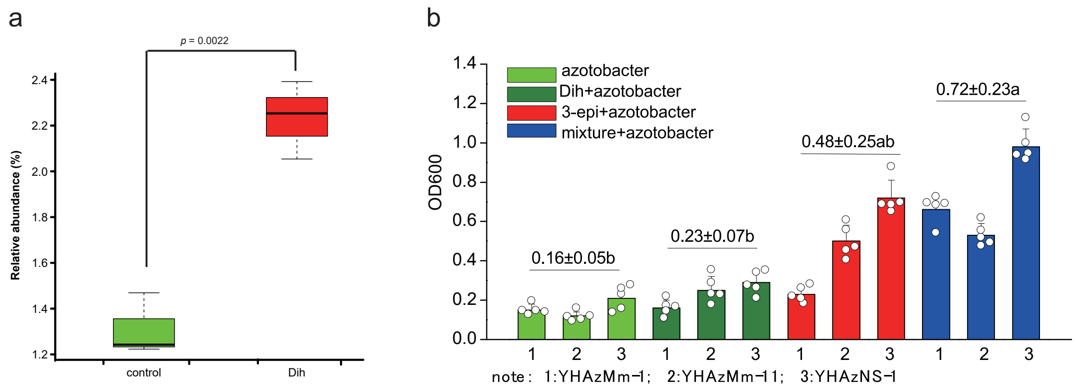

Supplementary Figure 35. Effect of STLs on the nitrogen fixation bacteria. a, We selected the soil adding Dih compound, which was the highest among the five STLs in the soil colonized by *Mikania micrantha*, for 16S rRNA sequencing. Statistical significance determined using the two-sided Student's t test method. n=3 biologically independent samples. On each box plot, the central mark indicates the median, the bottom and top edges of the box indicate the interquartile range (IQR) and the whiskers represent the maximum and minimum data points. **b,** The density of cultured nitrogen fixing bacteria was used to prove the effects of STLs on microorganisms. Adding 1mL of 2 mg/L STLs (Dih, 3-epi, and mixture of five STLs) and 0.5 mL cultured nitrogen-fixing bacteria respectively to 25mL of culture medium. Meantime, adding STL (Dih, 3-epi, and mixture of five STLs) respectively to culture medium, and adding nothing as the control. These samples were cultured in a shaker at 28C° and 150 rpm for 7 days. n=5 biologically independent samples. After then, OD values of each treatment were measured at 600 wavelengths by ultraviolet spectrophotometer (UV-2450) to evaluated the density of azotobacter. Error bars indicate mean ± s.d. of indicated replicates. The source data is provided as a Source Data file.

Supplementary Tables

Supplementary Table 1. The summary of *M. micrantha* sequencing data.

	No. of SMRT Cells	Total Base (Gb)	N50 Length (Kb)	N90 Length (Kb)	Coverage
Pacbio RSII	339	128.7	12.1	5.3	68.8
Pacbio Sequel	14	99.8	14.5	5.3	53.4
Total	353	228.5	13.3	5.3	122.2

Supplementary Table 2. The summary of contig sequences in *M. micrantha*

	Contigs		Linkage groups	
	No.	Base	No.	Base
Total Length	-	1,790,485,694	-	1,790,645,594
Maximum Length	-	18,035,607	-	124,364,672
Minimum Length	-	7,510	-	7,510
N10 Length	14	9,087,641	2	121,786,369
N20 Length	39	6,213,704	4	109,319,893
N30 Length	78	3,572,089	5	95,647,450
N40 Length	146	2,152,619	7	94,816,839
N50 Length	250	1,353,263	9	86,674,320
N60 Length	414	913,757	11	78,648,121
N70 Length	652	636,859	14	68,185,407
N80 Length	992	444,010	16	63,746,411
N90 Length	1520	180,265	19	47,241,367

Supplementary Table 3. The Evidences of gene prediction in *M. micrantha* genome.

	ab-initio	At-Homolog	Vv-Homolog	Ha-Homolog	Ls-Homolog	Pv-Homolog	RNA-Seq	Full-Length Transcriptome	EVM
Number of Gene	124,101	45,092	53,604	104,963	81,691	51,160	46,272	11,808	46,351
Average CDS Length	1,273	956	952	799	857	897	2,011	2,216	1,242
Average Number Exon of Gene	4.3	4.0	3.7	2.7	3.1	3.7	3.2	7.3	5.0
Average Exon Length	299	240	255	292	274	241	629	305	246
Average Intron Length	688	708	755	939	826	721	823	619	745

At: *Arabidopsis thaliana*; Vv: *Vitis vinifera*; Ha: *Helianthus annuus*; Ls: *Lactuca sativa*; Pv: *Phaseolus vulgaris*; The Results of EVM was the final Predicted gene.

Supplementary Table 4. The characteristic of coding genes in eight species.

	M. micrantha	sunflower	lettuce	*A. annua*	artichoke	coffee	*A. thaliana*	grape
Genome Size (Gb)	1.9	2.9	2.3	1.7	0.7	0.6	0.1	0.5
Gene Number	46,351	58,229	35,519	63,226	26,326	25,574	27,206	25,676
Avg. CDS Length	1,242	1,232	1,251	1,121	1,316	1,205	1,213	1,339
Avg. Exon Number	5.0	4.3	4.7	5.0	5.4	5.1	5.1	5.2
Avg. Exon Length (bp)	246	289	262	222	240	236	237	256
BUSCO Complete Ratio	91%	93%	92%	89%	93%	89%	93%	92%

Supplementary Table 5. Function annotation in *M. micrantha* genome

Database	Gene Number	Percentage
eggNOG	38,995	84.1%
KEGG	31,507	68.0%
Interpro	40,267	86.9%
uniprot sprot	31,436	67.8%
uniprot trembl	39,458	85.1%
Total	42,804	92.3%

Supplementary Table 6. The characteristic of transposable elements in seven species.

Species	SINEs (Mb)	LINEs (Mb)	DNA elements (Mb)	LTR elements (Mb)	Unclassified (Mb)	Total (Mb)
M. micrantha	5.88 (0.33%)	18.98 (1.06%)	99.34 (5.55%)	813.83 (45.45%)	375.5 (20.97%)	1,313.53 (73.36%)
sunflower	2.21 (0.07%)	58.81 (1.94%)	99.68 (3.29%)	1533.56 (50.65%)	549.09 (18.13%)	2,243.36 (74.08%)
lettuce	1.92 (0.08%)	35.13 (1.47%)	54.15 (2.27%)	1202.08 (50.42%)	444.13 (18.63%)	1,737.41 (72.87%)
A. annua	1.1 (0.06%)	30.52 (1.7%)	58.05 (3.24%)	524.54 (29.26%)	378.57 (21.12%)	992.78 (55.38%)
artichoke	1.27 (0.18%)	11.38 (1.57%)	37.22 (5.13%)	190.69 (26.29%)	175.62 (24.22%)	416.18 (57.39%)
coffee	0.04 (0.01%)	9.21 (1.62%)	14.32 (2.51%)	148.33 (26.03%)	71.96 (12.63%)	243.86 (42.8%)
chrysanthemum	0.68 (0.03%)	62.83 (2.49%)	81.24 (3.21%)	1,128.74 (44.66%)	485.28 (19.20%)	1,758.77 (69.59%)

The numbers in brackets indicate the percent of the total genome sequence.

Supplementary Table 7. The summary of LTR retrotransposons in *M. micrantha* genome

LTR	Count	Percent	Avg. Length	Percent of Genome
Gypsy	459,130	70.10%	1,323	33.90%
Copia	170,734	26.10%	1,281	12.20%
Others	25,041	3.80%	566	0.80%
Total	654,905	100%	1,283	46.90%

Supplementary Table 8. The comparison of GAS, GAO, Ih8H homologous in *M. micrantha*, *Helianthus annuus*, *Artemisia annua*, *Lactuca sativa* and *Inula hupehensis*.

Gene Name of *M. micrantha*	Gene Name of Relative Species	species	Identity (%)	Coverage (%)
MmGAS1 (Mm17G037281)	*HaGAS1* (XP_022010071.1)	*Helianthus annuus*	87	99.6
MmGAS2 (Mm17G037281)	*HaGAS2* (XP_022015687.1)	*Helianthus annuus*	86	99.6
MmGAS3 (Mm17G037281)	*HaGAS3* (ACZ50512.1)	*Helianthus annuus*	82	99.5
MmGAS (Mm17G037281)	*AaGAS* (ABE03980.1)	*Artemisia annua*	76	97.3
MmGAO1 (MmUnG044903)	*LsGAO* (ADF32078.1)	*Lactuca sativa*	75	100%
MmGAO1 (MmUnG044903)	*HaGAO* (ADF43082.1)	*Helianthus annuus*	79	100%
MmGAO2 (MmUnG044904)	*LsGAO* (ADF32078.1)	*Lactuca sativa*	72	100%
MmGAO2 (MmUnG044904)	*HaGAO* (ADF43082.1)	*Helianthus annuus*	73	100%
MmGAO3 (MmUnG044907)	*LsGAO* (ADF32078.1)	*Lactuca sativa*	75	100%
MmGAO3 (MmUnG044907)	*HaGAO* (ADF43082.1)	*Helianthus annuus*	78	100%
MmIh8H (Mm07G019019)	*HaG8H* (XP_022035380.1)	*Helianthus annuus*	61	98.7
MmIh8H (Mm07G019019)	*Ih8H* (AML23863.1)	*Inula hupehensis*	49	99.6

Supplementary Table 9. The candidate genes of sesquiterpene lactones (STLs) biosynthesis pathway from precursor germacrene A acid (GAA).

GeneID	Gene Expression				Gene Functions
	Root	Stem	Leaf	Flower	
Mm04G010432	0.0201761	3.24639	10.2359	22.7885	Cytochrome P450 CYP71B34
Mm04G010431	0.0492369	4.02235	17.8173	40.9775	Cytochrome P450 CYP71B34
Mm15G034569	0.17204	23.7544	84.7442	269.448	Short-chain dehydrogenase/reductase
Mm02G003954	0.0268542	4.47327	7.73847	28.0894	Cytochome b5
Mm02G003957	0.316979	3.42486	14.7958	25.4379	Cytochome b5
Mm05G011612	0.174041	4.95731	17.5	25.8304	Cytochrome P450 CYP71A4-like
Mm09G022971	0.00368511	3.29837	11.6936	52.7336	Aldehyde dehydrogenase
Mm13G031094	0.102297	6.34607	15.3216	64.6139	Alkyl transferase
Mm05G011607	0.0379987	2.84234	10.1	16.2454	Cytochrome P450 CYP71A25-like
Mm05G011608	0.0192457	3.04385	10.8369	17.2623	Cytochrome P450 CYP71A4-like

Supplementary Table 10. Changes of soil chemical characteristics in the pot experiment.

Soil samples	TP ($g\ kg^{-1}DW$)	AP ($mg\ kg^{-1}DW$)	TK ($g\ kg^{-1}DW$)	AK ($mg\ kg^{-1}DW$)
Control	0.99±0.02a	10.25±0.22c	7.02±1.24a	80.51±4.72a
P. chinense	0.92±0.01c	10.66±0.10b	5.43±0.50b	48.82±2.58c
P. scandens	0.94±0.02b	9.65±0.31d	5.63±0.56b	52.43±3.76c
M. micrantha	0.88±0.03d	11.69±0.07a	4.44±0.37c	60.21±7.24b

Control: no plants grown. n=5 or 6 biologically independent samples, as determined by one-way ANOVA followed by Duncan's multiple range test, $p < 0.05$. Different letters indicate significant differences among means for the different treatments. Error bars indicate mean ± s.d. of indicated replicates.

Supplementary Table 11. Changes of soil chemical characteristics after added chemical compound in the invaded soil.

Soil samples	TC (g kg^{-1}DW)	TN (g kg^{-1}DW)	NH_4-N (mg kg^{-1}DW)	NO_3-N (mg kg^{-1}DW)	AP (mg kg^{-1}DW)	AK (mg kg^{-1}DW)
Control	15.57±1.33b	2.71±0.30a	5.12±0.72b	28.38±4.87a	13.43±0.47d	76.15±5.67c
Dih	20.73±1.91a	2.61±0.18ab	8.13±1.47a	15.17±2.64b	15.88±0.42b	87.39±9.93bc
Anh	20.08±2.74a	2.29±0.42b	8.34±1.75a	15.61±2.67b	14.75±0.36c	97.45±17.71ab
Deo	18.97±1.54a	2.27±0.20b	7.02±1.17a	10.41±0.75cd	14.82±0.43c	107.64±11.76a
Sca	20.78±2.07a	2.27±0.24b	8.79±1.18a	13.66±2.74bc	8.53±0.27e	87.96±4.40bc
3-epi	19.52±2.08a	2.25±0.28b	7.80±1.50a	11.90±2.98bcd	18.11±0.07a	84.88±14.85bc

Note: Dih: dihydromikanolide, Ang: anhydroscandenolide, Deo: deoxymikanolide, Sca: scandenolide, and 3-epi: 3-epi-dihydroscandenolide. Control: did not add chemical compounds to soil. n=5 biologically independent samples, as determined by one-way ANOVA followed by Duncan's multiple range test, $p < 0.05$. Different letters indicate significant differences among means for the different treatments. Error bars indicate mean ± s.d. of indicated replicates.

Supplementary Table 12. Precursor/Product ion pairs and parameters for multiple reaction monitoring (MRM) of compounds.

Compounds	Retention time (min)	Molecular ions (m/z)	MRM transition (precursor→product)	Cone voltage (V)	Collision energy (eV)
1	1.89	273.05	273.1→185.1[a]	26.0	10.0
			273.1→229.1	26.0	8.0
2	2.65	275.06	275.1→187.1[a]	30.0	10.0
			275.1→231.2	30.0	8.0
3	1.82	291.06	291.1→219.1	28.0	18.0
			291.1→247.1[a]	28.0	8.0
4	2.56	333.10	333.1→229.1[a]	24.0	14.0
			333.1→273.1	24.0	6.0
5	2.66	335.12	335.1→187.1	26.0	16.0
			335.1→275.1[a]	26.0	8.0

[a]Transition used for quantitation.

Supplementary Note 1: genome assembly

Estimate the *M. micrantha* Genome size with K-mer

A K-mer refers to artificial sequence division of K nucleotides. A raw sequence read with L bp contains (L-K+1) K-mers if the length of each K-mer is K bp. The frequency of each K-mer can be calculated from the raw genome sequence reads. The K-mer frequencies along the sequence depth gradient follow a poisson distribution in a given data set. During deduction, the genome size G=K_num/K_depth, where the K_num is the total number of K-mer, and K_depth is the frequency occurring more frequently than the others [1]. The genome size of *M. micrantha* was estimated to be ~1.87 Gb (Supplementary Figure 2).

Genome Assembly and Filtering out heterozygous fragments

The *M. micrantha* genome assembly was used a combination strategy (Supplementary Figure 1). First, the PacBio reads were used to assemble the *M. micrantha* genome by the software canu-1.6, and 7,523 contigs were generated with a total length of 2.16 Gb. Based on the distribution of k-mer frequencies, the estimated genome size of *M. micrantha* is 1.87 Gb. Then, we used the following methods to filter the heterozygous sequences: Firstly, we used the genome sequence to align to itself by MUMmer3.23 [2], and filtered out the heterozygous segments with more than 70% coverage and 70% identity. Secondly, the PacBio reads were re-assembled by falcon/falcon-unzip, which generated 1,684 Mb primary reference sequence and 352 Mb alternative heterozygous haplotype sequence (haplotig). Then, we aligned the 352 Mb alternative heterozygous haplotype sequence (haplotig) to the canu assembled contigs, to further filter the heterozygous sequences in the canu reference sequences. The resulting 4,414 contigs with a total length of 1.79 Gb were used as the final haploid reference genome of *M. micrantha*.

Supplementary Note 2: the leaf photosynthesis pathways

Electron microscopic observations of leaf stomata

Leaves were fixed in 2.5% glutaraldehyde and 2% paraformaldehyde, and then dehydration through a graded ethanol series (30%, 50%, 70%, 80%, 90% and 100%). The dehydrated leaves were

critical-point dried with liquid carbon dioxide and then sputtered with a 30-nm gold layer. Scanning electron microscopy (SEM) images of the samples were taken using a scanning electron microscope (SEM, Q25, FEI, USA).

Change of stomatal conductance of leaf

The stomatal conductance of *M. micrantha* leaves was measured by LI-6400 portable photosynthesis analyzer (LI-COR, Inc, USA) at 9:00 a.m. and 9:00 p.m. on sunny days. The light source uses the red and blue LED light source with the leaf chamber standard, and sets the light intensity to 800 μmol m^{-2} s^{-1}. The concentration of CO_2 was set to 400 μmol m^{-2} s^{-1}. Leaves were fully induced under 800 μmol m^{-2} s^{-1} light intensity. Data were recorded after the photosynthesis of leaves reached its optimum state. Gas conductance (Gs) of leaves was calculated and stored automatically by the instrument.

Detection of PEPC and NADP-ME activity and organic acid content

PEPC activity. Extraction of enzyme solution: Weighted 0.3g leaves of *M. micrantha*, and grinded with grinding buffer (containing pH 8.2 0.1M Tris-HCl, 7 mM mercaptoethanol, 1 mM EDTA-Na_2, 5% (W/V) glycerol, 1% (W/V) PVP-10). Subsequently centrifuged for 10 minutes at 13,000g 4°C, and then extracted supernatant for related detection.

Determination on PEPC activity. The total volume of the reaction solution was 3.1 mL, containing 1 mL enzyme reaction buffer (pH 8.2), 100 μL enzyme solution, 100 μL 10 mM $MgCl_2$, 100 μL 10 mM $NaHCO_3$, 300 μL 0.1 mg L-1 NADH (pH 8.9), 300 μL malate dehydrogenase, 1 mL Tris-HCl (pH 9.2). The optical density (OD1) was measured at 340 nm. The reaction was initiated by adding 200 μL 40mM PEP, and the optical density (OD2) was recorded immediately after 1 minute.

NADP-ME activity. The activity of NADP-ME was determined according to the instructions of the kit (Beijing Solarbio Science & Technology Co., Ltd., Beijing, China).

Extraction of enzyme solution: 0.1g tissue was weighed and 1 mL extract was added to homogenize in ice bath. The supernatant was centrifuged at 4°C, 8 000 g for 10 min.

Determination on NADP-ME activity. The total volume of the reaction solution was 900 uL, containing 600 uL Reagent 1, 270 uL working solution and 30 uL enzyme solution. The optical density (OD1) was measured at 340 nm and the optical density (OD2) was recorded immediately after 1 minute. The soluble protein content was determined by Coomassie brilliant blue G250 method.

Organic acid content. Referring to Flores et al. (2012) [3] method, 0.3g frozen sample was weighed in centrifugal tube, frozen and stirred in grinder, then homogenized with 1.5mL water. After mixing on oscillator for about 1 minute, it was put into centrifuge for 8000g, centrifuged for 10 minutes, 4°C. The supernatant was analyzed immediately after passing through microporous membrane (0.45 um) or put into refrigerator at 4°C for testing. The organic acids in leaves were determined by ultra-high performance liquid chromatography-mass spectrometry (Xevo TQD, Waters MS Technologies, Manchester, UK). The standard reserve liquids of malic acid and citric acid with concentration ranging from 1 to 50 mg/L were determined and the standard curves were drawn. The regression equation and linear coefficients were calculated, and the contents of organic acids in the samples were obtained.

$\delta^{13}C$ determination

According to the method of [4], the leaf samples of day and night were baked at 65°C to constant weight, ground and sifted through 60 meshes. The 10 mg sample was placed in a quartz tube, a small amount of copper oxide was added, platinum (PR) was used as catalyst, sealed in vacuum, and then reacted at 800°C for half an hour. Carbon isotope ^{13}C and ^{12}C were determined by elemental analyzer-isotope mass spectrometer (Vario isotope cube -Isoprime 100). Based on the carbon isotope ratio of PDB fossils, the carbon isotope ratio of PDB fossils was determined by the value of $\delta^{13}C(-‰)$ =(R sample/R standard-1)*1000 was calculated.

Supplementary Note 3: stem photosynthesis

We conducted the exploration test of stem photosynthesis of *M. micrantha* during March to June in 2018, and the experiment of comparison with associated specie *(Paederia scandens*) and

transcriptome analysis of *M. micrantha* were completed during July to December, at the South China Normal University in Guangzhou, China.

Plant Materials. Vigorous stems of *M. micrantha* were collected from several wild populations near the Agricultural Genomics Institute at Shenzhen, Chinese Academy of Agricultural Sciences. *P. scandens* was collected from Guangdong Academy of Agricultural Sciences, in China.

Stems of *M. micrantha* and *P. scandens* were cut into small segments (about 10 cm) with two nodes, and all leaves of the nodes were removed. The segments were cultured in fresh water, with the lower nodes submerged. Following approximately 2 weeks, all segments had produced adventitious roots in the lower nodes, and sprouted one or two new shoots in upper nodes. Vigorous segments with at least one new shoot were selected and planted in plastic plots (one plant per plot). 2 weeks later, 20 plots of uniform seedlings (about 50 cm high) were selected and one half were conducted experimental treatment.

Determination of photosynthetic pigments and anthocyanin

Three 10-mm-diameter leaf discs and 2-cm-length stem (the third lobe from the top to down, the diameter was measured by Electronic vernier calipers) were homogenized respectively using a mortar and pestle in 2 mL and 4 mL of 80% acetone, and then placed at 4℃ in the dark overnight for chlorophyll extraction. After blended and centrifugated at 8,000g for 10 min, the contents of Chl a, Chl b and total Chl were calculated according to Wellburn (1994) [5].

Three leaf discs (10 mm in diameter) 1-cm-length stem (the third lobe from the top to down, the diameter was measured by Electronic vernier calipers) were respectively submerged in 4 mL of methanol : HCl (99:1, v/v) at 4 °C in the dark for 24 h for extraction of anthocyanin pigments. Subsequently, 4-mL chloroform and 1.5-mL deionized water were added to the extract for removal of chlorophylls. After blending, anthocyanins were dissolved in the upper layer of solution, whereas chlorophylls were dissolved in the lower solution. The volume of the upper layer solution was determined, and its absorbance was measured at 530 nm against methanol: HCl (99:1, v/v) as a blank. Canidin-3-O-glucoside (5–200 μM) was used as the standard for calibration of anthocyanin concentration.

Gas exchange measurements

A portable infrared gas analyser LI-6800 (LI-COR, Inc., USA) was used to measure gas exchange parameters of leaves and stems in the morning (8:30–12:00). During measurements of net photosynthetic rate (Pn) at the saturating photosynthetic photon flux density (PPFD) 800 μmol m^{-2} s^{-1}, and of dark photosynthetic photon flux density (PPFD) of 800μmol m^{-2} s^{-1} was emitted from a red and blue (9:1) LED light source integrated into the LI-6800 leaf measurement chamber. The cover area of leaves was a const 9 cm^2, and the cover area of stems was input by manual after measurement. The CO_2 concentration flowing into the leaf chamber was controlled at 400 μmol mol^{-1}, leaf and stem temperature were maintained at ~30 °C and humidity was 60~70%. Dark respiration rate (Rd) was equal to the absolute value of Pn measured at 0 μmol m^{-2} s^{-1} of PPFD, 400 μmol mol^{-1} of CO_2 and 30°C of temperature. The gross photosynthetic rate (Pgross) at PPFD 800 μmol m^{-2} s^{-1} was calculated as Pgross = Pn + Rd

Chloroplast ultrastructure

Fresh leaves and stems were cut into pieces (1mm×1mm×1mm), and doubled fixed with 4% glutaraldehyde and 1% O_SO_4. And then dehydrated in graded ethanol solution and embedded with EP 812 resin. The slices were prepared by an ultramicrotome (Leica UC7, Leica) at 60-80 nm and double stained with uranium and lead (containing aqueous uranyl acetate followed by 6% lead citrate, each stained for 15 minutes). The stained slices were dried overnight. The ultrastructure of chloroplasts was observed by transmission electron microscopy (HT7700, Hitachi Japan)

Supplementary Note 4: identification of secondary metabolites in *Mikaina micrantha*

Instruments and reagents

NMR spectra were recorded by a Bruker AVANCE-600 (600 MHZ) Instrument (Bruker Biospin, Zurich, Switzerland). UPLC-TQD-MS was operated using an Acquity UPLC system (Waters Corporation, Milford, MA, USA) coupled with a MS (Xevo TQD, Waters MS Technologies, Manchester, UK), and controlled by MassLynx v4.1 software (Waters Corp., Milford, MA, USA). HRESIMS spectrum were obtained from UPLC-QTOF-MS (Agilent, Inc. Santa Clara, CA, U.S.A.).

HPLC separations used a Agilent 1100 HPLC equipped with a UV detector using a semi-preparative column (Zorbax 300 SB-C18 column, 9.4 mm ×25 cm, 4 μm). Sephadex LH-20 (25-100 μm; Amersham Bioscieces, Sweden), HPLC grade acetonitrile, methanol and formic acid were purchased from J. T. Baker (Philipsburg, NJ, USA). Constunolide was purchased from BioBioPha Co., Ltd. (Kunming, China). Alantolactone and isoalantolactone were purchased from ShanghaiyuanyeBio-TechnologyCo.,Ltd (Shanghai, China).

Sample collection

Mikania micrantha samples and soils were collected in different places in Guangdong province, all the samples collected in 2018, the detail information as bellows: ZLT: Zhongluotan town (N: 23° 24′4.43″. E: 113°25′36.26″); JLZ: Jiulong town (N: 23°22′15.79″. E: 113°28′6.26″); EP: Enping city (N: 22°04′34.30″. E: 112°13′52.60″); SZ: Shenzhen city (N: 22°35′59.26″. E: 114°29′18.52″); SCAU, South China Agricultural University (N:23°09′24.66″. E:113°20′59.04″). All the voucher specimens have identified by Prof. Tieyao Tu (South China Botanical Garden), have been deposited in the Herbarium of the College of National Resources and Environment, South China Agricultural University, China.

Plant extraction: 0.2 g of fresh roots, stems, stem tips, leaves, and flowers of plant (while 0.1 g of dry plant materials) were transferred in 2 mL centrifuge tubes, and ground into powder by high flux grinder under liquid nitrogen freezing. Then each tube was stirred with 2 mL 80% ethanol followed by ultrasonic extraction for 30 min in a water bath at room temperature. All the extraction solutions store at 4°C refrigerator for 12 h. Finally, the samples were centrifuged at 15,285 g for 2 min, the supernatant were directly used for the quantification of target compounds by UPLC-MS analysis.

Chloroform dipping

The extraction of STLs in glandular trichomes of *M. micrantha* tissues was conducted by dichloroformethane-dipped method as previously described [6]. Briefly, 400 mg of *M. micrantha* different tissues were dipped in 20 mL of dichloroformethane by vortexing for 30 s. The dichloroformethane phase was filtered and concentrated by rotary evaporator under vacuum to

obtain the crude extract. The extracts were then dissolved in 4 mL solution (methanol:dichloroformethane=3.5:0.5, v/v) and measured by UPLC-MS.

Soil extraction

The invasive soil about 1-3 cm deep surrounding the *M. micrantha* root, the collected soil samples were dried in the dark at room temperature. Plant residues were carefully removed with a sieve (30 mesh), and 15 g of soil samples were extracted with 30 mL methanol by ultrasonication for twice (30 min each) at room temperature. The filtrate was concentrated in vacuum by rotary evaporator to obtain dry residue, and then was dissolved in 1.5 mL methanol and filtered by a 0.25 μm PTFE filter prepare for the UPLC-MS analysis.

The isolation of STLs from *M. micrantha*

Aerial parts of *M. micrantha* were collected from Zhongluotan town, Guangzhou city at June, 2018. The air-dried (10 kg) were ground into powder and extracted with 95% ethanol soak for 48 h (three times). The filter solution was concentrated under reduced pressure by a rotary evaporator at 40 °C to yield the crude extract (1100 g). The extract was suspended in H_2O and partitioned with petroleum ether, chloroform, and ethyl acetate, respectively. The chloroform portion (310 g) was subjected to silica gel column chromatography eluted with a gradient of acetone in petroleum ether to affording 8 fractions (Fc1-Fc8). Fc4 was chromatographed on silica gel column by gradient system of chloroform-methanol, to obtain 2 (43.5 mg) and 3 (13.6 mg). Fc6 was repeatedly chromatographed over by silica gel column and Sephadax LH-20 to yield 1 (35.1 mg). Fractions Fc 7 and Fc8 by repeatedly column chromatography led to the isolation of 4 (13.2 mg) and 5 (9.8 mg). The chemical structure of compounds 1-5 were elucidated using NMR, HR-ESI-MS and compare NMR data to the references, which have identified as anhydroscandenolide (1)[7,8], deoxymikanolide (2)[9,10], dihydromikanolide (3)[8,9], scandenolide (4)[11], 3-epi-dihydromikanolide (**5**)[10]. The purity of compounds 1-5 were higher than 95 % by UPLC-MS and ^{1}H NMR analysis. The spectroscopic data of five known STLs as bellowing:

Anhydroscandenolide (1): white crystals, ESI-MS *m/z* 273.05 $[M - H]^-$; ^{1}H NMR (500 MHz, $CDCl_3$) δ: 3.28 (1H, dq, J = 4.7, 3.4 Hz, H-1), 6.52 (1H, ddd, J = 10.4, 3.1, 1.9 Hz, H-2a), 6.13 (1H,

d, J = 10.4 Hz, H-3), 7.42 (1H, m, H-5), 5.22 (1H, dt, J = 4.8, 1.9 Hz, H-6), 3.72 (1H, t, J = 1.5 Hz, H-7), 4.63 (1H, ddd, J = 10.8, 8.1, 5.6 Hz, H-8), 2.18 (1H, dd, J = 13.8, 10.7 Hz, H-9a), 2.33 (1H, dd, J = 14.0, 5.6 Hz, H-9b), 6.49 (1H, d, J = 3.5 Hz, H-13a), 6.06 (1H, d, J = 3.1 Hz, H-13b), 1.13 (3H, s, Me-14). ^{13}C NMR (125 MHz, $CDCl_3$) δ: 59.2 (C-1), 123.8 (C-2), 135.0 (C-3), 131.1 (C-4), 151.0 (C-5), 82.2 (C-6), 49.5 (C-7), 76.7 (C-8), 43.8 (C-9), 58.7 (C-10), 136.9 (C-11), 167.7 (C-12), 125.1 (C-13), 22.5 (C-14), 170.3 (C-15).

Deoxymikanolide (2): colerless crystals, ESI-MS *m/z* 275.06 $[M - H]^-$; ^{1}H NMR (500 MHz, $CDCl_3$) δ: 2.77 (1H, dd, J = 13.3, 5.9 Hz, H-1), 1.56 (1H, m, H-2a), 2.56 (1H, td, J = 12.9, 6.3 Hz, H-2b), 3.38 (1H, dtd, J = 4.8, 3.5, 1.4 Hz, H-3a), 2.15 (1H, m, H-3b), 7.18 (1H, s, H-5), 5.25 (1H, d, J = 1.7 Hz, H-6), 2.66 (1H, dd, J = 11.4, 2.3 Hz, H-7), 4.47 (1H, ddd, J = 11.2, 8.6, 4.5 Hz, H-8), 2.10 (1H, dd, J = 14.2, 11.2 Hz, H-9a), 2.21 (1H, dd, J = 14.3, 4.5 Hz, H-9b), 6.48 (1H, d, J = 3.7 Hz, H-13a), 5.91 (1H, d, J = 3.2 Hz, H-13b), 1.22 (3H, s, Me-14).

Dihydromikanolide (3): colorless granular crystal, ESI-MS *m/z* 291.06 $[M - H]^-$; ^{1}H NMR (500 MHz, DMSO) δ: 3.20 (1H, s, H-1), 3.39 (1H, dd, J = 3.6, 1.0 Hz, H-2), 3.99 (1H, d, J = 3.6 Hz, H-3), 7.62 (1H, d, J = 1.2 Hz, H-5), 5.43 (1H, d, J = 1.7 Hz, H-6), 2.46 (1H, m, H-7), 4.65 (1H, td, J = 10.6, 4.6 Hz, H-8), 2.08 (1H, dd, J = 13.6, 10.8 Hz, H-9a), 1.86 (1H, dd, J = 13.7, 4.6 Hz, H-9b), 2.96 (1H, dd, J = 11.8, 7.0 Hz, H-11), 1.26 (3H, d, J = 7.0 Hz, Me-13), 0.96 (3H, s, Me-14). ^{13}C NMR (125 MHz, DMSO) δ: 57.3 (C-1), 54.7 (C-2), 52.1 (C-3), 128.6 (C-4), 149.9 (C-5), 81.7 (C-6), 50.2 (C-7), 76.6 (C-8), 42.1 (C-9), 57.4 (C-10), 41.0 (C-11), 176.0 (C-12), 13.2 (C-13), 20.8 (C-14), 171.0 (C-15).

Scandenolide (4): white crystals, ESI-MS *m/z* 333.10 $[M - H]^-$; ^{1}H NMR (500 MHz, Pyridine-d_5) δ: 3.29 (1H, dd, J = 11.5, 2.2 Hz, H-1), 2.02 (1H, m, H-2a), 2.49 (1H, dt, J = 14.9, 2.1 Hz, H-2b), 6.10 (1H, br d, J = 3.8 Hz, H-3), 7.99 (H, s, H-5), 5.67 (1H, br d, J = 1.6 Hz, H-6), 3.73 (1H, dd, J = 8.4, 1.2 Hz, H-7), 4.96 (1H, m, H-8), 2.23 (1H, dd, J = 13.9, 11.2 Hz, H-9a), 2.34 (1H, dd, J = 14.0, 4.3 Hz, H-9b), 6.52 (1H, d, J = 3.7 Hz, H-13a), 6.02 (1H, d, J = 3.3 Hz, H-13b), 1.38 (3H, s, Me-14), 2.17 (3H, s, 3-OAc). ^{13}C NMR (125 MHz, Pyridine-d_5) δ: 59.1 (C-1), 30.1 (C-2), 67.9 (C-3), 133.5 (C-4), 148.2 (C-5), 83.2 (C-6), 50.7 (C-7), 78.8 (C-8), 43.8 (C-9), 57.6 (C-10), 137.9 (C-11), 168.4 (C-12), 123.1 (C-13), 21.1 (C-14), 170.6 (C-15), 169.9 (3-OAc), 20.6 (3-OAc).

3-epi-Dihydroscandenolide (5): colorless crystal, ESI-MS *m/z* 335.12 $[M - H]^-$; ^1H NMR (500 MHz, DMSO) δ: 2.96 (1H, dd, J = 11.6, 2.1 Hz, H-1), 2.23 (1H, dt, J = 14.8, 2.0 Hz, H-2a), 2.64 (1H, dd, J = 12.6, 9.8 Hz, H-2b), 5.59 (1H, br d, J = 3.7 Hz, H-3), 7.97 (1H, br s, H-5), 5.42 (1H, s, H-6), 1.66 (1H, ddd, J = 14.9, 11.6, 4.4 Hz, H-7), 4.59 (1H, dt, J = 10.9, 4.1 Hz, H-8), 1.92 (1H, dd, J = 14.1, 4.1 Hz, H-9a), 2.05 (1H, dd, J = 14.0, 11.1 Hz, H-9b), 2.92 (1H, dt, J = 13.9, 6.9 Hz, H-11), 1.26 (3H, d, J = 7.0 Hz, Me-13), 1.08 (3H, s, Me-14), 2.17 (3H, s, 3-OAc).

Quantitative analyzed STLs by UPLC-MS: Quantitative analysis of samples was performed on an ACQUITY™ UHPLC system couple with a triple-quadrupole Xevo TQD mass spectrometer. An ACQUITY UPLC® BEH C18 column (2.1 mm × 50 mm, 1.7 μm) was employed and the column temperature was maintained at 40 °C. The gradient elution with acetonitrile containing 0.1% formic acid (A) and water containing 0.1% formic acid (B), was performed as follows: 0-1.0 min, 20% A; 1.0-3.0 min, 20-60% A; 3.0–6.0 min, 60-95% A; 6.0-8.0 min, 95% A; 8.0-8.5 min, 95-20% A; 8.5-10.0 min, 20% A. The flow rate was set at 0.3 mL/min. The auto-sampler was conditioned at 22 °C and the injection volume of solution was 2 μL for analysis. Mass spectrometric detection was performed on Xevo-TQD equipped with an electrospray ionization source (ESI). The capillary voltages were set to 3.0 and 2.22 KV at positive and negative modes, respectively, the source temperature was maintained at 150 °C. The collision gas was Ar, and N_2 gas was used as desolvation at temperature of 400 °C and cone gas at a flow rate of 700 L/h, the cone gas set to 50 L/h. Compounds 1-5 were optimized in multiple reaction monitoring (MRM) in negative mode, the dwell time was 0.025 s (Table 2). MS scan and selected ion recording (SIR) also used in relative quantitative analysis in both positive and negative ions measures. Targetlynx (Waters Corp.) software was used to analyze the data.

Pot Experiment

Aerial part of *M. micrantha* was collected from South China Agriculture University and cut into 20 cm with two leaves for each (similar growth-trend), then cultivated in plant nutrient solution in a growth chamber. The plant took roots after 10 days and transplanted into vinyl pots with a diameter of 12 cm (1.0 kg of soil and three plants per pot). The soil used was a non-invasive soil collected

from Jiulong town, Guangzhou. All the pots were covered by plastic sheets to prevent the residue from aerial part except negative controls. Pots were arranged as a randomized complete block with four replicates per experiment. The plants were maintained in a greenhouse at 30 °C and 70-80 % humidity. After 10 days, four treatments were conducted as bellow: 1) Add leaves, each pot was added 5.0 g of dried and fragmentized *M. micrantha* leaves on soil surface, which aims to measure STLs in the soil mainly come from fallen leaves. 2) Add flowers, adding 5.0 of dried flowers; to measure STLs result from fallen flowers. 3) Nnothing added, adding nothing, but covered with plastic sheets to separate underground and aerial part of plants. This measure STLs in soil come from root exudates. 4) Leaching, similar to treatment of nothing added except watering, the cap (plastic sheets) was removed when leaching with simulated rain to water pots from aerial part, its aims to determine the STLs are mainly derived from leaching. 5) Natural growth, without specific treatment. The soil samples were collected by rounded puncher (1.5 cm in diameter) and gather depth at 4 cm, after the treatment of 15 days.

Supplementary Note 5: the soil's microbial structure

Five STLs addition experiment

Five STLs was added into the uninvaded soil near the invader *M. micrantha* monoculture. After 12 days of incubation, CO_2 concentration was measured by Agilent 7890B Gas Chromatograph (Agilent Technology, USA). TC and TN was determined by TOC Elemental analyzer (LI-8100A, German elementar company); NH_4^+ and NO_3^- by continuous flow analyze (Proxima, Shenzhen E-Zheng tech Co., Ltd); AP by spectro-photometer (UV-2000, Shimadzu, Kyoto, Japan); AK by flame atomic absorption spectrometry (Z-5300, Polarized Zeeman Atomic, Absorption Spectrophotometer).

Pot experiment

M. micrantha and its two neighboring native species, *P. chinense* and *P. scandens*, was respectively planted in the pot filled with natural field soil collected from the uninvaded area near the invader *M. micrantha* monoculture which located in the dry riverbed of Liuxi River, Guangzhou City, Guangdong Province, China (lat. 23°28′ N, long. 113°28′ E). Four treatments (three plants plus a

blank control) were replicated 6 times (two plants per pot with 7 kg fresh soil) and put in a greenhouse where the side windows had been opened but essentially under a glass roof in South China Normal University. Three months after the setup, the leaves, stems, and roots were separated from each plant and dried to a constant weight for at least 48 h at 60 °C. After drying and grinding leaves, stems and roots respectively, and sieving them with 0.15 mm sieve, nitrogen content per unit of mass was determined by TOC analyzer (LI-8100A, German Elementar Company), phosphorus content per unit of mass was determined by spectro-photometer (UV-2000, Shimadzu, Kyoto, Japan) [12], and potassium content per unit of mass was determined by flame atomic absorption spectrometry (Z-5300, Polarized Zeeman Atomic. Absorption Spectrophotometer). The total nitrogen, phosphorus and potassium content of each part is equal to nitrogen, phosphorus or potassium content per unit of mass multiply by biomass of each part, respectively. Nitrogen, phosphorus or potassium accumulation increment in plants is calculated by nitrogen, phosphorus or potassium content in leaves, stems and roots at harvest time minus them at transplanting seedling from the start. Total nitrogen in soil [13] was determined by TOC analyzer (LI-8100A, German Elementar Company). The ammonium and nitrate nitrogen of the soil [13] were measured by a continuous flow analyzer (Proxima, Alliance instruments, France). Total P and available P in soil were determined by using spectro-photometer (UV-2000, Shimadzu, Kyoto, Japan) according to molybdenum blue method of Murphy and Riley (1962) [14]. Available potassium was analyzed with flame atomic absorption spectrometry (Z-5300, Polarized Zeeman Atomic. Absorption Spectrophotometer). The population density of microbial community in rhizosphere soil of three plant species was detected using the dilution plate counts method [15,16].

Collection of soil samples

To collect the soil samples from *M. micrantha*, *P. chinense* and *P. scandens* in pot experiment, we randomly selected five replicates of each treatment, totally collected 20 samples. Plants were removed carefully and shaken lightly; then, the soil remaining attached to the root surface was collected with sterile water. The separated soil solution was centrifuged at 6,793 g for 10 min to collect soil samples. The collected soils were stored at −80 °C until use for microbial community analysis. We also used the same method to collected 20 filed soil samples in the dry riverbed of

Liuxi River in Guangzhou City and the reservoir of Dapeng in Shenzhen City respectively, totally 40 filed samples.

DNA extraction, library preparation, and sequencing

A combination of lysis steps was applied before DNA extraction. Next, DNA was extracted from all samples using the PowerSoil DNA isolation kit following the manufacturer's protocol (MO BIO Laboratories, QIAGEN Inc., USA). The library constructed using TruSeq DNA PCR-Free Library Prep Kit as per standard protocol (illumina, USA). Sequencing was performed on Illumina HiSeq 2500.

Supplementary Reference

1 Li, R., Fan, W., Tian, G., Zhu, H., He, L., Cai, J., Huang, Q., Cai, Q., Li, B., Bai, Y., Zhang, Z., Zhang, Y., Wang, W., Li, J., Wei, F., Li, H., Jian, M., Li, J., Zhang, Z., Nielsen, R., Li, D., Gu, W., Yang, Z., Xuan, Z., Ryder, O. A., Leung, F. C., Zhou, Y., Cao, J., Sun, X., Fu, Y., Fang, X., Guo, X., Wang, B., Hou, R., Shen, F., Mu, B., Ni, P., Lin, R., Qian, W., Wang, G., Yu, C., Nie, W., Wang, J., Wu, Z., Liang, H., Min, J., Wu, Q., Cheng, S., Ruan, J., Wang, M., Shi, Z., Wen, M., Liu, B., Ren, X., Zheng, H., Dong, D., Cook, K., Shan, G., Zhang, H., Kosiol, C., Xie, X., Lu, Z., Zheng, H., Li, Y., Steiner, C. C., Lam, T. T., Lin, S., Zhang, Q., Li, G., Tian, J., Gong, T., Liu, H., Zhang, D., Fang, L., Ye, C., Zhang, J., Hu, W., Xu, A., Ren, Y., Zhang, G., Bruford, M. W., Li, Q., Ma, L., Guo, Y., An, N., Hu, Y., Zheng, Y., Shi, Y., Li, Z., Liu, Q., Chen, Y., Zhao, J., Qu, N., Zhao, S., Tian, F., Wang, X., Wang, H., Xu, L., Liu, X., Vinar, T., Wang, Y., Lam, T. W., Yiu, S. M., Liu, S., Zhang, H., Li, D., Huang, Y., Wang, X., Yang, G., Jiang, Z., Wang, J., Qin, N., Li, L., Li, J., Bolund, L., Kristiansen, K., Wong, G. K., Olson, M., Zhang, X., Li, S., Yang, H., Wang, J. & Wang, J. The sequence and de novo assembly of the giant panda genome. *Nature* **463**, 311-317, doi:10.1038/nature08696 (2010).

2 Kurtz, S., Phillippy, A., Delcher, A. L., Smoot, M., Shumway, M., Antonescu, C. & Salzberg, S. L. Versatile and open software for comparing large genomes. *Genome Biol* **5**, R12, doi:10.1186/gb-2004-5-2-r12 (2004).

3 Flores, P., Hellín, P. & Fenoll, J. Determination of organic acids in fruits and vegetables by liquid chromatography with tandem-mass spectrometry. *Food Chemistry* **132**, 1049-1054, doi:10.1016/j.foodchem.2011.10.064 (2012).

4 Yang, H., Yu, Q., Sheng, W. P., Li, S. G. & Tian, J. Determination of leaf carbon isotope discrimination in C4 plants under variable N and water supply. *Sci Rep* **7**, 351, doi:10.1038/s41598-017-00498-w (2017).
5 Wellburn, A. R. The Spectral Determination of Chlorophylls a and b, as well as Total Carotenoids, Using Various Solvents with Spectrophotometers of Different Resolution. *Journal of Plant Physiology* **144**, 307-313, doi:10.1016/s0176-1617(11)81192-2 (1994).
6 Chen, F., Hao, F., Li, C., Gou, J., Lu, D., Gong, F., Tang, H. & Zhang, Y. Identifying three ecological chemotypes of Xanthium strumarium glandular trichomes using a combined NMR and LC-MS method. *PLoS One* **8**, e76621, doi:10.1371/journal.pone.0076621 (2013).
7 Gutierrez, A. B., Oberti, J. C. & Herz, W. Germacran-5, 14, 6, 12-diolides from Mikania urticifolia. *Phytochemistry* **27**, 938-939 (1988).
8 Cuenca, M. D. R., Bardon, A., Catalan, C. A. & Kokke, W. Sesquiterpene lactones from Mikania micrantha. *Journal of natural products* **51**, 625-626 (1988).
9 Herz, W. & Govindan, S. V. The Germacradienolide Isabelin from Zexmenia valerii: Stereochemistry and conformation of scandenolide and deoxymikanolide. *Phytochemistry* **20**, 1740-1742 (1981).
10 But, P. P.-H., He, Z.-D., Ma, S.-C., Chan, Y.-M., Shaw, P.-C., Ye, W.-C. & Jiang, R.-W. Antiviral constituents against respiratory viruses from Mikania micrantha. *Journal of natural products* **72**, 925-928 (2009).
11 Aguinaldo, A. M., Abe, F., Yamauchi, T. & Padolina, W. G. Germacranolides of Mikania cordata. *Phytochemistry* **38**, 1441-1443 (1995).
12 Turner, M. & Brooks, P. Evaluation of the use of H2SO4-H2O2 digestions for the elemental analysis of plant tissue by ICP spectrometry. *Communications in soil science and plant analysis* **23**, 559-567 (1992).
13 Wu, Y., Ma, H. & Peng, Y. Effects of storage temperature and time on the contents of different nitrogen forms in fresh soil samples. *Ying yong sheng tai xue bao= The journal of applied ecology* **29**, 1999-2006 (2018).
14 Murphy, J. & Riley, J. P. A modified single solution method for the determination of phosphate in natural waters. *Analytica chimica acta* **27**, 31-36 (1962).
15 Harris, R. & Sommers, L. Plate-dilution frequency technique for assay of microbial ecology. *Appl. Environ. Microbiol.* **16**, 330-334 (1968).
16 Lin, X. Principles and methods of soil microbiology research. *High. Educ. Press, Beijing (In Chinese)* (2010).
