# Supplementary material for: Mikania micrantha genome provides insights into the molecular mechanism of rapid growth
Source: Nat Commun. 2020 Jan 17;11:340. doi: 10.1038/s41467-019-13926-4 (PMC6969026; doi:10.1038/s41467-019-13926-4)
Supplement: Supplementary file 3 — Reporting Summary [file 41467_2019_13926_MOESM3_ESM.pdf]

nature research

Corresponding author(s): DBPR, NCOMMS-19-16940A

Last updated by author(s): Nov 24, 2019

Reporting Summary

Nature Research wishes to improve the reproducibility of the work that we publish. This form provides structure for consistency and transparency in reporting. For further information on Nature Research policies, see Authors & Referees and the Editorial Policy Checklist.

Statistics

For all statistical analyses, confirm that the following items are present in the figure legend, table legend, main text, or Methods section.

n/a	Confirmed	
☐	☒	The exact sample size (*n*) for each experimental group/condition, given as a discrete number and unit of measurement
☐	☒	A statement on whether measurements were taken from distinct samples or whether the same sample was measured repeatedly
☐	☒	The statistical test(s) used AND whether they are one- or two-sided<br>*Only common tests should be described solely by name; describe more complex techniques in the Methods section.*
☒	☐	A description of all covariates tested
☐	☒	A description of any assumptions or corrections, such as tests of normality and adjustment for multiple comparisons
☐	☒	A full description of the statistical parameters including central tendency (e.g. means) or other basic estimates (e.g. regression coefficient) AND variation (e.g. standard deviation) or associated estimates of uncertainty (e.g. confidence intervals)
☐	☒	For null hypothesis testing, the test statistic (e.g. *F*, *t*, *r*) with confidence intervals, effect sizes, degrees of freedom and *P* value noted<br>*Give P values as exact values whenever suitable.*
☒	☐	For Bayesian analysis, information on the choice of priors and Markov chain Monte Carlo settings
☒	☐	For hierarchical and complex designs, identification of the appropriate level for tests and full reporting of outcomes
☐	☒	Estimates of effect sizes (e.g. Cohen's *d*, Pearson's *r*), indicating how they were calculated

Our web collection on statistics for biologists contains articles on many of the points above.

Software and code

Policy information about availability of computer code

Data collection	*Provide a description of all commercial, open source and custom code used to collect the data in this study, specifying the version used OR state that no software was used.*
Data analysis	clean_adapter and clean_lowqual (https://github.com/fanagislab/assembly_2ndGeneration/tree/master/clean_illumina)

For manuscripts utilizing custom algorithms or software that are central to the research but not yet described in published literature, software must be made available to editors/reviewers. We strongly encourage code deposition in a community repository (e.g. GitHub). See the Nature Research guidelines for submitting code & software for further information.

Data

Policy information about availability of data

All manuscripts must include a data availability statement. This statement should provide the following information, where applicable:

- Accession codes, unique identifiers, or web links for publicly available datasets
- A list of figures that have associated raw data
- A description of any restrictions on data availability

This Whole Genome project has been deposited at DDBJ/ENA/GenBank under the accession SZYD00000000. The version described in this paper is version SZYD01000000. For details, SRR8816384 and SRR8834228 - SRR8834566 for PacBio SMRT data have been deposited in SRA database; SRR8835135, SRR8835136 and SRR8835137 for the M. micrantha genomic Illumina DNA data; SRR8857616 - SRR8857640 for the M. micrantha RNA-seq data; SRR8834701-SRR8834748 for the M. micrantha full-length transcriptome data. For the defoliation experiment of M. micrantha, the RNA-seq data has been deposited as SRR8846782, SRR8846783, SRR8846784, SRR8846785, SRR8846786, SRR8846787. The metagenomics data has been deposited as SRR8936416 ~ SRR8936475.The M. micrantha genome assembly, gene prediction and functional annotation datas in this paper can also be accessed at ftp://ftp.agis.org.cn/Mikania_micrantha/.

Field-specific reporting

Please select the one below that is the best fit for your research. If you are not sure, read the appropriate sections before making your selection.

☒ Life sciences ☐ Behavioural & social sciences ☐ Ecological, evolutionary & environmental sciences

For a reference copy of the document with all sections, see nature.com/documents/nr-reporting-summary-flat.pdf

Life sciences study design

All studies must disclose on these points even when the disclosure is negative.

Sample size	No statistical methods were used to predetermine sample size. We collected a plant sample from Neilingding Island, Shenzhen, China; Based on the rarefaction analysis, the rarefaction analysis of 60 soil samples showed a curve approaching saturation
Data exclusions	No data was excluded in our analysis
Replication	photosynthesis experiment: 3 replications in CAM experiment and stem photosynthesis experiment. soil metagenome experiment: 5 replications.
Randomization	We randomly allocated the samples into experimental groups
Blinding	N/A

Reporting for specific materials, systems and methods

We require information from authors about some types of materials, experimental systems and methods used in many studies. Here, indicate whether each material, system or method listed is relevant to your study. If you are not sure if a list item applies to your research, read the appropriate section before selecting a response.

Materials & experimental systems

n/a	Involved in the study	
☒	☐	Antibodies
☒	☐	Eukaryotic cell lines
☒	☐	Palaeontology
☒	☐	Animals and other organisms
☒	☐	Human research participants
☒	☐	Clinical data

Methods

n/a	Involved in the study	
☒	☐	ChIP-seq
☒	☐	Flow cytometry
☒	☐	MRI-based neuroimaging
