# Supplementary material for: Mikania micrantha genome provides insights into the molecular mechanism of rapid growth
Source: Nat Commun. 2020 Jan 17;11:340. doi: 10.1038/s41467-019-13926-4 (PMC6969026; doi:10.1038/s41467-019-13926-4)
Supplement: Supplementary file 2 — Peer Review File [file 41467_2019_13926_MOESM2_ESM.pdf]

Reviewers' comments:

Reviewer #1 (Remarks to the Author):

Review
"Mikania micrantha, 'mile-a-minute', genome provides insights 1into the molecular mechanism of rapid growth"
Submitted by Liu et al.
Preliminary Remarks
I have focused on the sesquiterpene aspect of this work because this is my main expertise.
Comments
a) What are the major claims of the paper?
Liu et al. report the sequencing of the Mikania micrantha genome. Mikania micrantha is an invasive species from the Asteraceae family that causes major ecological and economic problems. They link their findings on the genome sequencing level to experiments aimed at photosynthesis mechanisms as well as sesquiterpene lactone production and its allelopathic effects. This combination of techniques is employed to understand the mechanism of rapid growth and "outperforming" other species in growth.
b) Are they novel and will they be of interest to others in the community and the wider field?
The findings are novel and will be interesting, not only to the community, but also to the wider field. There are several genome sequencing projects on Asteraceae plants. Most of these aim at agriculturally important crop plants such as sunflower or medically important plants such as Artemisia annua. To employ this technique for an invasive Asteraceae plant that causes damage to ecosystems and agriculture is generally a good idea and will be interesting to researchers from various communities. The role of photosynthesis, hormone regulation and sesquiterpene lactone composition on the competitiveness of the investigated species is, in this combination, a novel approach. The role of STL in allelopathy is convincing and it is an interesting approach to investigate how these allelopathic STL may be transferred into the soil. STL are generally cytotoxic and their main function is protection against herbivory, but also defense against microorganisms and allelopathy. All the STL detected here share the bioactive α-methylene-γ-lactone group that promotes bioactivity/cytotoxicity. It has also been shown how some STL induce in very low concentrations the germination of the root parasite Orobranche cumana (Joel et al. 2011, Raupp et al. 2013) and that some STL may play a role in physiological mechanisms of the plant (Yokotani-Tomita, 1997, 1999). What is rather surprising, however, is that the STL are supposed to have a stimulatory effect on the soil microbiome and are considered as a "new biofertilizer to promote nutrient cycling". This would indeed be a novelty, but the authors do not explain by what mechanism these STL are supposed to mediate this nutrient cycling effect. This part needs a more convincing explanation and more discussion.
c) Is the work convincing, and if not, what further evidence would be required to strengthen the conclusions?
The work is in most parts convincing and there is sufficient evidence to support the key findings. However, in some cases additional evidence is necessary, some passages that might be misleading have to be rewritten and additional data must be shown.

General considerations
1. Generally, the grammar and style should be checked in the main manuscript and the supporting material.

Part: "Metabolic pathway and genetic basis of Mikania sesquiterpene lactones"

2. l. 230: "Sesquiterpene lactones (STLs), which are mainly derived from the precursor germacrene A acid (GAA), are well known for their allelopathic effects and are characteristic of the family Asteraceae." => End sentence with a dot. Cite references for these statements.

3. l. 230: "The biosynthetic pathways of STLs related to synthetases including HaG8H, LsCOS, and Ih8H from sunflower, Lactuca sativa and Inula hupehensis have been elucidated 32-34" Like this the

sentence makes no sense. => keep references but rephrase: “Several enzymes acting downstream of GAA have been elucidated recently, such as HaH8H, LsCOS and Ih8H, that lead to the formation of different STL with distinctive stereochemistry32-34.”

4. l.234: “However, the biosynthetic pathways of STLs in Mikania remain unknown.” Here some information is missing: At this point the reader must be informed about the common stereochemistry of all STL detected in this study to understand why the authors chose to look for the Ih8H homolog. Include the following sentence: “As all the detected STL had a 7,8-trans lactone, we were investigating for a homolog of Ih8H, an enzyme capable of synthesizing 7,8-trans inunolide, the putative precursor of these Mikania micrantha STL.”

5. l.234: “Inunolide is a key intermediate of STL products, which can be identified and detected in fresh leaves by ultra-performance liquid chromatography—quadrupole time-of-flight—mass spectrometry (UPLC—QTOF—MS) (Supplementary Fig. S22)” Supp Figure S22 should contain a MS-MS spectrum of inunolide in the plant extract and ideally a run of a reference compound run in parallel. The authors should consider that STL from closely related species have been reported, that have the same exact mass and very similar MS-MS spectra and retention times. (costunolide, alantolactone, isoalantolactone, and other)

6. l. 244: “Based on the structural characteristics of the inunolide and STL products, the proposed downstream inunolide pathway of M. micrantha was demonstrated in this study (Figure 5c). Corresponding to the five genes interaction network, the candidate genes of STLs biosynthesis were detected, which may regulate the pathway producing STLs in M. micrantha (Figure 5a and Supplementary Table S8).” Several things have to be corrected here:
1. Fig 5a has a poor resolution and is as such meaningless, as the labeling can not be read.
2. Fig 5b: y-axis label has to be corrected.
3. Fig 5c: Eupatolide is a C12,6α lactone with a 8β-OH group. Here, the (incorrect) labeling is C12,8α.
4. Fig 5e: resolution and illustrative graphics must be improved.
5. Supplementary Table S8. The last nine genes have nothing to do with STL, why are they in this table? Other genes may be more interesting, like putative terpene transporters. Why are no expression values of trichomes reported? Have the P450 enzymes been assigned to a subfamily by David Nelson?
6. About the GAS, GAO, Ih8H homologs: the authors must provide the following: a) a percentage of amino acid identity of these homologous genes to the closest characterized gene (i.e. HaGAS, HaGAO, HaG8H or Ih8H). b) A phylogeny of the novel P450 candidates including LsCOS, HaG8H, HaES, Ih8H, HaGAO, and the respective homologs from Mikania or an amino acid alignment of the homologous genes c) for the GAO and Ih8H homologs as well as the P450 candidates for downstream STL pathway a systematic name (by D. Nelson).
7. Related to this: in supp material “3.4. The isolation of STLs from M. micrantha” the authors describe the isolation and NMR structure elucidation of the major STL from M. micrantha. However, the NMR data that prove the identity of these compounds are missing.

7. Generally, it is not clear why the authors did not investigate for the presence of trichomes on the leaf surface. There are microscopic pictures of the lower leaf showing stomata. In many plants in this tribe of the Asteraceae the STL are predominantly stored in trichomes. The authors have found the STL metabolites and biosynthesis genes in high abundance in the flowers and leaves. This may be because trichomes are found in flowers and in leaves. Trichomes are not mentioned once. The authors should check for the presence of trichomes on leaf and flower surfaces, and if they are present (what I assume) for their STL content. In this case I would recommend a LC-MS or HPLC run from isolated trichomes showing which STL are derived from there. Also, microscopic pictures of trichomes and gene expression studies of STL synthesizing genes in trichomes would make sense here. (See also: Paes de Almeida et al. 2017 Comparative morphoanatomical analysis of Mikania species)

d) On a more subjective note, do you feel that the paper will influence thinking in the field?
The paper will influence thinking in the field (see also part b)

Conclusion
The manuscript should be accepted, but only with major revisions (see also part b and c).

Reviewer #2 (Remarks to the Author):

Liu et al. report a chromosome scale genome of the rapid growing plant Mikania micrantha and analyze genomic features associated with its high photosynthetic rate and fast growth. Gene duplicates retained from its whole genome and segmental duplications are enriched in functions related to growth and photosynthesis, reflecting adaptive evolution to fast growth. The authors provide several lines of evidence supporting CAM photosynthesis activity in M. micrantha, and when combined with stem photosynthesis, may help boost growth. The authors also identified candidate biosynthesis genes for the allelochemicals STLs and propose a model where STLs promote soil nitrogen cycling. I have some minor concerns that should be addressed to strengthen this manuscript.

1. Line 118. The authors state the sharp Ks peak < 0.2 correlates to recent segmental duplications that span ~23% of the M. micrantha genome. Based on the Kmer peak in Supplemental Figure 2, it seems like this genome is highly heterozygotic. These duplicated regions may represent haplotype specific sequences that were not filtered out using falcon-unzip from the assembly.
The amount of haplotype-specific sequence identified and filtered out of the assembly is not discussed in the methods or the results, but this would be useful to provide in a supplemental table.

2. Plant genomes contain multiple copies of core CAM pathway genes, and most are involved in housekeeping processes and metabolic functions unrelated to CAM. The genes shown in the heatmap of figure 3b may not be the copies functioning in CAM. For instance, PEPC is usually expressed and transcribed during the day time in CAM plants, and post-translationally activated through phosphorylation by PEPCK at night. The three PEPC genes shown in Figure 3b have high nocturnal but low diurnal expression, contrasting what has been shown in other CAM lineages. A single subfamily of PEPC has been recruited independently in all C4 and CAM lineages and a phylogenetic tree of PEPC genes may help the authors resolve which copies are functioning in CAM.
Based on the results, it seems like only two timepoints were collected for surveying CAM gene expression, but six are shown in the heatmap in Figure 3b. Is this heatmap just showing independent replicates?

Reviewer #3 (Remarks to the Author):

This manuscript deals with the genome of M. micrantha, an invasive species of the Asteraceae family that is characterized by a very rapid growth. The authors describe the assembly and annotation, they perform a comparative genomics study with other Asteraceae species, and date the expansion of LTR-retrotransposons. Then they investigate the genomic basis of rapid growth and production of allelochemicals in M. micrantha.

The manuscript is generally well written, although some sentences are long and some jargon is used (generalist readers may not know what "allelochemical" means). The main text nevertheless lacks some basic information on some methods (e.g., a network is built but it is not clear in the text whether this is a metabolic or co-expression network), or there is a reference to a control without

explicit stating of what is this control. There is also a lack to the references to the proper sections of the methods or supplementary material (e.g. L235-236, there is no indication as where the methods that were used to build the network are described).

The genome assembly and annotation metrics are good, but unless I am wrong, the authors did not provide accession number for their release (from the detailed description of data availability only for raw data, which is not enough). An increasing number of plant genome are "published" without actually releasing the genome assembly, which I find really concerning, because the ressource are not really "provided" to the community.

Concerning the general genome analysis performed in the manuscript, the comparative genomics analysis in Astereaceae shows that M. micrantha, which belongs to the Heliantheae tribe, shares a whole-genome-duplication with sunflower. However, this not a scoop, the results presented here are very similar to previous findings (Badouin et al 2017, but also Barker et al 2008, who already shown that the whole-genome duplication of sunflower occurred at the basis the Heliantheae tribe). The analysis of LTR-retrotransposons is rather superficial and does not bring very interesting information.

The investigation of the genomic bases of specific life-history traits is more interesting. The authors find an enrichment of photosynthesis-related genes, but I am concerned that such genes are often left out in genome assemblies and that the enrichment seen here may be very dependent on methods that were used to assemble the different genomes in the dataset. The investigation of allelochemicals production is very interesting, especially because of the soil metagenomic experiments, but I must admit that I am not a specialist of soil biology and metabolism and that I lack the skills to evaluate this part. The defoliating experiment is also interesting but the manuscript should state if defoliated growth is common in M. micrantha, otherwise one does not see the revelance of the experiment.

Below are some more specific comments or questions to improve the manuscript:

Abstract
L31-32: not clear, what allelochemicals are is not clear for a generalist reader.
L33-34: the findings above can explain the rapid growth, but if one does not know what "allelochemicals" means one does not see how it relates to the invasiveness of the species.

Introduction
-paragraph
L42: add a comma to cut this long sentence "to the canopy, and blocking the sunlight"
-paragraph2: very clear

-paragraph3:
L57: of "the" invasive species: suppress "the"
L61: use past tense ("we generateD" ... "and performED")
L63: use plural: "genetic processes and molecular mechanisms"

Results
Section 1 LTR-RT
-paragraph1 assembly
how did the authors validate the correspondance between heterozygote fragments. How was genome size evaluated? What is the mean length of subreads?

-Paragraph 2 annotation:
L85-86: not very informative, the different "functional" databases provide very different levels of evidence. What databases were used?

Paragraph 3 LTR

L89: "high coverage and continuity": no information on how many gaps remain. The "continuous" aspect is not well accounted for.

L90-91: "which play multiple role...": this part of the sentence is not informative. Be more precise or remove.

L96-99: no information on the method that was used to estimated the age of LTR-RT expansion.

Section2 WGD: problem with the title "improve ecological adaptation" (no evidence for that). Please tone down.
L108-111: missing R in "orthologous"
I do not understand the difference between the two parts of the sentence, separated by 'as well'. I do not understand what "syntenic genes" are: synteny is studied at the level of multiple genes.
L117: "with Ks values less than 0.2": Do you mean "lower than 0.2"?
Sentence not clear "the enrichment of 127 synteny blocks": enrichment in what?

L123-127: sentence too long that is difficult to read, cut it in to.

-Section 2 metabolism. Again, a very affirmative title. Is the level of evidence sufficient?
Paragraph 2
L152-155: the important information is whether all genes in the CAM pathway are present in the genome, not the raw number of candidate genes. Is this the case, or are there missing genes?

Paragraph 3
L160: diel?

L179-L181: tone down the conclusion, this is suggested but not the definitive evidence.

Section 4: paragraph 1:
L188-L189: why this choice of plants. Are they particularly representative?

paragraph 2: defoliating experiment
L211-212: "on the basis of contrasted expression pattern": uncessary piece of sentence.

Section allelopathic effects:
L230-231: again, define allelopathic for the generalist reader.
L231: point at the end of the sentence, not comma.
L235: "genome-scale network": do you mean a metabolic network? Co-expression network? This must be stated.

L243: Define LC-MS?
L244: of five genes: of the five genes
L244-246: sentence not clear. What do you mean by "was demonstrated in this study"?
L246-248: not clear either. What is the "five genes interaction network?"
Overall, paragraph 2 of this section needs to be re-written in a clearer way, removing redundancy and highlighting the conclusions

L276: a, not "an" at the end of the sentence.
L279: use "increased", not "improved"
What is proof that the increased CO_2 content comes from the microorganisms and not from the plants? Why add the STLs to soil near a monoculture rather than to bare soil alone? It is not clear in the text whether the five STLs were added together or separately.

paragraph 2:

L285-287: what is the control?
Overall, the interest of this experiment should be better justified.

paragraph soil metagenomic
L302-305 sentence not correct, delete "the abundance" (no ok with "is significantly enriched")
L319-320: not clear, what do you mean by "able to prevent the loss of available nitrogen".
L322: "was all" > were
L322: "these evidences" > these evidence (no S)

All this message on the effect of STL in interesting, but there is no evidence of the allelopathic effect of these STL. Relevant with the message?

Discussion:

L327-330: not informative
L330-332: WGD not a scoop, already known that it was shared by most Heliantheae.
L333-334: redundant with the results section.
L334: double T
L335 with "a" published genome
L336: upper case in Asterids
L336-337: how?
L344-L345: "most than ..." only if the set of other species chosen in the stem photosynthesis experiment was representative. It is not possible to judge it because this choice was not justified.
L348-349: what do you mean by "transferring enzymes from C4 to C3 plants"?
L352-353: this sentence has no verb. remove the ", which "?
L356: "could not only promoted" > "could not only promote". What is dissimilatory?
L360-364: is this biosynthesis pathway for STL different than in other plants? If not it is not "new".

Figure 1:
L423: missing space
2c: at the scale it is very difficult to visualize the synteny relationships between the two genomes. Zoom on several interesting chromosomes and put the complete dotplot in supplementary?

Figure 3:
a) not obvious from this table than CAM genes are enriched.
e) L443: add "proposed pathway", be careful on the conclusions.

Responses to comments from Reviewer 1

What is rather surprising, however, is that the STL are supposed to have a stimulatory effect on the soil microbiome and are considered as a “new biofertilizer to promote nutrient cycling”. This would indeed be a novelty, but the authors do not explain by what mechanism these STL are supposed to mediate this nutrient cycling effect. This part needs a more convincing explanation and more discussion.

In previous researches, allelochemicals were mainly reported to inhibit microorganisms. However, some studies also showed that allelochemicals can stimulate soil microfauna and microorganisms. For example, the study on a serious invasive species, *Fallopia japonica,* which is greatly threating to Europe, found that the abundance of soil predator Acari could be increased by adding rhizome extract of *Fallopia japonica* (Abgrall et al., 2018). Chen et al. (2009) added the extract of *Mikania micrantha* to bare soil, and found that the content of NH_4^+-N and NO_3^--N significantly increased. These studies have shown that allelochemicals can stimulate soil microorganisms. However, similar effects of STL on soil microorganisms have not been reported yet.

According to the reviewer’s comments, we performed two experiments to find more evidences that STLs promote nutrient cycling of soil.

Experiment 1:

Five STL (Dih, Anh, Deo, Sca, 3-epi) were adding to the bare soil to identify whether STL affects microorganisms and promote nutrient cycling. The concentration of NH_4^+-N was increased when adding STL to the soil (Supplementary Table 11). Then, we selected the Dih soil, which was the highest among the five STL in the soil colonized by *Mikania micrantha*, for high-throughput sequencing. The 16S sequencing results showed that the relative abundance of Bradyrhizobium was significantly increased after adding Dih

compound at genus levels (P-value = 0.002, two-sided t-tests, see Supplementary Figure 35). Bradyrhizobium belong to the nitrogen fixation bacteria and convert molecular nitrogen in the air into ammonia or related nitrogenous compounds in soil.

Experiment 2:

The density of cultured nitrogen-fixing bacteria was used to prove the effects of STL on microorganisms. Adding 1mL of 2 mg/L STLs (Dih, 3-epi, and mixture of five STL) and 0.5 mL cultured nitrogen-fixing bacteria respectively to 25mL of culture medium. Meantime, adding STL (Dih, 3-epi, and mixture of five STL) respectively to culture medium, and adding nothing as the control. These samples were cultured in a shaker at 28℃ and 150 rpm for 7 days. Each treatment was replicated 5 times. After then, OD values of each treatment were measured at 600 wavelengths by ultraviolet spectrophotometer (UV-2450) to evaluated the density of azotobacter. We found that the colony-forming units (CFU) for nitrogen-fixing bacteria were significantly increased after adding STL to the culture medium (**Figure a**). These results showed that the STLs promote nitrogen-fixing bacteria growth.

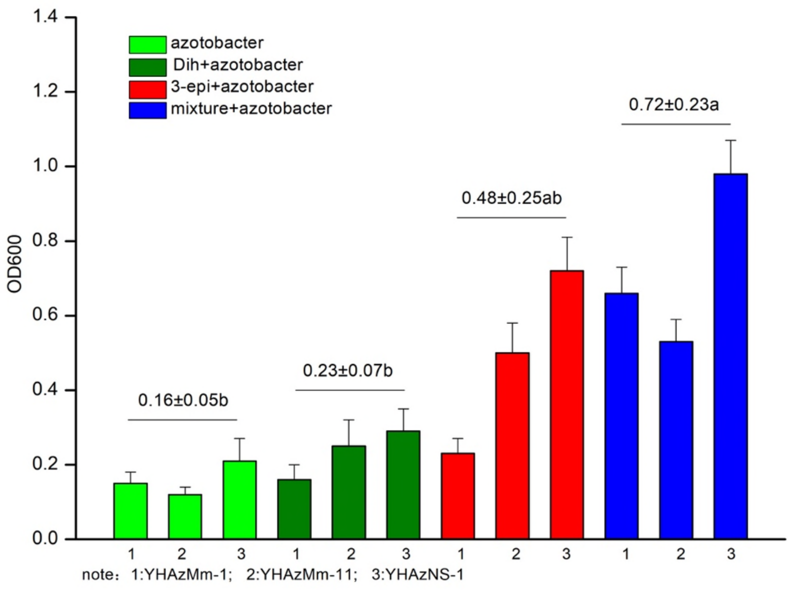

Figure a. Effect of STL on the density of azotobacter (AV±SD, n=5).

According to these results, STLs can increase concentration of NH_4^+-N and promote nitrogen-fixing bacteria growth. However, we cannot explain the molecular mechanism of STL's influence on soil microorganisms exactly. Not to give the reader ambiguous conclusions, we changed the title of this section from “Mikania sesquiterpene lactones promote nutrient utilization by reinforcing the soil microbial functioning” to “Reinforced soil microbial functioning promotes its nutrient utilization”. We modified the conclusion that STL promotes soil microbial enrichment and soil nutrient cycling, including Figure 6a, Supplementary figure 24 and Supplementary Table 9. And deleted this sentence “STL may be applied to develop a new biofertilizer to promote nutrient cycling in important crop soils” in discussion part. In addition, we modified the Figure 6.

References:

1 Abgrall, C., Forey, E., Mignot, L. & Chauvat, M. Invasion by Fallopia japonica alters soil food webs through secondary metabolites. Soil Biology and Biochemistry 127, 100-109, doi:10.1016/j.soilbio.2018.09.016 (2018).

2 Chen, B., Peng, S. & Ni, G. Effects of the invasive plant Mikania micrantha H.B.K. on soil nitrogen availability through allelopathy in South China. Biological Invasions 11, 1291-1299 (2009).

General considerations

1. Generally, the grammar and style should be checked in the main manuscript and the supporting material.

We agree with the reviewer's comment. The grammar and style have been revised in main manuscript and supporting material.

Part: "Metabolic pathway and genetic basis of *Mikania* sesquiterpene lactones"

2. l. 230: "Sesquiterpene lactones (STLs), which are mainly derived from the precursor germacrene A acid (GAA), are well known for their allelopathic effects and are characteristic of the family Asteraceae." => End sentence with a dot. Cite references for these statements.

We have cited references for these statements in revised version.

3. l. 230: "The biosynthetic pathways of STLs related to synthetases including HaG8H, LsCOS, and Ih8H from sunflower, Lactuca sativa and Inula hupehensis have been elucidated 32-34" Like this the sentence makes no sense. => keep references but rephrase: "Several enzymes acting downstream of GAA have been elucidated recently, such as HaH8H, LsCOS and Ih8H, that lead to the formation of different STL with distinctive stereochemistry32-34."

We agree with the reviewer's comment. The sentence has been revised.

4. l.234: “However, the biosynthetic pathways of STLs in *Mikania* remain unknown.” Here some information is missing: At this point the reader must be informed about the common stereochemistry of all STL detected in this study to understand why the authors chose to look for the Ih8H homolog. Include the following sentence: “As all the detected STL had a 7,8-trans lactone, we were investigating for a homolog of Ih8H, an enzyme capable of synthesizing 7,8-trans inunolide, the putative precursor of these Mikania micrantha STL.”

This part has been revised to “However, the biosynthetic pathways of STLs in Mikania remain unknown. Lactone ring formation in STLs is dependent upon the (C6 or C8) position and stereoselective hydroxylations of GAA, which can result in STLs with four unique configurations (12, 6α-, 12, 6β-, 12, 8α-, and 12, 8β-olides derivatives of GAA) (Gou et al., 2017). Ih8H homolog is important enzyme convert to 7, 8-trans lactone from GAA. As all the detected STL in this study had a 7,8-trans lactone, we were investigating for a homolog of Ih8H, an enzyme capable of synthesizing 7, 8-trans inunolide, the putative precursor of these *Mikania micrantha* STL.”

References:

1 Gou, J. et al. Discovery of a non-stereoselective cytochrome P450 catalyzing either 8α- or 8β-hydroxylation of germacrene A acid from the Chinese medicinal plant, Inula hupehensis. 93 (2017).

5. l.234: “Inunolide is a key intermediate of STL products, which can be identified and detected in fresh leaves by ultra-performance liquid chromatography—quadrupole time-of-flight—mass spectrometry (UPLC—QTOF—MS) (Supplementary Fig. S22)” Supp Figure S22 should contain a MS-MS spectrum of inunolide in the plant extract and ideally a run of a reference compound run in parallel. The authors should consider that STL from closely related species have been reported, that have the same exact mass and very similar MS-MS spectra and retention times. (costunolide, alantolactone, isoalantolactone, and other)

Because inunolide standard is not available from commerce. We tried to isolated

inunolide from *M. micrantha*, however, the content of this compound was low. The ^{1}H NMR spectrum of isolated inunolide was obtained as show in **Figure b**.

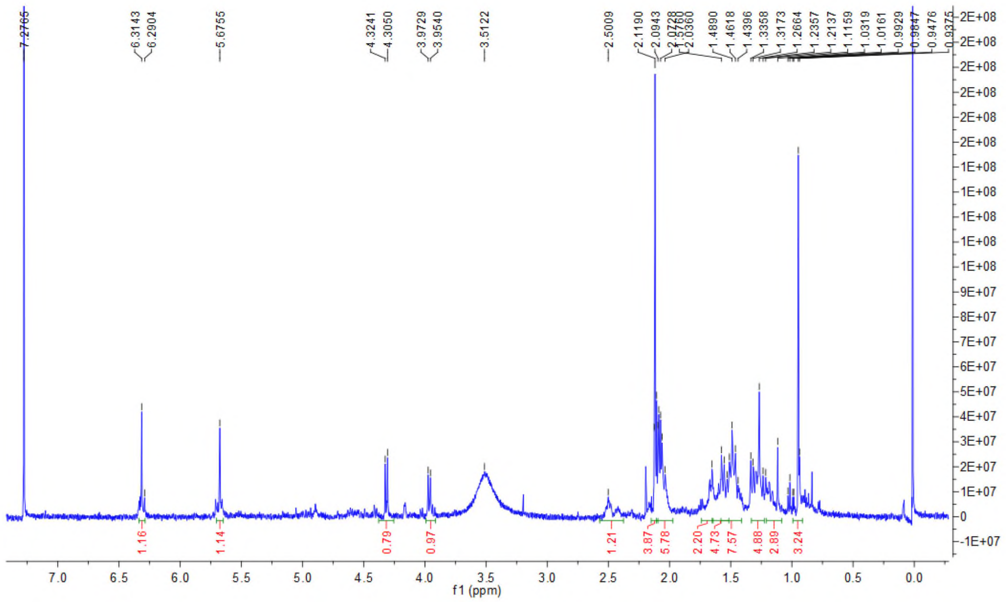

Figure b. ^{1}H NMR of inunolide isolated from M. micrantha leaves.

The NMR chemical shift of isolated compound were closely similar to those of inunolide (Gou et al., 2017), which identified as inunolide. We also analyzed the isolated inunolide and isomer compound (costunolide standard). Both of them displayed a different retention time but similar fragments (**Figure c**). Moreover, constunolide cannot be detected in different tissues of *M. micrantha*. We have revised Supplementary Figure 22, which contain a MS-MS spectrum of inunolide and costunolide standard.

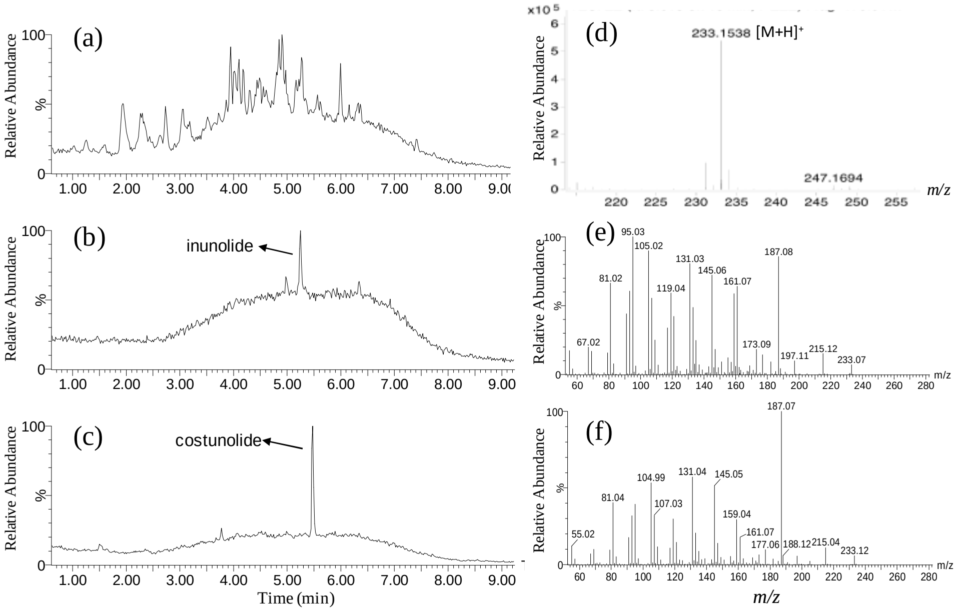

Figure c. LC-MS analyzed chloroform dipped extract of *M. micrantha* leaves, inunolide and costunolide standard in parallel (positive mode). (a) Total ion chromatograph (TIC) of chloroform dipped extract of leaves; (b) TIC of innunolide (Rt 5.25 min) isolated from M. micrantha; (c) TIC of costunolide standard (Rt 5.48 min); (d) The high resolution mass spectrum of inunolide in positive ion, the molecular formula $C_{15}H_{20}O_2$ from its molecular ions at m/z 233.1538[M+H]$^+$ (calcd for $C_{15}H_{21}O_2$, 233.1542); (e)-(f) MSMS spectra of inunolide and costunolide (collision energy was set at 20 eV).

Inunolide isomers including alantolactone, isoalantolactone and costunolide were also analyzed by LC-MS in parallel. As shown in **Figure d**, they present the same extract mass (extract mass at *m/z* 233.15) but different retention times, indicating that the isolated compound (m/z 233.1538) from *M. micrantha* should be inunolide.

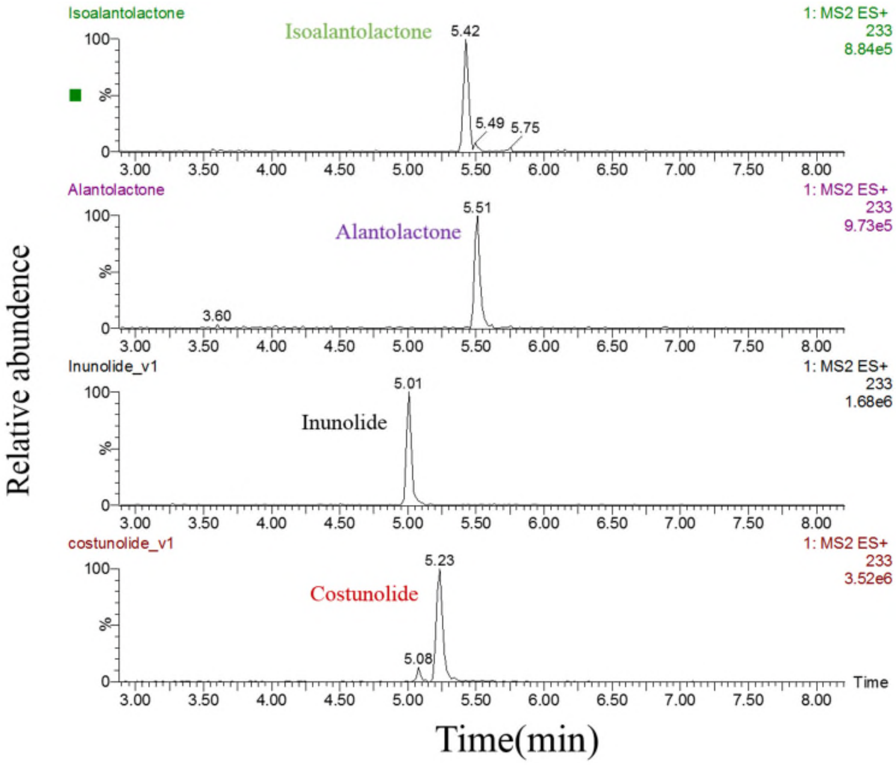

Figure d. Comparative analysis of the isolated inunolide and its isomers by LC-MS at positive mode (extract m/z at 233.15). Alantolactone, isoalantolactone and costunolide were standard compounds.

Reference:

Gou, J. et al. Discovery of a non-stereoselective cytochrome P450 catalyzing either 8α- or 8β-hydroxylation of germacrene A acid from the Chinese medicinal plant, Inula hupehensis. 93 (2017).

6. l. 244: "Based on the structural characteristics of the inunolide and STL products, the proposed downstream inunolide pathway of *M. micrantha* was demonstrated in this study (Figure 5c). Corresponding to the five genes interaction network, the candidate genes of STLs biosynthesis were detected, which may regulate the pathway producing STLs in *M. micrantha* (Figure 5a and Supplementary Table S8)." Several things have to be corrected

here:

1. Fig 5a has a poor resolution and is as such meaningless, as the labeling can not be read.

The resolution of Figure 5a have modified in the revised manuscript.

2. Fig 5b: y-axis label has to be corrected.

We used the standard curve of inunolide to calculate the content of inunolide in fresh tissues. Thus, y-axis label of Figure 5b have been corrected.

3. Fig 5c: Eupatolide is a C12,6α lactone with a 8β-OH group. Here, the (incorrect) labeling is C12,8α.

Eupatolide is a C12,6α lactone, which have been changed in Figure 5c of revised manuscript.

4. Fig 5e: resolution and illustrative graphics must be improved.

We have corrected those in the revised manuscript.

5. Supplementary Table S8. The last nine genes have nothing to do with STL, why are they in this table? Other genes may be more interesting, like putative terpene transporters. Why are no expression values of trichomes reported? Have the P450 enzymes been assigned to a subfamily by David Nelson?

We agreed with the reviewer's comments. We deleted the last nine candidate genes in supplementary Table S8.

Because glandular trichomes of *M. micrantha* are only observed under light microscope, it is very difficult to isolate them from the leaves or flowers. So, we did not detect the gene expressions in trichomes.

According to the David Nelson research, we found the P450 enzymes were assigned to one subfamily CYP71.

6. About the GAS, GAO, Ih8H homologs: the authors must provide the following: a) a percentage of amino acid identity of these homologous genes to the closest characterized gene (i.e. HaGAS, HaGAO, HaG8H or Ih8H). b) A phylogeny of the novel P450 candidates including LsCOS, HaG8H, HaES, Ih8H, HaGAO, and the respective homologs from Mikania or an amino acid alignment of the homologous genes c) for the GAO and Ih8H homologs as well as the P450 candidates for downstream STL pathway a systematic name (by D. Nelson).

a) It is good suggestion for identifying homologous genes. We downloaded the GAS, GAO Ih8H protein sequences in *Helianthus annuus, Artemisia annua, Lactuca sativa* and *Inula hupehensis*, respectively. a) Based on BLASTP analysis, the candidate genes in *Mikania* showed high identify and coverage with *Helianthus annuus, Artemisia annua, Lactuca sativa* and *Inula hupehensis* (**Table a**). And we have added this results in revised version.

Table a The comparison of GAS, GAO, Ih8H homologous in *M. micrantha, Helianthus annuus, Artemisia annua, Lactuca sativa* and *Inula hupehensis*.

**Gene Name of *M. micrantha***	**Gene Name of Relative Species**	**Species**	**Identity (%)**	**Gene Coverage**
MmGAS1 (Mm17G037281)	HaGAS1 (XP_022010071.1)	*Helianthus annuus*	87	99.6

MmGAS2 (Mm17G037281)	HaGAS2 (XP_022015687.1)	*Helianthus annuus*	86	99.6
MmGAS3 (Mm17G037281)	HaGAS3 (ACZ50512.1)	*Helianthus annuus*	82	99.5
MmGAS (Mm17G037281)	AaGAS (ABE03980.1)	*Artemisia annua*	76	97.3
MmGAO1 (MmUnG044903)	LsGAO (ADF32078.1)	*Lactuca sativa*	75	100%
MmGAO1 (MmUnG044903)	HaGAO (ADF43082.1)	*Helianthus annuus*	79	100%
MmGAO2 (MmUnG044904)	LsGAO (ADF32078.1)	*Lactuca sativa*	72	100%
MmGAO2 (MmUnG044904)	HaGAO (ADF43082.1)	*Helianthus annuus*	73	100%
MmGAO3 (MmUnG044907)	LsGAO (ADF32078.1)	*Lactuca sativa*	75	100%
MmGAO3 (MmUnG044907)	HaGAO (ADF43082.1)	*Helianthus annuus*	78	100%
MmIh8H (Mm07G019019)	HaG8H (XP_022035380.1)	*Helianthus annuus*	61	98.7
MmIh8H (Mm07G019019)	Ih8H (AML23863.1)	*Inula hupehensis*	49	99.6

b) the phylogenetic tree showed that the candidate genes of GAS, GAO and Ih8H in *M. micrantha* were on the same clade with its homologous genes (**Figure e**). And we have added this results in revised version.

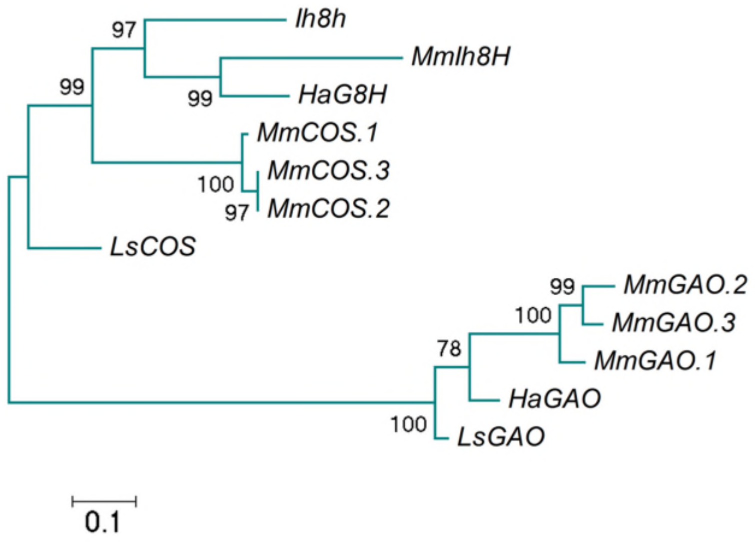

Figure e. Phylogenetic tree of GAS, GAO, Ih8H homologous in *M. micrantha, Helianthus annuus, Artemisia annua, Lactuca sativa* and *Inula hupehensis.*

c) We have systematically named the P450 candidates for downstream STL pathway by D. Nelson.

7. Related to this: in supp material "3.4. The isolation of STLs from *M. micrantha*" the authors describe the isolation and NMR structure elucidation of the major STL from *M. micrantha*. However, the NMR data that prove the identity of these compounds are missing.

We isolated and identified five STLs in *M. micrantha*, the purity was analyzed by HPLC and ^{1}H NMR (**Figure f**).

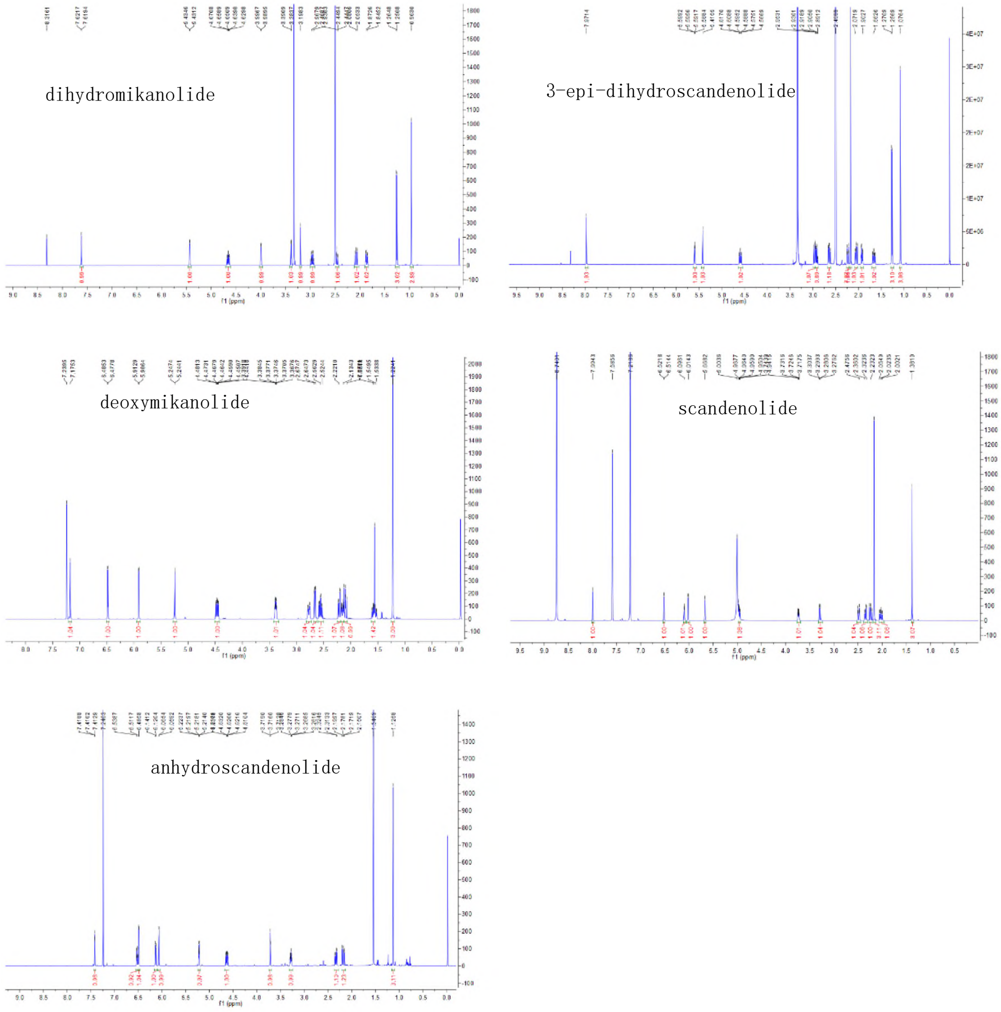

Figure f. ^{1}H NMR spectrum of five isolated STLs from *M. micrantha*.

The spectroscopic data of five known STLs as bellowing, which have matched the data of previous reports.

Anhydroscandenolide (1): white crystals, ESI-MS m/z 273.05 $[M - H]^-$; ^{1}H NMR (500 MHz, $CDCl_3$) δ: 3.28 (1H, dq, J = 4.7, 3.4 Hz, H-1), 6.52 (1H, ddd, J = 10.4, 3.1, 1.9 Hz, H-2a), 6.13 (1H, d, J = 10.4 Hz, H-3), 7.42 (1H, m, H-5), 5.22 (1H, dt, J = 4.8, 1.9 Hz, H-6), 3.72 (1H, t, J = 1.5 Hz, H-7), 4.63 (1H, ddd, J = 10.8, 8.1, 5.6 Hz, H-8), 2.18 (1H, dd, J = 13.8, 10.7 Hz, H-9a), 2.33 (1H, dd, J = 14.0, 5.6 Hz, H-9b), 6.49 (1H, d, J = 3.5 Hz, H-13a), 6.06 (1H, d, J = 3.1 Hz, H-13b), 1.13 (3H, s, Me-14). ^{13}C NMR (126 MHz, $CDCl_3$) δ: 59.2 (C-1), 123.8 (C-2), 135.0 (C-3), 131.1 (C-4), 151.0 (C-5), 82.2 (C-6), 49.5 (C-7),

76.7 (C-8), 43.8 (C-9), 58.7 (C-10), 136.9 (C-11), 167.7 (C-12), 125.1 (C-13), 22.5 (C-14), 170.3 (C-15). (Gutierrez et al., 1988; Cuenca et al., 1988)

Deoxymikanolide (2): colerless crystals, ESI-MS m/z 275.06 $[M - H]^-$; ^1H NMR (500 MHz, $CDCl_3$) δ: 2.77 (1H, dd, J = 13.3, 5.9 Hz, H-1), 1.56 (1H, m, H-2a), 2.56 (1H, td, J = 12.9, 6.3 Hz, H-2b), 3.38 (1H, dtd, J = 4.8, 3.5, 1.4 Hz, H-3a), 2.15 (1H, m, H-3b), 7.18 (1H, s, H-5), 5.25 (1H, d, J = 1.7 Hz, H-6), 2.66 (1H, dd, J = 11.4, 2.3 Hz, H-7), 4.47 (1H, ddd, J = 11.2, 8.6, 4.5 Hz, H-8), 2.10 (1H, dd, J = 14.2, 11.2 Hz, H-9a), 2.21 (1H, dd, J = 14.3, 4.5 Hz, H-9b), 6.48 (1H, d, J = 3.7 Hz, H-13a), 5.91 (1H, d, J = 3.2 Hz, H-13b), 1.22 (3H, s, Me-14). (Herz et al., 1981; But et al., 2009)

Dihydromikanolide (3): colorless granular crystal, ESI-MS m/z 291.06 $[M - H]^-$； ^1H NMR (500 MHz, DMSO) δ: 3.20 (1H, s, H-1), 3.39 (1H, dd, J = 3.6, 1.0 Hz, H-2), 3.99 (1H, d, J = 3.6 Hz, H-3), 7.62 (1H, d, J = 1.2 Hz, H-5), 5.43 (1H, d, J = 1.7 Hz, H-6), 2.46 (1H, m, H-7), 4.65 (1H, td, J = 10.6, 4.6 Hz, H-8), 2.08 (1H, dd, J = 13.6, 10.8 Hz, H-9a), 1.86 (1H, dd, J = 13.7, 4.6 Hz, H-9b), 2.96 (1H, dd, J = 11.8, 7.0 Hz, H-11), 1.26 (3H, d, J = 7.0 Hz, Me-13), 0.96 (3H, s, Me-14). ^{13}C NMR (126 MHz, DMSO) δ: 57.3 (C-1), 54.7 (C-2), 52.1 (C-3), 128.6 (C-4), 149.9 (C-5), 81.7 (C-6), 50.2 (C-7), 76.6 (C-8), 42.1 (C-9), 57.4 (C-10), 41.0 (C-11), 176.0 (C-12), 13.2 (C-13), 20.8 (C-14), 171.0 (C-15). (Herz et al., 1981; Cuenca et al., 1988).

Scandenolide (4): white crystals, ESI-MS m/z 333.10 $[M - H]^-$; ^1H NMR (500 MHz, Pyridine-d_5) δ: 3.29 (1H, dd, J = 11.5, 2.2 Hz, H-1), 2.02 (1H, m, H-2a), 2.49 (1H, dt, J = 14.9, 2.1 Hz, H-2b), 6.10 (1H, br d, J = 3.8 Hz, H-3), 7.99 (H, s, H-5), 5.67 (1H, br d, J = 1.6 Hz, H-6), 3.73 (1H, dd, J = 8.4, 1.2 Hz, H-7), 4.96 (1H, m, H-8), 2.23 (1H, dd, J = 13.9, 11.2 Hz, H-9a), 2.34 (1H, dd, J = 14.0, 4.3 Hz, H-9b), 6.52 (1H, d, J = 3.7 Hz, H-13a), 6.02 (1H, d, J = 3.3 Hz, H-13b), 1.38 (3H, s, Me-14), 2.17 (3H, s, 3-OAc). ^{13}C NMR (126 MHz, Pyridine-d_5) δ: 59.1 (C-1), 30.1 (C-2), 67.9 (C-3), 133.5 (C-4), 148.2 (C-5), 83.2 (C-6), 50.7 (C-7), 78.8 (C-8), 43.8 (C-9), 57.6 (C-10), 137.9 (C-11), 168.4 (C-12), 123.1 (C-13), 21.1 (C-14), 170.6 (C-15), 169.9 (3-OAc), 20.6 (3-OAc). (Aguinaldo et al., 1995).

3-epi-Dihydroscandenolide (5): colorless crystal, ESI-MS m/z 335.12 $[M - H]^-$; ^1H NMR (500 MHz, DMSO) δ: 2.96 (1H, dd, J = 11.6, 2.1 Hz, H-1), 2.23 (1H, dt, J = 14.8, 2.0 Hz, H-2a), 2.64 (1H, dd, J = 12.6, 9.8 Hz, H-2b), 5.59 (1H, br d, J = 3.7 Hz, H-3), 7.97 (1H, br s, H-5), 5.42 (1H, s, H-6), 1.66 (1H, ddd, J = 14.9, 11.6, 4.4 Hz, H-7), 4.59 (1H, dt, J = 10.9, 4.1 Hz, H-8), 1.92 (1H, dd, J = 14.1, 4.1 Hz, H-9a), 2.05 (1H, dd, J = 14.0, 11.1 Hz, H-9b), 2.92 (1H, dt, J = 13.9, 6.9 Hz, H-11), 1.26 (3H, d, J = 7.0 Hz, Me-13), 1.08 (3H, s, Me-14), 2.17 (3H, s, 3-OAc). (But et al., 2009).

All the data have added into supporting material "3.4. The isolation of STLs from *M. micrantha*".

References:

1 Aguinaldo, A. M., Abe, F. T. & Padolina, W. G. J. P. GERMACRANOLIDES OF MIKANIA CORDATA. 38, 1441-1443 (1995).

2 But, P. P. et al. Antiviral constituents against respiratory viruses from Mikania micrantha. J Nat Prod 72, 925-928, doi:10.1021/np800542t (2009).

3 Cuenca Mdel, R., Bardon, A., Catalan, C. A. & Kokke, W. C. Sesquiterpene Lactones from Mikania micrantha. J Nat Prod 51, 625-626, doi:10.1021/np50057a040 (1988).

4 Gutierrez, A. B., Oberti, J. C. & Herz, W. J. P. Germacran-5,14,6,12-diolides from Mikania urticifolia. 27, 938-939 (1988).

5 Herz, W. & Govindan, S. V. J. P. The germacradienolide isabelin from Zexmenia valerii: stereochemistry and conformation of scandenolide and deoxymikanolide. 20, 1740-1742 (1981)..

7. Generally, it is not clear why the authors did not investigate for the presence of trichomes on the leaf surface. There are microscopic pictures of the lower leaf showing stomata. In many plants in this tribe of the Asteraceae the STL are predominantly stored

in trichomes. The authors have found the STL metabolites and biosynthesis genes in high abundance in the flowers and leaves. This may be because trichomes are found in flowers and in leaves. Trichomes are not mentioned once. The authors should check for the presence of trichomes on leaf and flower surfaces, and if they are present (what I assume) for their STL content. In this case I would recommend a LC-MS or HPLC run from isolated trichomes showing which STL are derived from there. Also, microscopic pictures of trichomes and gene expression studies of STL synthesizing genes in trichomes would make sense here. (See also: Paes de Almeida et al. 2017 Comparative morphoanatomical analysis of Mikania species)

Thanks for the comments. We observed the flower, leaf and stem of *M. micrantha* under light microscope. As show in **Figure g**, the glandular trichomes were abundant in the bract of dry flower (**Figure g**A-B). While there two type of trichomes (conical glandular trichome and uniseriate glandular trichome) were observed on leaf surface. And most of those were conical glandular trichome (**Figure g**D). Less trichomes were found in surface view of stem, most of them were non-glandular trichome. This finding was similar to previous report that trichome index of leaf are high than that of stem (**Figure h**, Sathi et al., 2015). However, we found that the flower (bract) also contain a large number of glandular trichome. This may has resulted that STLs metabolites in high abundance in the flowers and leaves.

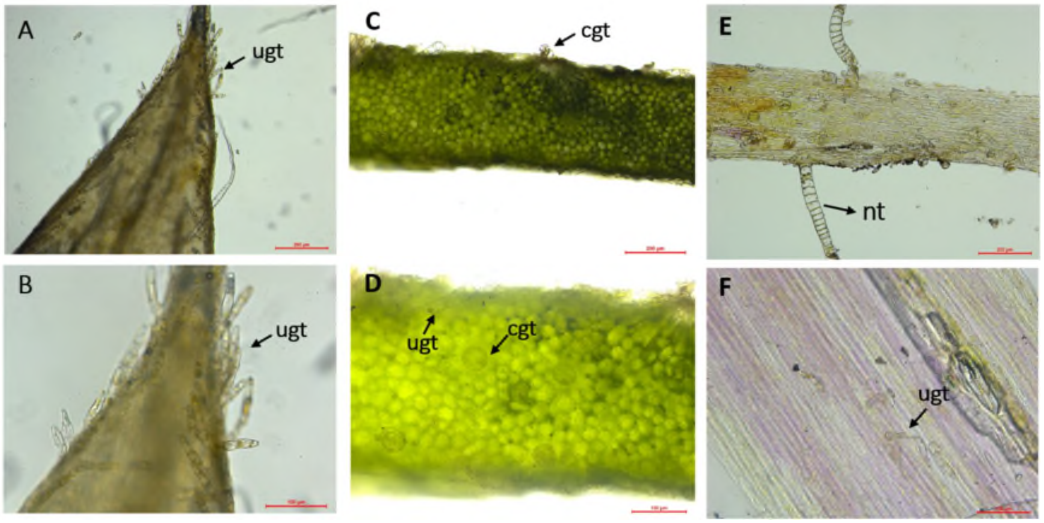

Figure g. Trichome in different part of *M. micrantha.* A-B: The bract of flower in surface view. C-D: Surface view of leaf. E-F. Surface view of stem. ugt: uniseriate glandular trichome; cgt: conical glandular trichome; nt: non-glandular trichome. 200 µm (A, C, E); 100 µm (B, D, F).

Table 3: Trichome features of *M. micrantha**

Trichome types	Occurrence	Length (µm)	Width (µm)	Diameter (µm)	No. of cells / trichome	Trichome Index (%)
Non-glandular	Petiole & Stem	338.32	21.36	-	6 - 17	-
	Leaf	83.33	19.7	-	3 - 4	0.13 (upper) 0.02 (lower)
Multicellular, uniseriate, glandular	Petiole & Stem	51.1	16.43	-	2 - 3	-
	Leaf	64.04	14.97	-	3 - 4	0.95 (upper) 1.22 (lower)
Conical, stalked glandular	Leaf	41.98	37.23	128.93	-	0.75 (upper) 0.59 (lower)

*Data presented in the table are averages of 20 observations.

Figure h. Trichome index in different tissues of *M. micrantha* (Sathi, 2015)

Because glandular trichomes of *M. micrantha* are only observed under light microscope, it is very difficult to isolate the glandular trichomes from the leaves or flowers. Previous studies showed that the most of STLs in young leaves of the seedlings obtained were dipped in chloroform for 30 s, which extracts truly reflected the chemical characteriastics inside glandular trichomes (Majdi et al., 2011, Chen et al., 2013).

According to the method, we extracted STLs in *M. micrantha* leaf. The results showed that it can extract most of STLs in the leaf trichomes (**Figure i**). Less STLs can be detected after chloroform dipping extract with 30 s. For example, the relative content of deoxymikanolide compound in chloroform dipping extracts present peak area at 8292, while the residue extracts only have relative peak area at 73 (**Figure i**). These evidences indicated the STL compounds were major distributed in glandular trichome of *M. micrantha*.

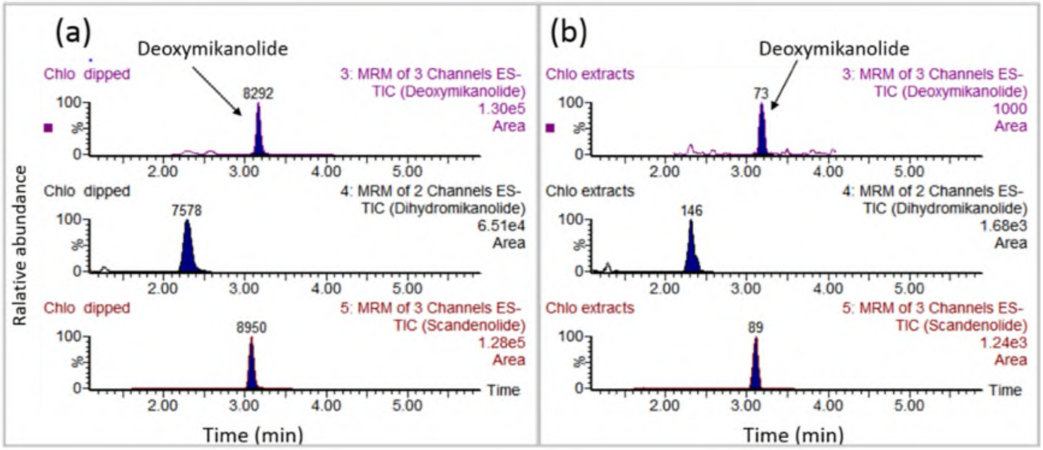

Figure i. Comparative analysis of chloroform dipping extract and the residue extracts of fresh *M. micrantha* leaves. A: MRM of scandenolide, dihydromikanolide and deoxymikanolide from chloroform dipped extracts. B: MRM analyzed the STLs from the residue extract by chlorolform.

Five STLs (Anh, Deo, Dih, Sca, 3-epi) in different parts of glandular trichomes were also quantified by LC-MS/MS analysis. As shown in **Figure j**, the content of five STLs in flowers and leaves trichomes were similar and high abundance, but low content in the stem trichomes. This result was supported by **Figure i** in manuscript that the STLs genes and STLs metabolites were high express and content in the leaf and flower extract of *M. micrantha*. Thus, it was confirmed that STLs mainly came from the glandular trichomes of *M. micrantha.*

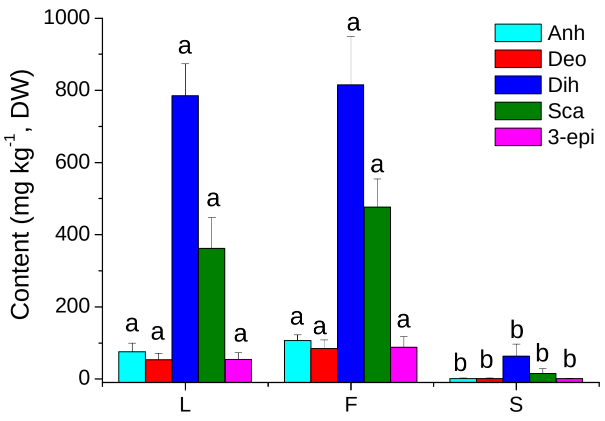

Figure j. Quantification of five STLs in different dried tissues of trichomes of *M. micrantha* (L: leaf, F: flower; S: stem).

References:

1 Chen, F. et al. Identifying three ecological chemotypes of Xanthium strumarium glandular trichomes using a combined NMR and LC-MS method. Plos ONE, 8, e76621 (2013).

2 Majdi, M. et al. Biosynthesis and localization of parthenolide in glandular trichomes of feverfew (Tanacetum parthenium L. Schulz Bip.). Phytochemistry 72, 1739-1750 (2011).

3 Saha, S., Mandal, S. K., Chowdhury, H. R. J. I. J. o. R. i. A. & Pharmacy. Anato-pharmacognostic studies of mikania micrantha kunth: A promising medicinal climber of the family asteraceae. International Journal of Research in Ayurveda & Pharmacy. 6, 773-780 (2015).

Responses to comments from Reviewer 2

1. Line 118. The authors state the sharp Ks peak < 0.2 correlates to recent segmental duplications that span ~23% of the M. micrantha genome. Based on the Kmer peak in

Supplemental Figure 2, it seems like this genome is highly heterozygotic. These duplicated regions may represent haplotype specific sequences that were not filtered out using falcon-unzip from the assembly.

The amount of haplotype-specific sequence identified and filtered out of the assembly is not discussed in the methods or the results, but this would be useful to provide in a supplemental table.

We agree with the reviewer's comment. We added the methods of genome assembly and haplotype identification in supplemental Note 1. "First, the PacBio reads were used to assemble the *M. micrantha* genome by the software canu-1.6, and 7,523 contigs were generated with a total length of 2.16 Gb. Based on the distribution of k-mer frequencies, the estimated genome size of M. micrantha is 1.87 Gb. Then, we used the following methods to filter the heterozygous sequences: Firstly, we used the genome sequence to align to itself by MUMmer3.23, and filtered out the heterozygous segments with more than 70% coverage and 70% identity. Secondly, the PacBio reads were re-assembled by falcon/falcon-unzip, which generated 1,684 Mb primary reference sequence and 352 Mb alternative heterozygous haplotype sequence (haplotig). Then, we aligned the 352 Mb alternative heterozygous haplotype sequence (haplotig) to the canu assembled contigs, to further filter the heterozygous sequences in the canu reference sequences. The resulting 4,414 contigs with a total length of 1.79 Gb were used as the final haploid reference genome of *M.micrantha*".

2. Plant genomes contain multiple copies of core CAM pathway genes, and most are involved in housekeeping processes and metabolic functions unrelated to CAM. The genes shown in the heatmap of figure 3b may not be the copies functioning in CAM. For instance, PEPC is usually expressed and transcribed during the day time in CAM plants, and post-translationally activated through phosphorylation by PEPCK at night. The three PEPC genes shown in Figure 3b have high nocturnal but low diurnal expression, contrasting what has been shown in other CAM lineages. A single subfamily of PEPC has been recruited independently in all C4 and CAM lineages and a phylogenetic tree of

PEPC genes may help the authors resolve which copies are functioning in CAM.

We agree with the reviewer's comment that most of multiple copies of core CAM pathway genes are involved in housekeeping processes and metabolic functions unrelated to CAM. And there was also reports that PEPC was activated by phosphorylation to catalyse the primary assimilation of CO2 in Crassulacean acid metabolism plants at night. The expression patterns of three PEPC genes shown in Figure 3b were really inconsistent with other CAM lineages, so these three PEPC genes shown in Figure 3b maybe not functioning in CAM. So according to reviewer's suggestion, we identify the PEPC genes of CAM.

Four published CAM plant genomes (pineapple (Ming et al., 2015), *Phalaenopsis equestris* (Cai et al., 2015), *Apostasia shenzhenica* (Zhang et al., 2017), *Dendrobium officinale* (Zhang et al., 2016)) were chosen to distinguished PEPC genes functioning in CAM in *M. micrantha*. It was reported that there are three putative PEPC genes in pineapple. As the pineapple genome is a model for studying the evolution of CAM photosynthesis, the three PEPC genes in pineapple were used to identify the homologous genes in *P. equestris, A. shenzhenica, D. officinale* and *M. micrantha* using BLASTP.

The three PEPC genes in pineapple were used to align to the protein sequences with e-value cutoff of 1×10^{-5} in *P. equestris, A. shenzhenica, D. officinale* and *M. micrantha*, respectively. Based on BLASTP analysis, totally eleven candidate PEPC genes were identified in *P. equestris* (2), *A. shenzhenica* (2), *D. officinale* (2) and *M. micrantha* genome (5) (identify > 60% and coverage > 60%). The phylogenetic tree showed the relationships of the putative PEPC genes in these CAM plants (**Figure k**). In phylogenetic tree, all the putative PEPC genes were clustered into two clades. Two *M. micrantha* genes Mm10G023910, Mm08G019821 and one pineapple gene Aco016429.1 belong to one clade, while three *M. micrantha* genes Mm07G016791, Mm12G028694, Mm11G0125286, and the other putative PEPC genes belong to another clade. Based on the diurnal expression pattern, only Mm07G016791 has high diurnal and low nocturnal expression. So Mm07G016791 was candidate PEPC gene of *M. micrantha*. The

expression of the PEPC genes (Mm07G016791) was showed in Figure 3b in the revised manuscript.

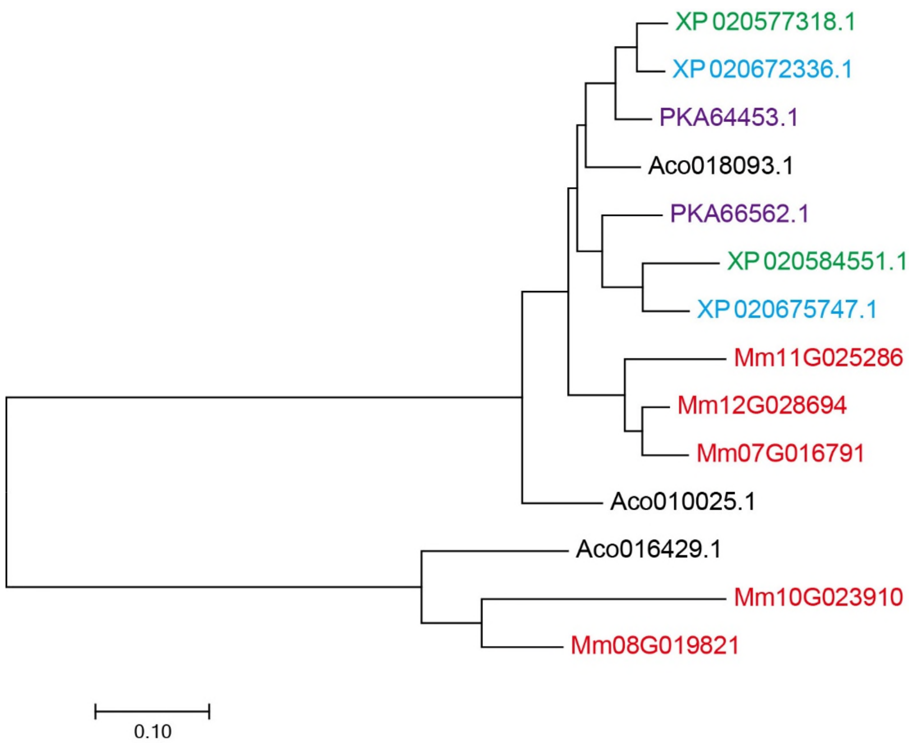

Figure k. Phylogenetic tree of PEPC genes in pineapple, *P. equestris, A. shenzhenica, D. officinale* and *M. micrantha*. Different colors represent different species: green for *P. equestris*; blue for *D. officinale*; purple for *A. shenzhenica*; black for pineapple; red for *M. micrantha*.

References:

1 Cai, J. et al. The genome sequence of the orchid Phalaenopsis equestris. Nat Genet 47, 65-72, doi:10.1038/ng.3149 (2015).

2 Zhang, G. Q. et al. The Apostasia genome and the evolution of orchids. Nature 549, 379-383, doi:10.1038/nature23897 (2017).

3 Zhang, G. Q. et al. The Dendrobium catenatum Lindl. genome sequence provides insights into polysaccharide synthase, floral development and adaptive evolution. Sci Rep 6, 19029, doi:10.1038/srep19029 (2016).

4 Ming, R. et al. The pineapple genome and the evolution of CAM photosynthesis. Nat Genet 47, 1435-1442, doi:10.1038/ng.3435 (2015).

Based on the results, it seems like only two timepoints were collected for surveying CAM gene expression, but six are shown in the heatmap in Figure 3b. Is this heatmap just showing independent replicates?

Figure 3b shows the biological replicates at two time points (9 a.m. and 9 p.m.). We have added the sentence “The gene expression of each replicate sample in two time points (9 a.m. and 9 p.m.) presented in the heatmap.” in the figure legend in the revised manuscript.

Responses to comments from Reviewer 3

The manuscript is generally well written, although some sentences are long and some jargon is used (generalist readers may not know what "allelochemical" means).

Thanks for the reviewer’s suggestion. The defines of Allelochemical and allelopathic have been added in the introduction part.

The main text nevertheless lacks some basic information on some methods (e.g., a network is built but it is not clear in the text whether this is a metabolic or co-expression network),

We constructed a co-expression network to speculate STL proposed biosynthesis pathway by transcriptome data. We have made corresponding modifications in the main text.

or there is a reference to a control without explicit stating of what is this control.

We have explained it in the revised version.

There is also a lack to the references to the proper sections of the methods or supplementary material (e.g. L235-236, there is no indication as where the methods that were used to build the network are described).

We changed the sentence from "We reconstructed a genome-scale network for *M. micrantha* and focused on the metabolic pathways involved in inunolide synthesis" to "We reconstructed a genome-scale co-expression network for *M. micrantha* and focused on the metabolic pathways involved in inunolide synthesis". In the transcriptome data analysis of materials and methods part, we described the method of co-expression network analysis. "Co-expression network analysis was performed by WGCNA package using all transcriptomes. WGCNA network construction and module detection was identified using an unsigned type of topological overlap matrix (TOM) with a soft-thresholding power β of 28 ($R^2 > 0.9$)".

The genome assembly and annotation metrics are good, but unless I am wrong, the authors did not provide accession number for their release (from the detailed description of data availability only for raw data, which is not enough). An increasing number of plant genome are "published" without actually releasing the genome assembly, which I find really concerning, because the resource are not really "provided" to the community.

We agree with the reviewer's suggestion. When the manuscript is submitted, the genome sequence and gene annotation results had been uploaded to NCBI. We have shown the accession number in the maintext.

The defoliating experiment is also interesting but the manuscript should state if defoliated growth is common in *M. micrantha*, otherwise one does not see the revelance of the experiment.

Defoliation is a common phenomenon in the whole life history of plants, such as insect

bite, drought and other conditions can lead to plant leaf wilting, litter and so on. In this study, the stem photosynthetic capacity of *Mikania micrantha*, other vines and associated plants under normal conditions was measured. It was found that the stem of *Mikania micrantha* has a certain photosynthetic capacity, which is much higher than that of the control plant. In order to further study whether the stem photosynthetic capacity of *Mikania micrantha* can independently maintain the growth of plants, the related stem physiological indicators were determined by removing the interference of leaf factors. Finally, combined with the analysis of physiology and transcriptome, it was found that the stem of *Mikania micrantha* had a certain photosynthetic compensation effect when it lost its leaves, which could independently maintain the normal growth of plants (**Figure I** and **Supplementary Figure S16**). This study indicated that *Mikania micrantha* could cope with adversity through non-photosynthetic organ stems. It provided favorable evidence to ensure that *Mikania micrantha* could continue to survive under adversity when photosynthetic organ leaves wither or even die.

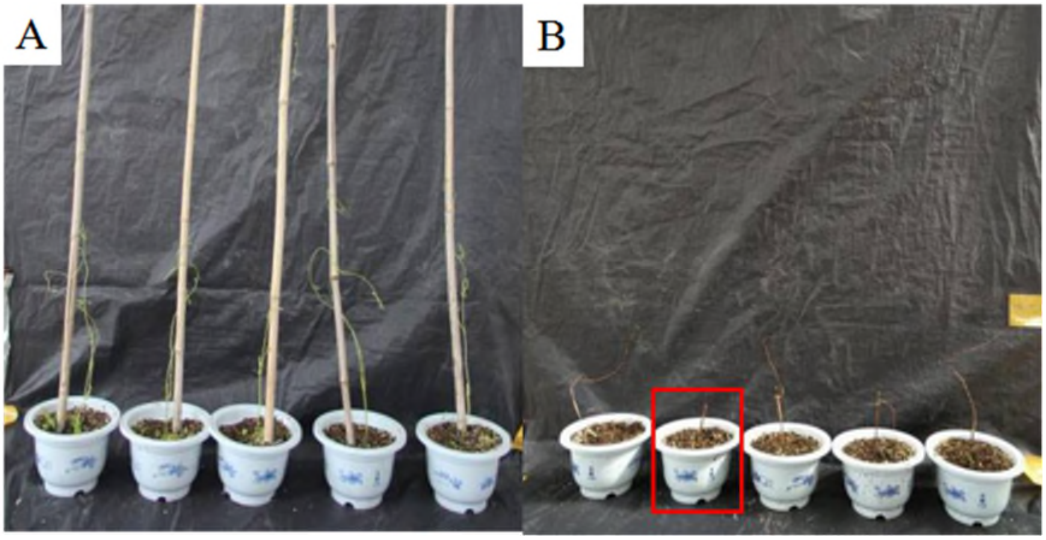

Figure I. Growth situation of the two plants after treatment

(A. *M. micrantha*; B. *P. scandens*; The dead plant was signed in red)

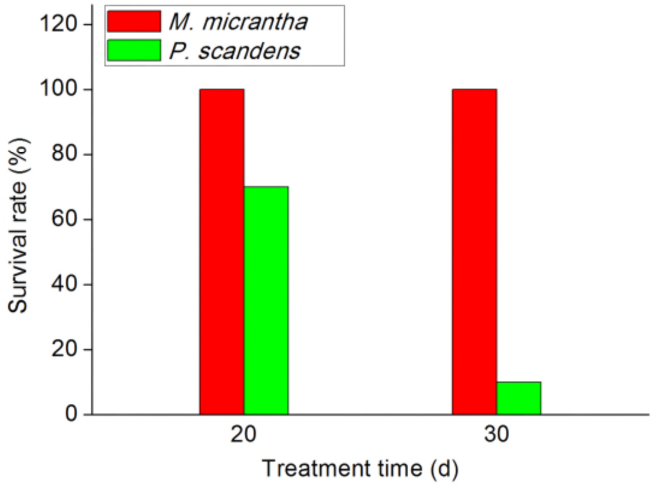

Supplementary Figure 16. The survival rates of the *M. micrantha* and *P. scandens* under the defoliation experiment.

Abstract

L31-32: not clear, what allelochemicals are is not clear for a generalist reader.

We have added the define of allelochemicals in the introduction part.

"Allelopathy is the influence of a plant over a target species, through the release into the environment of compounds that influence the growth and development of biological systems (Rice et al., 1983). Various allelochemicals, that are produced by a plant must escape into the environment and subsequently influence the growth and development of other plants (Einhellig et al., 2018)"

References:

1 Einhellig, F. A. in Handbook of natural pesticides: methods 161-200 (CRC Press, 2018).

2 Rice, E. L. Allelopathy. (Academic Press, 2013)

L33-34: the findings above can explain the rapid growth, but if one does not know what "allelochemicals" means one does not see how it relates to the invasiveness of the species.

We have added the define of allelochemicals in the introduction part.

Introduction

-paragraph

L42: add a comma to cut this long sentence "to the canopy, and blocking the sunlight"

We have added a comma to cut this sentence.

-paragraph3:

L57: of "the" invasive species: suppress "the"

We have delete “the” in this sentence in revised version.

L61: use past tense ("we generateD" ... "and performED")

According to the comment, we have corrected the sentence in revised version.

L63: use plural: "genetic processes and molecular mechanisms"

According to the comment, we have corrected the sentence in revised version.

Results

Section 1 LTR-RT

-paragraph1 assembly

how did the authors validate the correspondance between heterozygote fragments. How was genome size evaluated? What is the mean length of subreads?

For the heterozygote fragments, we added the methods of genome assembly and haplotype identification in supplemental Note 1. “First, the PacBio reads were used to

assemble the M. micrantha genome by the software canu-1.6, and 7,523 contigs were generated with a total length of 2.16 Gb. Based on the distribution of k-mer frequencies, the estimated genome size of M. micrantha is 1.87 Gb. Then, we used the following methods to filter the heterozygous sequences: Firstly, we used the genome sequence to align to itself by MUMmer3.23, and filtered out the heterozygous segments with more than 70% coverage and 70% identity. Secondly, the PacBio reads were re-assembled by falcon/falcon-unzip, which generated 1,684 Mb primary reference sequence and 352 Mb alternative heterozygous haplotype sequence (haplotig). Then, we aligned the 352 Mb alternative heterozygous haplotype sequence (haplotig) to the canu assembled contigs, to further filter the heterozygous sequences in the canu reference sequences. The resulting 4,414 contigs with a total length of 1.79 Gb were used as the final haploid reference genome of *M. micrantha*".

We estimated the size of genome based on k-mer frequencies in Illumina reads. The K-mer frequencies along the sequence depth (Liu et al., 2013). During deduction, the genome size G = K_num/K_depth, where the K_num is the total number of K-mer, and K_depth is the frequency occurring more frequently than the others. This part has been added to supplementary Note 1.

Subread: Each polymerase read is partitioned to form one or more subreads, which contain sequence from a single pass of a polymerase on a single strand of an insert within a SMRTbell™ template and no adapter sequences. The subreads contain the full set of quality values and kinetic measurements.

Reference:

Binghang Liu, Yujian Shi, Jianying Yuan, et al. and Wei Fan*. Estimation of genomic characteristics by analyzing k-mer frequency in de novo genome project. arXiv.org arXiv: 1308.2012. (2013)

-Paragraph 2 annotation:

L85-86: not very informative, the different "functional" databases provide very different levels of evidence. What databases were used?

We agree with the reviewer’s comment. The sentence has been change from “A total of 42,804 (92%) coding proteins were annotated by functional databases (Supplementary Table S5)” to “A total of 42,804 (92%) coding proteins were annotated by functional databases (Supplementary Table S5), including eggNOG, KEGG, Interpro and uniprot”.

Paragraph 3 LTR

L89: "high coverage and continuity": no information on how many gaps remain. The "continuous" aspect is not well accounted for.

There are only 1,599 (0.0089% of whole genome) gaps in M. micrantha reference genome sequences. This result has been added in Table 1 in revised version.

L90-91: "which play multiple role...": this part of the sentence is not informative. Be more precise or remove.

We remove this sentence “which play multiple roles in driving genome evolution in eukaryotes” in the revised version.

L96-99: no information on the method that was used to estimated the age of LTR-RT expansion.

We have added this sentence “The LTR insert time is estimated by LTR-retriever.” to describe the method of LTR expansion time estimation in Materials and Methods.

Section2 WGD: problem with the title "improve ecological adaptation" (no evidence for that). Please tone down.

We agreed with reviewer’s comment. The title has been change to “Genome-wide replication and subsequent fragment replication of *Mikania micrantha* may play an important role in ecological adaptation”.

L108-111: missing R in "orthologous"

We have revised this error.

I do not understand the difference between the two parts of the sentence, separated by 'as well'. I do not understand what "syntenic genes" are: synteny is studied at the level of multiple genes.

The sentence has been changed to “To study the conservation of genomic structure, we identified the inter-species syntenic genes between *M. micrantha* and sunflower, artichoke, and grape, and intra-species syntenic genes within the *M. micrantha*, sunflower, lettuce, artichoke and coffee genomes, then calculated the Ks values of the syntenic fragments for orthologous pairs”.

L117: "with Ks values less than 0.2": Do you mean "lower than 0.2"?

Yes, we changed this sentence from “less than 0.2” to “lower than 0.2”.

Sentence not clear "the enrichment of 127 synteny blocks": enrichment in what?

We changed this sentence from “we identified the enrichment of 127 syntenic blocks” to “we identified 127 syntenic blocks”.

L123-127: sentence too long that is difficult to read, cut it in to.

This sentence has been changed to “The WGT-1, WGD-2 and SD-3 duplicated genes were significantly enriched in the functions of catalytic and hydrolase activity, nitrogen compound metabolism, transcription factor, response to stimulus and chemical (Figure 2d and Supplementary Figure 9-10). It demonstrated that extensive gene fractionation has occurred during the evolutionary history of the *M. micrantha* genome, which retained the genes that are essential for survival and lost other redundant genes.”

-Section 2 metabolism. Again, a very affirmative title. Is the level of evidence sufficient?

We think it's a good suggestion. The title has been changed from “Cooperation of the C3 and CAM photosynthesis pathways provides abundant carbohydrates for rapid growth” to “High photosynthesis of the leaves provides abundant carbohydrates for rapid growth”.

Paragraph 2

L152-155: the important information is whether all genes in the CAM pathway are present in the genome, not the raw number of candidate genes. Is this the case, or are there missing genes?

All key genes in the CAM and C4 pathway were identified in *Mikania micrantha* genome (see **Figure m**).

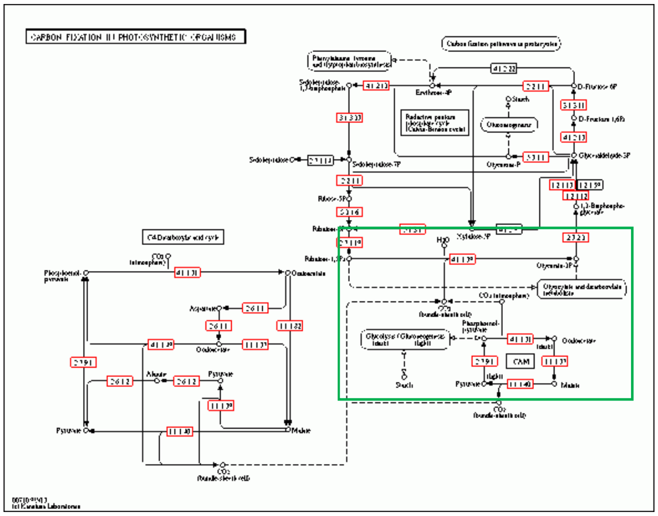

Figure m. The photosynthetic KEGG pathway in *Mikania micrantha*. The red boxes indicate that the genes of pathway were identified in *M. micrantha* genome. The green box represented the CAM pathway.

Paragraph 3

L160: diel?

The sentence has been changed from “To investigate the diel enzyme activity patterns of the CAM pathway” to “To investigate the enzyme activity patterns of the CAM pathway in day and night”.

L179-L181: tone down the conclusion, this is suggested but not the definitive evidence.

The sentence has been changed from “Together, these results showed that *M. micrantha* can not only fix carbon dioxide by the C3 pathway in the daytime, but also obtain sufficient carbon dioxide though the CAM pathway at night.” to “These results indicate that one of the reasons for the higher photosynthetic capacity of *Mikania micrantha* leaves is that CO2 may be absorbed at night by photosynthetic pathway similar to CAM and stored in vacuoles as organic acids to supplement the carbon fixation capacity during the day”.

In addition, we added some discussion in revised version (see discussion part).

Section 4: paragraph 1:

L188-L189: why this choice of plants. Are they particularly representative?

Thank you very much for careful reading and your valuable comment. We chose these species because that the five species, including *Mikania micrantha, Paederia scandens, Pueraria lobata, Stephania longa* and *Ipomoea cairica*, were similarities in morphological characteristics. They were vines and lived in the same habits. Although *Merremia hederacea* was a kind of herb, its leaf shape was similar to that of *M. micrantha*.

paragraph 2: defoliating experiment

L211-212: "on the basis of contrasted expression pattern": unnecessary piece of sentence.

We have removed this sentence “on the basis of contrasted expression pattern” from the revised version.

Section allelopathic effects:

L230-231: again, define allelopathic for the generalist reader.

We have added the define of allelopathic in the Introduction part. “allelopathic effects is a biological phenomenon by which an organism produces one or more biochemicals that influence the growth, survival, and reproduction of other organisms.”

L231: point at the end of the sentence, not comma.

This sentence has been revised.

L235: "genome-scale network": do you mean a metabolic network? Co-expression network? This must be stated.

"genome-scale network" means Co-expression network. We changed the sentence from “We reconstructed a genome-scale network for *M. micrantha*” to “We reconstructed a co-expression network for *M. micrantha*”.

L243: Define LC-MS?

We changed the sentence from “we have developed an LC-MS method to determine the relative inunolide contents” to “we have developed a liquid chromatograph-mass spectrometer (LC-MS) method to determine the relative inunolide contents”.

L244: of five genes: of the five genes

This sentence has been revised.

L244-246: sentence not clear. What do you mean by "was demonstrated in this study"?

We have revised this sentence to: “This suggests that inunolide is likely as

intermediate to the STLs metabolites in *Mikania,* thus, the proposed pathway of STLs in *M. micrantha* was displayed in green arrows (Figure 5c in maintext)."

L246-248: not clear either. What is the "five genes interaction network?"

This sentence has been revised from "Corresponding to the five genes interaction network, the candidate genes of STLs biosynthesis were detected" to "According to the co-expression network, the candidate genes of STLs biosynthesis were detected".

Overall, paragraph 2 of this section needs to be re-written in a clearer way, removing redundancy and highlighting the conclusions

Thanks for reviewer's suggestion. We rewrote the paragraph to make it easier to understand.

This paragraph has been change into "STLs have been postulated to be derived from the precursor germacrene A acid (GAA). Several enzymes acting downstream of GAA have been elucidated recently, such as HaH8H, LsCOS and Ih8H, that lead to the formation of different STL with distinctive stereochemistry. However, the biosynthetic pathways of STLs in Mikania remain unknown. Formation of the lactone rings depends on the (C6 or C8) position and stereoselective (α- or β-) hydroxylations of GAA, resulting in four possible region and stereo-specific configurations (12, 6α-, 12, 6β-, 12, 8α-, and 12, 8β-olides). Phytochemicals investigation on M. micrantha have isolated and identified STLs had a 7, 8-trans lactone (12, 8 α-olide derivatives), we were investigating for a homolog of Ih8H, an enzyme capable of synthesizing 7, 8-trans inunolide, the putative precursor of these *Mikania micrantha* STL.".

L276: a, not "an" at the end of the sentence.

It has been revised.

L279: use "increased", not "improved"

It has been revised.

What is proof that the increased CO_2 content comes from the microorganisms and not from the plants?

We collected the soil in the field, then sifted the soil through a sieve with 1mm diameter. Then, we add the STLs into the soil without any plants, and put it in a incubator. So absolutely the increased CO_2 content comes from the microorganism and not from the plants.

Why add the STLs to soil near a monoculture rather than to bare soil alone?

We think the soil near Mikania monoculture will be the nearest colony for Mikania in the near future during the period of the plant Mikania spreading. If the STLs could be the new weapon for Mikania colonizing, the soil near the monoculture would be influenced first.

The soil near the monoculture is the same as the bare soil because there are no plants growing in the soil near the Mikania monoculture. The picture we collected the soil is as follows:

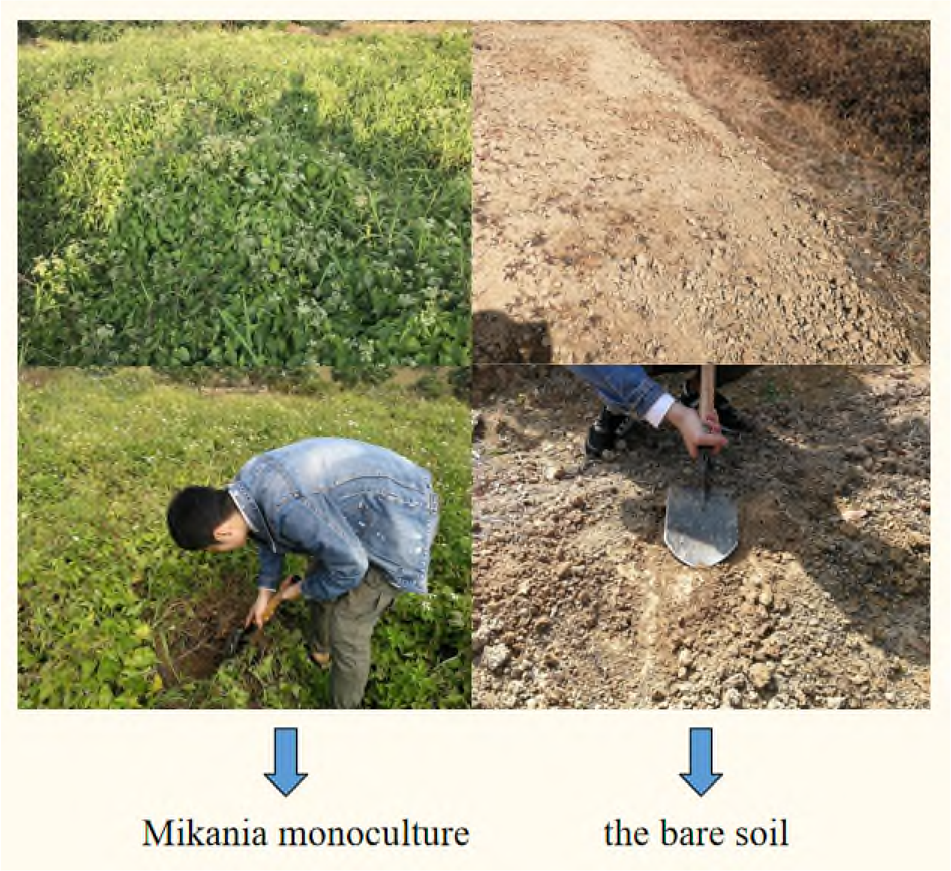

It is not clear in the text whether the five STLs were added together or separately.

We try to understand how STLs have an effect on the soil in the process of Mikania invading, and which one affected the most, so we added the five STLs into the soil separately, not together. We added this information in the text.

paragraph 2:

L285-287: what is the control?

Overall, the interest of this experiment should be better justified.

The control is the blank soil which no plants were planted. This sentence has been changed from “compared to that in the soil of its' two neighboring species and the

control” to “compared to that in the soil of its’ two neighboring species and the control which no plants were planted”.

paragraph soil metagenomic

L302-305 sentence not correct, delete "the abundance" (no ok with "is significantly enriched")

We agreed with the reviewer’s comment. We deleted the "the abundance of " in revised version.

L319-320: not clear, what do you mean by "able to prevent the loss of available nitrogen".

Because denitrifying bacteria are responsible for converting nitrate nitrogen into gaseous nitrogen oxides and consuming them, a reduction in denitrification genes means a reduction in the loss of available nitrate nitrogen.

We changed the sentence from “suggesting that the soil microbes of *M. micrantha* were able to prevent the loss of available nitrogen.” to “suggesting that the soil microbes of *M. micrantha* were able to reduce the conversion of nitrate nitrogen into gaseous nitrogen oxides, thus preventing the loss of available nitrogen.”.

L322: "was all" > were

It has been revised.

L322: "these evidences" > these evidence (no S)

It has been revised.

All this message on the effect of STL in interesting, but there is no evidence of the allelopathic effect of these STL. Relevant with the message?

The allelopathy of STL has been proved in previous studies (Shao et al., 2005; Huang

et al., 2008; Piyasena et al., 2013). For example, Shao et al. reported that the sesquiterpenoids from *M. micrantha* inhibited both germination and seedling growth of tested species with deoxymikanolide possessing the strongest phytotoxicity. In a bioassay against lettuce (Lectuca sativa L.), deoxymikanolide reduced radicle elongation at low concentration (IC50 = 47 microg/ml); dihydromikanolide showed a weaker effect (IC50 = 96 microg/ml). Deoxymikanolide caused yellowish lesions at the root tips of lettuce at a concentration of 50 microg/ml, and a 250 microg/ml solution killed lettuce seedlings (Shao, 2005). Huang et al reported that deoxymikanolide and dihydromikanolide showed the inhibitory effect on seeding growth of B. parachinensis with I50 value at 1.08 and 0.88 mM. Deoxymikanolide and dihydromikanolide inhibited on shoot growth with the I50 values in the assay being 0.04 and 0.05 mM (Huang, 2008)

In this manuscript, we provide possible synthesis pathways of STL and candidate genes.

References:

1 Shao, H., Peng, S., Wei, X., Zhang, D. & Zhang, C. Potential Allelochemicals from an Invasive Weed Mikania micrantha H.B.K. Journal of Chemical Ecology 31, 1657-1668, doi:10.1007/s10886-005-5805-0 (2005).

2 Huang, H., Ye, W., Wei, X. & Zhang, C. Allelopathic potential of sesquiterpene lactones and phenolic constituents from Mikania micrantha H. B. K. Biochemical Systematics & Ecology 36, 867-871 (2008).

3 Piyasena, K. G. N. P. & Dharmaratne, H. R. W. Allelopathic activity studies of Mikania scandens. Natural Product Research 27, 76-79 (2013).

Discussion:

L327-330: not informative

We have removed it from revised version.

L330-332: WGD not a scoop, already known that it was shared by most Heliantheae.

We have removed it from revised version.

L333-334: redundant with the results section.

We have removed it from revised version.

L334: double T

It has been revised.

L335 with "a" published genome

It has been revised.

L336: upper case in Asterids

It has been revised.

L336-337: how?

We have removed it from revised version.

L344-L345: "most than ..." only if the set of other species chosen in the stem photosynthesis experiment was representative. It is not possible to judge it because this choice was not justified.

We agreed with the reviewer’s comment. We changed the sentence from “Moreover, the stem of *M. micrantha* also has a greater photosynthetic capacity than that of most

plant species” to “Moreover, the stem of *M. micrantha* also has a high photosynthetic capacity”.

L348-349: what do you mean by "transferring enzymes from C4 to C3 plants"?

Thanks for the review’s question. Previous studies reported that photosynthetic efficiency could increase by modification of the photosynthetic pathway, such as introducing an intact maize gene for one of the key enzymes in C4 photosynthesis, PEPC, into rice (Ku et al.,1999) and the alternative photorespiratory pathways (South et al., 2019), implying a photosynthetic pathway by similar CAM at night in C3 crop could be used to improve their carbon fixation capacity through a modified photosynthetic pathway in the future.

References:

1 Ku MS, Agarie S, Nomura M, Fukayama H, Tsuchida H, Ono K, Hirose S, Toki S, Miyao M, Matsuoka M. High-level expression of maize phosphoenolpyruvate carboxylase in transgenic rice plants. Nat Biotechnol 17, 76-80 (1999).

2 South PF, Cavanagh AP, Liu HW, Ort DR. Synthetic glycolate metabolism pathways stimulate crop growth and productivity in the field. Science 363, (2019).

L352-353: this sentence has no verb. remove the ", which "?

We removed the “, which”.

L356: "could not only promoted" > "could not only promote". What is dissimilatory?

It has been revised.

“dissimilatory nitrogen reduction” should be “dissimilatory nitrate reduction to ammonium”.

L360-364: is this biosynthesis pathway for STL different than in other plants? If not it is not "new".

This biosynthesis pathway of STL was found in other species, but it is found in Mikania micrantha for the first time.

Figure 1:

L423: missing space

It has been revised.

2c: at the scale it is very difficult to visualize the synteny relationships between the two genomes. Zoom on several interesting chromosomes and put the complete dotplot in supplementary?

We agreed with the reviewer’s comment. We selected two chromosomes to display syntenic relationship between *M. micrantha* and sunflower. And Dot plot of syntenic orthologues in genome level was moved to supplementary.

Figure 3:

a) not obvious from this table than CAM genes are enriched.

The total genes number of *M. micrantha* (51 genes) in the CAM pathway were more than that in pineapple (34 genes) and sunflower (37 genes).

e) L443: add "proposed pathway", be careful on the conclusions.

Thanks for the reviewer’s valuable suggestions. We have added the “proposed” in this sentence.

REVIEWERS' COMMENTS:

Reviewer #1 (Remarks to the Author):

1. Grammar and style should be re-checked.

2. In line 242 "HaH8H" should be replaced by "HaG8H"

3. Supplementary Figure 24.
Resolution and quality of the pictures should be improved, and trichomes should be shown in more detail.

Reviewer #2 (Remarks to the Author):

The authors have addressed my previous concerns.

Reviewer #3 (Remarks to the Author):

I have read the revised version of the manuscript entitled "Mikania micrantha, 'mile-a-minute', genome provides insights into the molecular mechanism of rapid growth. The authors have adressed the reviewer comments, improving clarity, toning down conclusions when necessary and adding experiments to better support their claims.
I think that this is now a very interesting manuscript. It presents a high-quality reference genome that will be useful to many comparative analyses in Asteraceae, the largest family of flowering plants. The biological insights on the molecular bases of invasion are interesting, new and convincing and will interest a large readership. I therefore recommend the publication of this manuscript in Nature Communications.

REVIEWERS' COMMENTS:

Reviewer #1 (Remarks to the Author):

1. Grammar and style should be re-checked.

We have re-checked the grammar and style in the manuscript and modified some unreasonable statements, including:

The sentence “Here, we generated a reference genome of M. micrantha and performed an integrated analysis of transcriptomic, metabolomic and metagenomic data to explore the genetic processes and molecular mechanisms of its rapid growth.“ was changed to “Here, we generate a reference genome of M. micrantha and performe an integrated analysis of transcriptomic, metabolomic and metagenomic data to explore the genetic processes and molecular mechanisms of its rapid growth. “

The sentence “These evidence indicated that the STLs of soil may play an important role in enriching microorganisms, which in turn accelerating the nutrient cycling (Figure 6d)” was changed to “These evidences indicated that the STLs of soil may play an important role in enriching microorganisms, which in turn accelerating the nutrient cycling (Figure 6d)”.

The sentence “The anthocyanin contents were markedly reduced in the defoliation treatment because the expression of anthocyanin synthesis pathway genes in this treatment was significantly downregulated compared with that in the control.” Was changed to “The anthocyanin contents were markedly reduced in the defoliation treatment because the expression of anthocyanin synthesis genes in this treatment were significantly downregulated compared with that in the control.”.

In addition, CO2, C3 and C4 were changed to CO_2, C_3 and C_4.

2. In line 242 "HaH8H" should be replaced by "HaG8H"

It has been revised.

3. Supplementary Figure 24.

Resolution and quality of the pictures should be improved, and trichomes should be shown in more detail.

The resolution and quality of the Supplementary Figure 24 were improved, and we also showed more details of trichomes in this picture.

Reviewer #2 (Remarks to the Author):

The authors have addressed my previous concerns.

Reviewer #3 (Remarks to the Author):

I have read the revised version of the manuscript entitled "Mikania micrantha, 'mile-a-minute', genome provides insights into the molecular mechanism of rapid growth. The authors have adressed the reviewer comments, improving clarity, toning down conclusions when necessary and adding experiments to better support their claims.

I think that this is now a very interesting manuscript. It presents a high-quality reference genome that will be useful to many comparative analyses in Asteraceae, the largest family of flowering plants. The biological insights on the molecular bases of invasion are interesting, new and convincing and will interest a large readership. I therefore recommend the publication of this manuscript in Nature Communications.
